# Discerning asthma endotypes through comorbidity mapping

**DOI:** 10.1038/s41467-022-33628-8

**Published:** 2022-11-07

**Authors:** Gengjie Jia, Xue Zhong, Hae Kyung Im, Nathan Schoettler, Milton Pividori, D. Kyle Hogarth, Anne I. Sperling, Steven R. White, Edward T. Naureckas, Christopher S. Lyttle, Chikashi Terao, Yoichiro Kamatani, Masato Akiyama, Koichi Matsuda, Michiaki Kubo, Nancy J. Cox, Carole Ober, Andrey Rzhetsky, Julian Solway

**Affiliations:** 1 Department of Medicine, University of Chicago, Chicago, IL 60637 USA; 2 Institute of Genomics and Systems Biology, University of Chicago, Chicago, IL 60637 USA; 3 Shenzhen Branch, Guangdong Laboratory of Lingnan Modern Agriculture, Genome Analysis Laboratory of the Ministry of Agriculture and Rural Affairs, Agricultural Genomics Institute at Shenzhen, Chinese Academy of Agricultural Sciences, Shenzhen, Guangdong 518120 China; 4 Department of Medicine and Vanderbilt Genetics Institute, Vanderbilt University Medical Center, Nashville, TN 37232 USA; 5 Department of Human Genetics, University of Chicago, Chicago, IL 60637 USA; 6 Department of Genetics, Perelman School of Medicine, University of Pennsylvania, Philadelphia, PA 19104 USA; 7 RIKEN Center for Integrative Medical Sciences, Yokohama, 230-0045 Japan; 8 Clinical Research Center, Shizuoka General Hospital, Shizuoka, 420-8527 Japan; 9 Department of Applied Genetics, The School of Pharmaceutical Sciences, University of Shizuoka, Shizuoka, 422-8526 Japan; 10 Department of Computational Biology and Medical Sciences, Graduate school of Frontier Sciences, The University of Tokyo, Tokyo, 108-8639 Japan; 11 Department of Ophthalmology, Graduate School of Medical Sciences, Kyushu University, Fukuoka, 812-8582 Japan; 12 Committee on Genomics, Genetics, and Systems Biology, University of Chicago, Chicago, IL 60637 USA

**Keywords:** Classification and taxonomy, Asthma

## Abstract

Asthma is a heterogeneous, complex syndrome, and identifying asthma endotypes has been challenging. We hypothesize that distinct endotypes of asthma arise in disparate genetic variation and life-time environmental exposure backgrounds, and that disease comorbidity patterns serve as a surrogate for such genetic and exposure variations. Here, we computationally discover 22 distinct comorbid disease patterns among individuals with asthma (asthma comorbidity subgroups) using diagnosis records for >151 M US residents, and re-identify 11 of the 22 subgroups in the much smaller UK Biobank. GWASs to discern asthma risk loci for individuals within each subgroup and in all subgroups combined reveal 109 independent risk loci, of which 52 are replicated in multi-ancestry meta-analysis across different ethnicity subsamples in UK Biobank, US BioVU, and BioBank Japan. Fourteen loci confer asthma risk in multiple subgroups and in all subgroups combined. Importantly, another six loci confer asthma risk in only one subgroup. The strength of association between asthma and each of 44 health-related phenotypes also varies dramatically across subgroups. This work reveals subpopulations of asthma patients distinguished by comorbidity patterns, asthma risk loci, gene expression, and health-related phenotypes, and so reveals different asthma endotypes.

## Abstract (web summary)

Asthma is a heterogeneous, complex syndrome that arises in individuals with various genetic and exposure variations. Here, the authors show that disease comorbidity patterns can serve as a surrogate for these variations, and identify asthma endotypes distinguished by comorbidity patterns, asthma risk loci, gene expression, and health-related phenotypes.

## Introduction

Asthma is a prevalent, debilitating, and expensive condition that affects about 30 million Americans and about 300 million people worldwide^1^. It is a heterogeneous complex syndrome that undoubtedly represents an amalgam of multiple distinct “diseases,” each stemming from a different constellation of genetic variations, environmental exposure histories, and molecular mechanisms that results in a generally similar clinical diathesis. The heterogeneous nature of asthma is evidenced in its varying clinical presentations, spectrum of airway inflammation, and differences in individual responses to asthma treatments^2–14^. Moreover, the risk loci discovered by genome-wide association studies (GWASs) in very large samples of individuals with “asthma” do not account for all of the genetic risks for asthma, indicating that genetic variants in additional loci are yet to be discovered. These missing loci likely include those that contribute to specific subtypes of asthma – but acquiring sufficiently large numbers of individuals with detailed phenotypic and genetic data to study the genetics of asthma subgroups has been challenging.

We and others have performed studies of genetic variation, gene expression, and DNA methylation in an attempt to identify patient subpopulations based on pathogenetic mechanism (“endotypes”)^15–21^, but such studies require direct patient contact and invasive procedures to obtain airway cells, thereby limiting the number of participants.

The extreme heterogeneity of asthma makes it paradigmatic of many complex common diseases. Consequently, designing an approach to distinguish asthma patient subgroups within which individuals share common pathogenetic mechanisms could provide a beacon for parallel approaches in other complex common diseases of the lung (e.g., COPD, interstitial lung disease) or of other organ systems (e.g., hypertension, congestive heart failure, type 2 diabetes).

In this work, we describe a novel approach based on the hypothesis that individuals with different asthma endotypes might be separable based on the other accompanying (non-asthma) diseases they have. Our reasoning is as follows: Each comorbid disease category (e.g., cardiovascular disease, gastrointestinal disease, or breast cancer) is characterized by sets of variations across many genes and sets of exposures (e.g., neighborhood environment, infections, toxins, *in utero*, experiential), behaviors, and traumas that together predispose to diseases in the category^22–27^. Thus, comorbid diseases altogether can be considered a “surrogate” for a corresponding broad genetic and exposure landscape. It seemed likely to us that the asthma diathesis that develops in individuals with one of these broad genetic/exposure landscapes may well have a different pathophysiological basis compared to other asthmatic individuals, whose asthma arises in a very different genetic/exposure landscape. The endotypes of asthmatic individuals from such different landscapes may manifest in unique sets of asthma risk loci and distinct phenotypic characteristics. In this study, we tested this hypothesis.

## Results

### Developing a workflow for asthma subgroup identification

To identify asthma subgroups with distinct comorbidity patterns from a collection of diagnosis records, we applied a “topic modeling” approach^28–34^, inspired by natural-language processing (NLP). In essence, identifying asthma subgroups can be considered as the same task as extracting “topics” (such as “US politics” or “biotechnology news”) from a collection of newspapers, if the following analogies are made: (i) A disease code is a “word;” (ii) A patient’s diagnosis record that contains disease codes (each with its respective abundance) is a “sentence” that consists of words (with words possibly repeated); (iii) A large collection of patient-specific diagnosis histories is a “collection of sentences”; and (iv) An asthma subgroup as defined by a specific distribution of co-occurring diseases (i.e., a comorbidity pattern) is a “topic” (i.e., a probability distribution over words). Specifically, we implemented a Hierarchical Dirichlet Process (HDP) model^35,36^, originally proposed for unsupervised clustering of large collections of texts, such as news articles. In our version of implementation, we treat chronologically ordered clinical histories of individual patients as sentences. In this representation, natural-language words map to disease diagnostic codes (a “text”), and a large collection of patient histories maps to “text corpus.” The underlying generative probabilistic model of data is built on formalism of a stochastic Dirichlet process. In this formalism, each disease subtype is generated by a unique Dirichlet process, and Dirichlet processes for individual disease subtypes share a base distribution which itself is drawn from a Dirichlet process. The HDP modeling automatically determined the optimal number of subgroups through a nonparametric Bayesian model selection approach (see Methods).

**Fig. 1:**
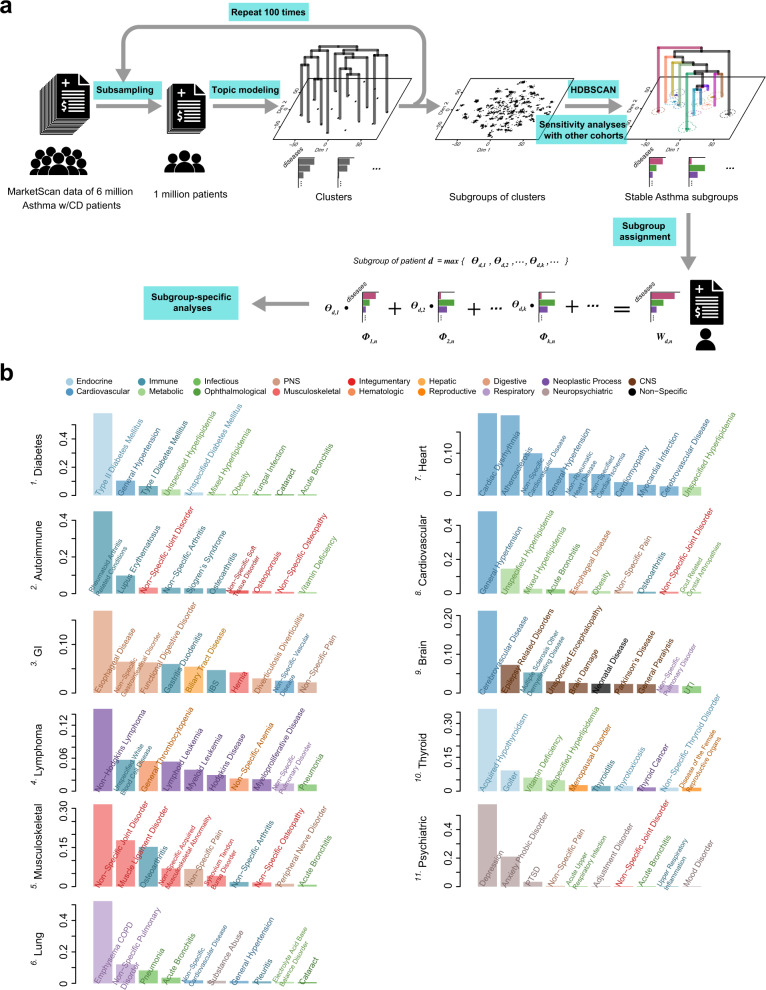
Identification of asthma subgroups through topic modeling. **a** Flowchart of asthma subgroup identification. The MarketScan data includes around six million asthma patients who have at least one comorbid disease (CD). To enable the estimation of sample statistics, we randomly selected one million patients and applied topic modeling to obtain comorbidity clusters (one cluster is projected as one point in the *t-SNE* plot). This procedure was repeated 100 times, generating a large collection of clusters shown as thousands of scattered points in the *t-*SNE projection. We used this *t*-SNE low-dimensional projection of topics only for visualization purpose, rather than for cluster discovery. With inter-cluster dissimilarity measured by Jensen-Shannon divergence, we applied HDBSCAN to identify stable subgroups of clusters as well as their hierarchies. A potential subgroup was deemed to be a stable “asthma subgroup”, only if it harbored more than 50 cluster points. We also conducted a sensitivity analysis on our identification approach in four additional cohorts, and subsequentially show the eleven subgroups that were commonly found in all the different cohorts above. Then, given the distribution of diagnosis counts shown in an individual’s record, we can express it as a linear combination of the distributions of diagnosis counts as defined in the asthma subgroups, and suggest that the subgroup with the largest assigned coefficient could represent the individual’s record best, therefore “assigning” the individual to this subgroup (${W}_{d,n}$, ${\varPhi }_{k,n}$, and ${\varTheta }_{d,k}$ contain the information about record-diagnosis co-occurrences, subgroup profiles, and assignment coefficients, respectively; see Methods for more details). **b** The top ten frequently occurring diseases in the identified eleven asthma subgroups. A complete and precise definition of an asthma subgroup requires one to specify the frequency distribution of 567 disease groups. For each subgroup, we use a bar plot to show its top ten frequently occurring diseases, and color-code the bars as well as the annotations by the broader categories that the diseases belong to. The *y* axis denotes the normalized occurring frequency of a given disease, and we can see that a subgroup is named after the broader category to which several most frequently occurring diseases belong (see Supplementary Data 1 for the subgroup profiles in detail).

The MarketScan database of diagnosis contains records for over 151 million US residents^37^, covering 567 major groups of diseases suggested by ICD code taxonomy^38,39^. We selected asthma patients aged 15–70 who also had comorbid diseases to construct the “collection of sentences” for modeling. The resulting population was around six million, of which we used records from one million randomly selected individuals each time as input to the HDP modeling, repeating the modeling process for 100 times (see a flowchart in Fig. 1a). A large ensemble of clusters was thus generated, and a cluster therein was essentially a specific frequency distribution of comorbid diseases. Some resulting clusters were similar, while others were not, partially due to the stochastic nature of HDP modeling. The inter-cluster dissimilarity, i.e., dissimilarity between frequency distributions, can be measured by Jensen-Shannon divergence, and we then applied Hierarchical Density-Based Spatial Clustering of Applications with Noise (HDBSCAN)^40–42^ to discern the stable subgroups of recurring clusters from non-recurring ones (outliers). We considered a subgroup to be stable and designated it as an “asthma subgroup,” only if it enclosed more than 50 cluster points (see Methods for parameter selection results). By applying this subgroup-discovery workflow, we identified 22 asthma comorbidity subgroups, each with a unique distribution of 567 disease frequencies. The specific frequency distribution of 567 disease groups defined the “comorbidity pattern” in an asthma subgroup and was quantified collectively by its enclosed clusters. The median values, as well as minima, the first quartiles, the third quartiles, and maxima of the occurring frequencies of diseases in the clusters are shown in Supplementary Data 1 for each subgroup.

Next, we conducted sensitivity analyses on our identification approach using other four different cohorts, including (i) individuals in the MarketScan data who were aged between 15 and 70, but carried at least two asthma codes, (ii) individuals in the MarketScan data who carried at least one asthma code, but were aged between 40 and 70, (iii) individuals in the MarketScan data who not only were aged between 15 and 70 and carried at least one asthma code, but also had at least one type of asthma drug prescriptions, and (iv) individuals enrolled in UK Biobank (UKB). By repeating the exact same procedure as described above, we could re-discover 21, 20, 22, and eleven subgroups out of the original 22, respectively (see Supplementary Data 2–5 and Supplementary Table 2 for the subgroup profiles). For visualization purpose only, the asthma subgroups were projected into a two-dimensional space using the *t-*SNE algorithm^43^, and we show the eleven subgroups that were found in all the different cohorts above in Supplementary Fig. 1b. Supplementary Fig. 1c shows the hierarchical clustering of these subgroups, and for each subgroup, a word cloud summarizes comorbid diseases therein contained and their occurring frequencies (proportional to the font sizes). For easier reference, we labeled each asthma comorbidity subgroup with a serial number and the broader category to which several most frequently occurring diseases belonged (see Fig. 1b for the relative frequencies of top 10 comorbid conditions in each asthma subgroup).

To our knowledge, this is the first such analysis of asthma comorbidity patterns over the entire disease spectrum. Some comorbid conditions identified in the 2007 American National Asthma Education and Prevention Program (NAEPP) guidelines^44^ appear prominent in certain subgroups, such as gastrointestinal disease in subgroup 3 and depression in subgroup 11. More interestingly, some comorbidity associations are novel, such as lymphoma in subgroup 4 and joint disorder in subgroup 5.

### Identifying genetic associations specific to asthma subgroups

Our underlying premise is that each individual’s comorbid diseases arose in a gene-environment background that predisposed to their occurrences. Therefore, comorbidities can serve as surrogates for the various overall gene-environment settings in which different asthma endotypes can arise. By comparing patients with and without asthma who all share the same comorbidity pattern (as defined in an asthma comorbidity subgroup), we studied asthma risk genes in a subgroup-specific manner. For this purpose, we selected unrelated individuals of white British background and with high-quality genotyping from the UKB^45^ as a discovery cohort, including 44,383 asthma cases and 260,715 non-asthma controls (see Table 1 and Supplementary Table 4). With the profiles of subgroups comprehensively defined, we could assign any individual to the most appropriate asthma subgroup that best matched an individual’s complete collection of disease diagnoses and respective occurring frequencies (see Methods).

**Table 1:** Descriptions of used databases

Database	Ethnicity	Total sample size (asthma case count)	Male percentage	Median age^g^	Usage
MarketScan (select age ≥15)^a^	White (78.3%), Black (14.5%)^f^	84,315,387 (6,048,247)	44.8%	41 (29–53)	Asthma subgroup identification
UK Biobank^b^	British white	305,098 (44,383)	45.7%	59 (51–64)	GWAS discovery, and phenotype association analysis
	Irish white	22,600 (3,186)	41.9%	57 (49–63)	Replication of GWAS findings via meta-analysis
	African, Caribbean	6,833 (998)	40.5%	51 (46–58)	
BioVU^c^	White	16,060 (1,668)	50.3%	61 (51–71)	
BioBank Japan^d^	East Asian	194,413 (3,368)	54.1%	65 (55–73)	
UChicago RNAseq^e^	White (37.1%), Black (58.6%)	70 (42)	32.9%	38 (27–50)	Differential gene expression validation

^a^The MarketScan insurance claims database in the US, including diagnosis records.

^b^National health database in the UK, including diagnosis records and genotype data.

^c^Patient-based registry of Vanderbilt University Medical Center, including diagnosis records and genotype data.

^d^Patient-based registry in Japan, including diagnosis records and genotype data.

^e^RNAseq transcriptome profiles of bronchial epithelial cells of patients enrolled in the University of Chicago.

^f^Imputed percentage based on county-level distributions of race.

^g^Values in parentheses are interquartile ranges given in years.

First, we performed a larger GWAS of asthma by comparing asthma cases and non-asthma controls among all individuals with any comorbid diseases (“any-CDs group”)^46^. We observed 103 independent loci of genome-wide significance (*p* < 5 × 10^−8^), 13 of which were not previously reported in the NHGRI-EBI GWAS catalog database^47^.

**Fig. 2:**
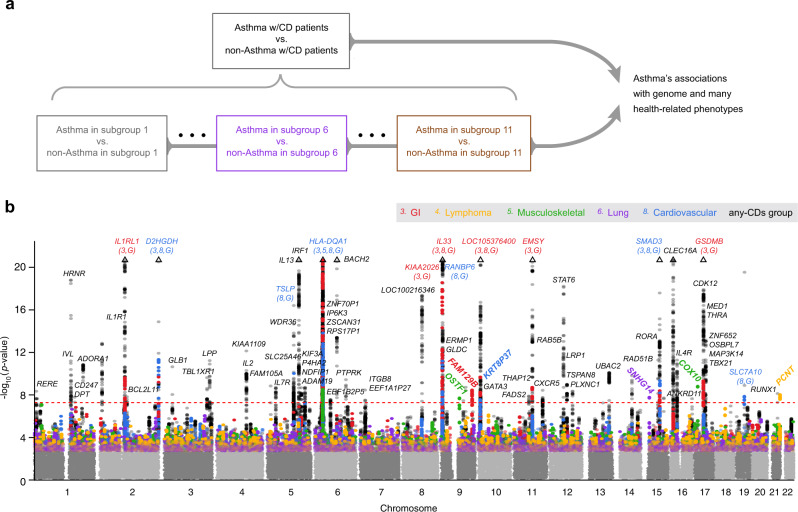
Genome-wide significant associations with asthma. **a** Study design for association analyses. Starting with the general population who may have any comorbid diseases (the any-CDs group) in UK Biobank, we were able to assign an individual with 1 of the 11 asthma subgroups that were found in UK Biobank. Then, we performed GWASs to identify asthma risk loci for the any-CDs group and for each subgroup individually (by comparing asthma cases against non-asthma controls within each subgroup). **b** GWAS Manhattan plots. This figure overlays GWAS results from the any-CDs group (in black) and from five selected subgroups (in multi-colors) that contained genome-wide significant asthma risk loci, including subgroups 3 “GI,” 4 “Lymphoma,” 5 “Musculoskeletal,” 6 “Lung,” and 8 “Cardiovascular.” All the association *p* values are shown on a –log_10_ scale on the *y* axis, and genomic locations are shown on the *x*-axis. The threshold of genome-wide significance (5 × 10^−8^) is indicated as a horizontal dashed line in red. Triangles at top indicate SNPs that have a higher –log_10_(*p* value) than shown. In addition, we annotate genome-wide significant loci with the names of their nearest genes, and in the case where a gene is commonly found in multiple subgroups and in the any-CDs group, the subgroup serial numbers and letter “G” are written, respectively, in parentheses under the gene name. In particular, we highlight the genes nearest to the six subgroup-specific loci by rotating their names with an angle of 45 degrees. More details can be found in Supplementary Table 1.

Second, we assigned asthma cases and non-asthma controls to their comorbidity subgroups, forming case and control subgroup pairs that shared the same comorbidity patterns (see Fig. 2a). Within each of the eleven subgroups that were re-discovered in UKB, we carried out a GWAS of asthma, identifying 14 loci that were also found in the initial larger GWAS analysis, plus six additional loci that conferred asthma risk in one subgroup, but not in the other subgroups or in the initial asthma GWAS. We show Manhattan plots of these results in Fig. 2b and annotate significant loci with their nearest genes (see Supplementary Table 1 for the complete loci information).

For example, in addition to being significantly associated with asthma in the initial larger GWAS, variants near *IL1RL1*, *KIAA2026*, *EMSY*, and *GSDMB* were also associated with asthma in subgroup 3 “GI;” variants near *TSLP*, *RANBP6*, and *SLC7A10* in subgroup 8 “Cardiovascular;” and variants near *D2HGDH*, *HLA-DQA1*, *IL33*, and *SMAD3* in subgroups 3 and 8. The lead SNPs at the six subgroup-specific loci include rs11144271 (near *OSTF1*, *p* = 2.50 × 10^−8^) and rs113757163 (near *COX10*, *p* = 1.58 × 10^−9^) in subgroup 5 “Musculoskeletal,” rs2249851 (in *FAM129B*, *p* = 3.30 × 10^−9^) in subgroup 3 “GI,” rs76225731 (in *SNHG14*, *p* = 3.66 × 10^−8^) in subgroup 6 “Lung,” rs117262476 (in *PCNT*, *p* = 1.46 × 10^−8^) in subgroup 4 “Lymphoma,” and rs2765400 (near *KRT8P37*, *p* = 2.56 × 10^−8^) in subgroup 8 “Cardiovascular.” Five of the six subgroup-specific loci, except for the last one (rs2765400), were novel, meaning, never reported in any asthma GWASs before. If a Bonferroni correction is further applied to adjust the twelve GWASs in total (eleven subgroups and a general asthma population), and the adjusted genome-wide significance threshold becomes 4.17 × 10^−9^ (i.e., 5 × 10^−8^/12), then there are two associations that remain significant: rs113757163 near *COX10* and rs2249851 in *FAM129B*.

**Fig. 3:**
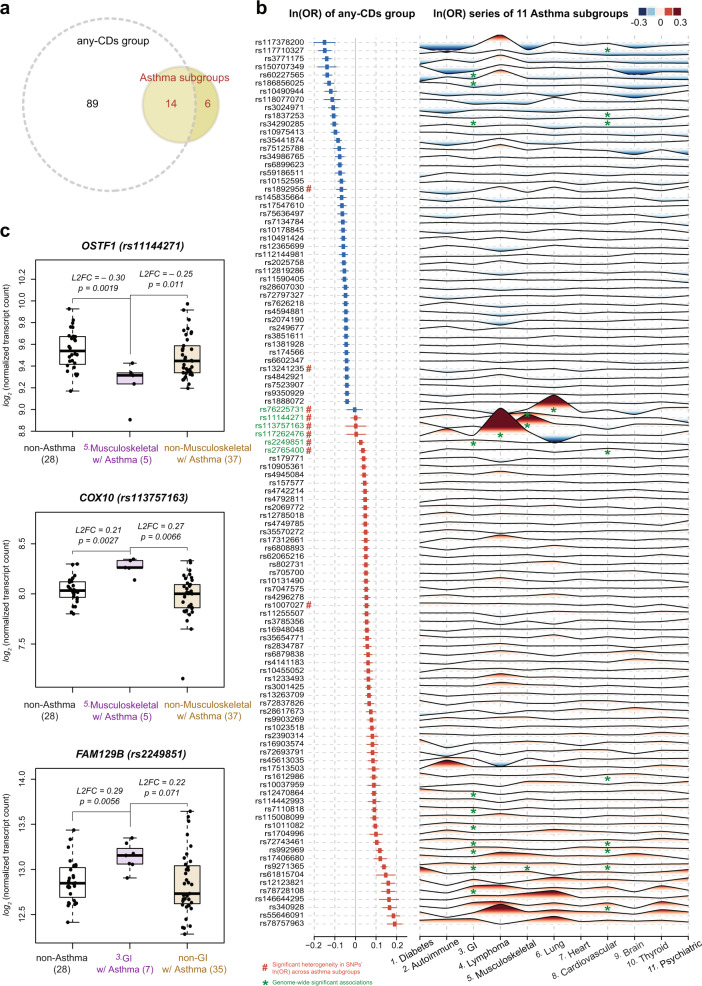
Summary of genome-wide significant loci and differential gene expression. **a** A summary of the significant loci in a Venn diagram. The association analysis by comparing asthma cases and non-asthma controls in the any-CDs group identified 103 independent loci at genome-wide significance level. Similar association analyses within each of the eleven asthma subgroups discovered 20 significant loci, of which 14 were also seen in the any-CDs group, and, interestingly, six more loci were specific to one subgroup only. Altogether there were 109 independent loci identified. **b** Association results for significant loci. The forest plot on the left side summarizes the association results seen in the any-CDs group for the 109 loci, at which the lead SNPs are listed in the first column. Squares denote the effect sizes, i.e., natural logarithm of odds ratios or ln(OR), and horizontal lines are the 95% confidence intervals. From top to bottom, the effect sizes are in ascending order, from negative (in blue) to positive values (in red). The wave-like plot on the right side displays a series of effect sizes seen in the eleven subgroups that can be found in UK Biobank for each of the 109 SNPs. The subgroup names are labeled along the horizontal axis, while for each of the 109 SNPs that are displayed along the vertical axis, its effect size is represented as a peak in the red shade if it is positive, and as a trough in blue shade if negative. The absolute value of the effect size is proportional to the height (or depth) of the peak (or trough), and is also color-coded. All the genome-wide significant associations between SNPs and subgroups are marked with green asterisks, and in particular, the six SNPs that are specific to one subgroup only are highlighted in green in the first name column. In addition, the heterogeneity of per-locus effect sizes across the eleven subgroups was assessed through a Cochran’s Q test, finding nine loci with evidence of significant heterogeneity in effect sizes (indicated with # symbols in red after the respective SNP names in the first column). See Supplementary Data 6 for the association results in detail and Supplementary Fig. 5 for the numbers of allocated cases and controls in each subgroup. **c** Differential gene expression. For three of the subgroup-specific SNPs, we confirmed the differential expression of their nearby genes (i.e., *OSTF1*, *COX10*, and *FAM129B*), using an independent dataset of bronchial epithelial transcriptome profiles. The gene *OSTF1*, for example, has significantly lower expression among asthma cases in subgroup 5 “Musculoskeletal”, compared to non-asthma controls and asthma cases in other subgroups (see the *x* axis labels and respective sample sizes in parentheses). The *y*-axis shows the normalized transcript count on a log_*2*_ scale, i.e., log_*2*_[(transcript count+0.5)/size factor]; the minimum, the first quartile, the median, the third quartile, and the maximum of *OSTF1* for non-asthma controls are 9.17, 9.42, 9.54, 9.67, and 9.93, for asthma cases in subgroup 5 are 8.91, 9.24, 9.31, 9.34, and 9.43, and for asthma cases in other subgroups are 9.20, 9.34, 9.45, 9.59, and 9.97; these values of *COX10* for non-asthma controls are 7.80, 7.97, 8.03, 8.12, and 8.30, for asthma cases in subgroup 5 are 8.14, 8.26, 8.26, 8.33, and 8.35, and for asthma cases in other subgroups are 7.15, 7.86, 8.00, 8.09, and 8.33; these values of *FAM129B* for non-asthma controls are 12.41, 12.71, 12.85, 13.01, and 13.44, for asthma cases in subgroup 3 are 12.91, 13.06, 13.15, 13.23, and 13.35, and for asthma cases in other subgroups are 12.28, 12.62, 12.73, 13.04, and 13.64). The mean log_2_ fold changes (L2FC) of *OSTF1* in subgroup 5 of asthma cases were −0.30 (two-sided Wald statistic *p* value = 0.0019) and −0.25 (*p* value = 0.011), when compared to non-asthma controls and asthma cases in other subgroups, respectively. The other comparisons show that bronchial epithelial cell expression of *COX10* in subgroup 5 “Musculoskeletal” and *FAM129B* in subgroup 3 “GI” are significantly higher, compared to non-asthma controls and their respective asthma cases in other subgroups.

In summary, we identified a total of 109 independent loci, representing the union of all genome-wide significant asthma risk loci found in any of the GWASs in our study (Fig. 3a). We investigated the heterogeneity in the effect sizes of the lead SNPs at these 109 loci across the eleven subgroups, using a Cochran’s Q test^48^. This revealed significant heterogeneity at nine loci (marked with red # symbols in Fig. 3b), which included all the six subgroup-specific loci (Supplementary Data 6). To validate these discoveries, we conducted a multi-ancestry meta-analysis^49–53^ of four additional cohorts, including two subsets from UKB that were not included in the initial GWAS (a cohort of white Irish and any other white background, and a cohort of African, Caribbean and any other backgrounds associated with recent African descent, respectively), a European ancestry subset of BioVU from the Vanderbilt University Medical Center^54,55^, and an East Asian ethnic group from BioBank Japan (BBJ)^56–58^. After multiple testing correction, there remained 61 associations (involving 52 loci) successfully replicated, consisting of 49 (involving 49 loci) from the any-CDs group and twelve (involving ten loci) from subgroups. The latter, in particular, included three subgroup-specific loci: rs11144271 (near *OSTF1*), and rs113757163 (near *COX10*), both in subgroup 5 “Musculoskeletal,” and rs2765400 (near *KRT8P37*) in subgroup 8 “Cardiovascular” (see Supplementary Data 7 for summary statistics).

Third, using transcriptome data from bronchial epithelial cells (BECs) obtained by bronchoscopy from a small number of patients (42 asthma cases and 28 non-asthma controls) at the University of Chicago^18,59^, we checked for possible differential expression of the genes nearest to the six subgroup-specific loci. Based on the available diagnosis information, we assigned the 42 asthma cases into comorbidity subgroups; only subgroups 5 and 3 involving three genes (*OSTF1*, *COX10*, and *FAM129B*) contained five or more individuals, and were included in these analyses. We formularized gene transcript counts using a generalized linear model of the negative binomial family^60^ with age, sex, and ethnicity included as covariates. We compared asthma cases within each group to two reference (control) groups: 28 non-asthmatic individuals, and the asthma cases that fell into those subgroups other than the one being tested (see Methods). As shown in Fig. 3c, *OSTF1* expression was significantly reduced while *COX10* was overexpressed in asthmatics in subgroup 5 “Musculoskeletal”, compared to the expression levels in the non-asthma controls or in the asthma cases not in subgroup 5. The expression of *FAM129B* was significantly higher among the cases in subgroup 3 “GI” compared to either reference group. In addition, we used both the HaploReg v4.1^61^ and the Genotype-Tissue Expression project (GTEx)^62^ databases to determine whether the associated SNPs were also expression quantitative trait loci (eQTLs). We found that rs11144271 is an eQTL for *OSTF1* in whole blood (*p* = 2.5 × 10^−29^), and rs2249851 is an eQTL for *FAM129B* in cultured fibroblasts (*p* = 3.1 × 10^−22^), in whole blood (*p* = 1.3 × 10^−6^), in pituitary (*p* = 3.6 × 10^−5^), and in tibial artery (*p* = 6.1 × 10^−5^). Admittedly, differential expression analysis and functional validation of additional genes will be needed to infer causal associations between the genes and subgroup-specific asthma risk.

Next, we performed pathway enrichment analyses based on the full subgroup association results. Asthma subgroups indeed show distinct sets of enriched biological pathways/processes, for example, keratinocyte differentiation (*p* = 7.52 × 10^−16^) and the regulation of leukocyte proliferation (*p* = 5.27 × 10^−7^) in subgroup 3 “GI”, and keratinocyte differentiation (*p* = 4.40 × 10^−19^) and epidermal cell differentiation (*p* = 2.24 × 10^−14^) in subgroup 8 “Cardiovascular”. These enriched biological pathways or processes could potentially inform subgroup-specific asthma pathogeneses. Complete listings for all the eleven asthma subgroups can be found in Supplementary Table 3.

### Asthma associations with health-related phenotypes differ across subgroups

If the identified subgroups reflect true endotypes, then there should be health-related phenotypes (e.g., measurable clinical differences) that differentially associate with asthma among comorbidity subgroups and possibly suggest distinct pathogenetic mechanisms. To test this, we leveraged the phenotypic data in the UKB resource^45^, and focused on a total of 140 different phenotypes that measured ten health-related categories, including spirometry, blood count, blood biochemistry, urine biochemistry, early life factors, anthropometry, addictions, diet, physical activity, and local environment. We focused these studies on the same cohort as we used for the GWAS discovery: unrelated individuals of white British ethnicity with available diagnosis records. We implemented a multivariate adaptive shrinkage (mash) method^63^ to assess the heterogeneity of the associations across subgroups by benchmarking against the larger group with any comorbidities (as benchmarks; see Methods).

**Fig. 4:**
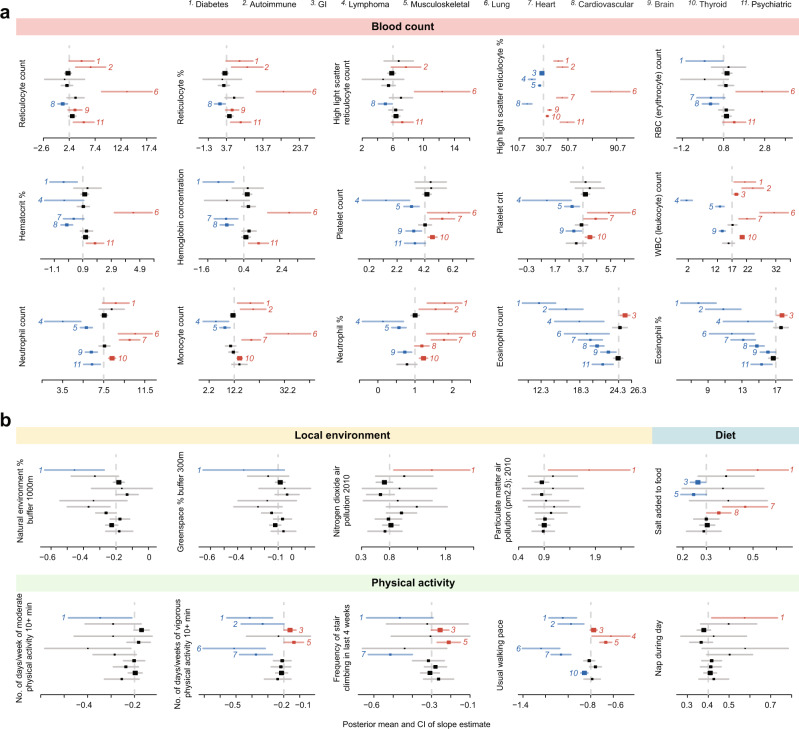
Differential asthma associations with health-related phenotypes across subgroups. A total of 10 different categories of health-related phenotypes (140 different measurements in total) were subjected to phenotype association analysis (see Methods for technical details and Supplementary Data 11 for the numbers of allocated cases and controls in each subgroup). We first computed phenotypes’ slope estimates of asthma associations within each subgroup and in the any-CDs group. The direction and strength of the association are characterized by the sign and absolute value of the slope, respectively. **a** Heterogeneous slope estimates related to blood count. We assessed the heterogeneity in these slope estimates across subgroups for each phenotype, and benchmarked against the slope value for that phenotype in the any-CDs group. Each phenotype is presented as a meta-plot, which shows the posterior means (as squares) and 95 percent confidence intervals (as error bars) of the slopes from subgroups 1 to 11 that were also discovered in UK Biobank (displayed from top to bottom). Slope estimates that are significantly less positive than the any-CDs group benchmark (marked by a vertical dashed line) are shown in blue, while those that are significantly more positive are shown in red; the respective subgroup numbers are also shown for significantly different subgroups. For example, subgroup 6 “Lung” exhibits many red-blood-cell-related phenotypes that are in significantly stronger associations with asthma likelihood than appear for the general population in the any-CDs group. **b** Heterogeneous slope estimates related to the local environment, diet, and physical activity. In the same fashion as shown in **a**, we display the meta-plots of the phenotypes in the categories of the local environment, diet, and physical activity. A distinct pattern of these phenotypes distinguishes subgroup 1 “Diabetes,” in which stronger associations of greenspace, air quality, salt intake, and exercise are evident.

The first step was to examine asthma associations for each phenotype in the ten categories, in each subgroup as well as in the larger group. We used the slope estimate of an association, i.e., increased likelihood of asthma with respect to increasing or decreasing value of the phenotypic feature, to denote the association’s direction (by the sign of the slope) and strength (by the absolute value of the slope; see Supplementary Data 11). The analysis revealed 44 phenotypes associated with asthma differentially across subgroups (see Supplementary Data 12 for the estimates of the slope differences after benchmarking against any-CDs group). These subgroup-specific differential associations are highlighted in color (blue signifies less positive than the benchmark, red signifies more positive than the benchmark) in the meta-plots in Fig. 4 and Supplementary Fig. 7, which show the posterior means and variances of the association slopes. This analysis demonstrated that clinically relevant phenotypes indeed varied across subgroups, with some suggesting potential subgroup-specific endotypic mechanisms (see Discussion).

Finally, we have collated summary statistics of relevant health-related phenotypes available in MarketScan and UKB data (including white blood cell counts, spirometry measurements, body mass index, smoking status, age of asthma onset, and asthma medications), and compare them in a subgroup-specific manner in Supplementary Tables 5–9. As shown in Supplementary Table 6, the abnormalities of spirometry measures (including reductions of predicted forced vital capacity (FVC), forced expiratory volume in one second (FEV_1_), peak expiratory flow (PEF), and the ratio of FEV_1_ to FVC (FEV_1_/FVC) are greatest in subgroup 6 “Lung” and are modest in subgroup 5 “Musculoskeletal;” Supplementary Table 9 shows that inhaled steroid combinations with long-acting beta agonists or antibody inhibitors, both of which medication categories are usually prescribed for more severe asthma, have the largest fraction of users in the “Lung” subgroup and less than half that fraction in the “Musculoskeletal” subgroup. Collectively, these suggest that the “Lung” subgroup may comprise individuals with more severe asthma than that experienced by individuals in the “Musculoskeletal” subgroup.

## Discussion

Currently, the most widely adopted method of asthma classification is based on severity, defined by the level of symptoms, lung function, and rescue bronchodilator use^44^. Asthma has also been classified by onset age: early- and late-onset^64^; by the presence or absence of allergic sensitization: atopic and nonatopic^65,66^; by the level of symptom control: controlled, partly controlled, and uncontrolled^67^; or, more recently by the co-occurrence of other medical conditions like obesity^68,69^, rhinosinusitis^70^, and depression^71–73^, which are thought to exacerbate symptoms or even directly contribute to asthma pathogenesis. One problem with the current classifications lies in poor coherence and subjectivity; studies have shown that poor agreement can exist across classification systems^74^, official guidelines, and physician assessment^75^. Additionally, there is increasing evidence that the current classifications can sometimes be too broad to adequately reflect the highly heterogeneous characteristics observed in asthma populations^4,64,76^. In this study, we sought to discover asthma subtypes in a data-driven, probabilistic modeling-based unsupervised way: (i) We gathered large-scale, multi-dimensional datasets, including very large diagnosis records and genotype data originating from multiple countries (US, UK, and Japan), RNA-sequencing profiles (laboratory measurement), and a suite of health-related phenotypic measures (see Table 1 and Supplementary Fig. 5 for a brief summary of all the used datasets); (ii) The workflow and methodologies proposed in this study are a showcase for the benefits from the integration of these multi-dimensional information, and can work as machinery that has general applicability towards the investigation of other complex diseases.

The ever-increasing availability of large-scale administrative medical records has allowed us to find emerging comorbid conditions among asthma patients^77–85^ and should allow the investigation of their adverse effects, including asthma exacerbation^86^, lower quality of life^87,88^, and increased risk of morbidity and mortality^89^. Here, we refer to a comorbidity pattern as a specific distribution of diseases that co-occur with asthma, and hypothesize that such comorbidity patterns, if analyzed systematically from country-scale diagnosis records, can be very informative in dissecting hidden heterogeneity of asthma and guiding asthma endotyping. The rationale for this approach is rooted in the hypothetical deep connection between comorbidity patterns and asthma endotypes. First, genetic factors can predispose an individual to different asthma endotypes as well as to the manifestation of many other co-occurring diseases, in other words, genetic origins are shared. Studies have shown that trait-associated SNPs discovered by previous GWASs are largely pleiotropic, and tend to influence general biological functions contributing to numerous traits^90^. Second, different asthma endotypes and comorbid conditions can also share environmental exposures or even possibly cause one another, promoting the convergence of certain comorbidities. For these reasons, we suggest that comorbidities are effectively working surrogates for gene-environment landscapes that lead to different asthma endotypes, and that different comorbidity subgroups may harbor unique asthma risk loci. In other words, it seemed likely that the additional risk factors for asthma to arise in one gene-environment landscape (as prevails in one comorbidity subgroup) are different from the additional risk factors that make asthma more likely to arise in a different gene-environment landscape (e.g., as prevails in a second comorbidity subgroup). In this study, we tested and confirmed this possibility. Our approach of using comorbid patterns to derive homogeneous endotypes resonates with the previous studies that identified novel disease subtypes and genetic loci through non-random ascertainment of covariates informed by multiple traits and genetics^91,92^. However, this ascertainment could conceivably induce unintended, biased associations^93–95^. In an effort to restrain them, we replicated the genetic risk loci in multiple ethnic cohorts and aggregated the genetic, gene expression, and phenotypic associations that collectively may suggest the heterogeneity existing in asthma.

The subgroup-specific variants that were found significant in GWASs here may point to different pathogenetic mechanisms in asthma endotypes. For example, we identified an association specific to asthma subgroup 5 “Musculoskeletal.” The lead variant was near osteoclast-stimulating factor 1 (*OSTF1*), a gene that interacts with fatty acid binding protein 4 (*FABP4*)^96^, which in turn regulates airway inflammation in experimental asthma^97,98^. *OSTF1* also regulates cell motility^99^, which could be important in bronchial epithelial repair and inflammatory cell trafficking. Another nearby gene specific to subgroup 5, cytochrome C oxidase assembly homolog 10 (*COX10*), regulates T-cell activation and differentiation^100–102^, and so could regulate asthmatic airway inflammation in some way particularly important for this subgroup. Family with Sequence Similarity 129 Member B (*FAM129B*), selective for subgroup 3 “GI,” regulates glycolysis, Ras activation, oxidative stress, apoptosis^103–106^, and more generalized cell processes whose contributions to asthma pathogenesis could take multiple forms. Experimental studies will be required to identify the exact mechanism(s) by which these genes contribute to asthma in a subgroup-specific fashion.

Similarly, unique phenotypic associations also characterize some asthma subgroups. For example, we were struck by the strong positive slope relationships among multiple measures of red blood cell (RBC) production and accumulation in subgroup 6 “Lung,” including reticulocytes, erythrocytes, hematocrit, and hemoglobin (Fig. 4a). Increased RBC production could reflect higher erythropoietin elaboration or sensitivity. Erythropoietin is known to reduce airway remodeling in experimental murine allergic asthma^107^, perhaps inducing the activation of regulatory T cells^108^ through stimulation with TGF-β released from M2 macrophages. However, TGF-β is well known to promote airway smooth muscle differentiation and accumulation^109–112^, and erythropoietin-induced TGF-β secretion could conceivably represent the key pathogenetic contributor that promotes the emergence of asthma in patients with the comorbidity background of subgroup 6, in which COPD is the most frequent comorbid disease. Consistent with this notion, the association between asthma and blood eosinophil count or percentage is significantly weaker in subgroup 6 than in the larger group with any comorbidities, suggesting that Th2-type inflammation may be relatively less important for the development of asthma in this subgroup.

As another example (Fig. 4b), greater likelihood of asthma in subgroup 1 “Diabetes” is related to less greenspace, higher air pollution, higher salt intake, and lower physical activity. Indeed, both greenspace^113^ and air pollution^114^ have been previously linked to asthma prevalence or severity, and these effects are mirrored in the observed slopes for the whole UKB samples analyzed here as well. Greenspace reduces the incidence of elevated interleukin-8 (IL8) in serum^115^, while both NO_2_^116^ and particulate matter air pollution^117,118^ induce IL8 expression in human airway epithelium. High-intensity interval exercise reduces circulating IL8 in both lean and overweight-obese individuals^119^, and while eating higher salt diets, individuals with exercise-induced asthma experienced worsened post-exercise airflow obstruction and had greater induced sputum IL8 concentrations than when eating a low-salt diet^120^. Importantly, IL8 is particularly elevated in the lung secretions of severe asthmatics^121^. In all, the known role of IL8 in asthma and the phenotypic peculiarities of subgroup 1 asthmatics suggest that their asthma may be especially driven by IL8 secretion. Each of these potential subgroup-specific endotypic mechanisms should be explored experimentally. In total, out of the tested 140 health-related phenotypes, there are 44 showing significant heterogeneity across our subgroups of asthma (see Supplementary Fig. 7 for the other significant phenotypes); these might also contain clues about endotypic mechanisms.

Additionally, 182 asthma-associated loci (at the suggestive threshold, *p* < 10^−5^) had significantly larger effect sizes in specific subgroups than in the initial larger GWAS, although these associations did not reach genome-wide significance. Another 73 independent genome regions had similar effect sizes in one or more subgroups as well as the larger group with any comorbidities (see Supplementary Note 2 for details). Understanding these genetic specificities and commonalities, which collectively mapped the genomic landscapes of asthma subgroups, can be critical in discovering new asthma endotypes and in elucidating their distinct or shared molecular etiologies.

Admittedly, disentangling genetic and environmental heterogeneity of asthma is difficult because (i) sample size diminishes quickly in the process of subdividing asthma cases into subgroups; and (ii) asthma-associated polymorphisms tend to have small effect sizes^122–125^. Although a subsampling method (see Supplementary Note 1 and Methods) alleviates these problems to some extent, the detection of genome-wide significant signals was still restricted to several relatively large subgroups. Extending our current work in the future, it may be possible to represent asthma groupings by multi-dimensional, quantitative risk scores: genotypic, phenotypic, or both. Advantages are two-fold: (i) Continuous risk scores would be assigned to asthma cases instead of binary classifications, allowing the samples to be used more effectively and thus providing gains in statistical power, while the central challenge in this regard is how to best incorporate into these analyses the collection of SNPs and genes, and; (ii) Such scores could predict one’s asthma subgroup before the actual onset of the score-predicted comorbidities, and so could lead to a better understanding of their endotype at an earlier age. Another possible extension of our current approach is to allow the intake of dynamic data about disease trajectories or progressions. This extension will likely be valuable, considering that previous studies have shown that the exact timing of specific environmental exposures during critical developmental windows could influence risk trajectories that ultimately trigger asthma^126^, and only the exposures occurring in early life may leave observable signatures^127^. To this end, longitudinal data with a reasonably long period of coverage will be required.

## Methods

All relevant ethical regulations have been followed. This study was approved by the University of Chicago Institutional Review Board, and informed consent was obtained from all research subjects to the work involving transcriptome data of BECs. The study design and conduct complied with all relevant regulations regarding the use of human study participants and was conducted in accordance with the criteria set by the Declaration of Helsinki.

### The US MarketScan Commercial database and topic modeling for asthma subgroup identification

The US MarketScan databases, owned by IBM Watson Health, are a suite of administrative claims-based databases that include inpatient and outpatient claims, medical procedure claims, prescription claims, clinical utilization records, and healthcare expenditures. These data were collected from employers, managed care organizations, health plan providers, and state Medicaid agencies. The covered patient population is mainly composed of relatively more affluent, privately-insured segments of US society^37,128^. Distinct strengths that lie in the MarketScan databases include: (i) comprehensive and high-quality coding of diagnoses, procedures, and drug prescriptions, (ii) large collection of samples that cover over half of the US population, (iii) longitudinal tracking at the individual level, and (iv) full integration of inpatient and outpatient care events, emergency care services and outpatient pharmaceutical data. More than 900 peer-reviewed research articles have been published since the launch of these databases in 1995, and the number of related publications has increased even more rapidly in recent years^129,130^.

In order to identify asthma subgroups in this study, we used one of the US MarketScan databases—the US MarketScan Commercial Claims and Encounters database (US MarketScan data). The US MarketScan data contain the US country-scale collection of diagnosis records for over 151 million unique individuals who were enrolled in the database during the years between 2003 and 2013. We selected those individuals who were aged between 15 and 70, and carried an asthma code with at least one comorbid disease (in addition to asthma). Here, we used 493.00–493.99 (for ICD-9-CM) and J45.0–J45.998 (for ICD-10-CM) as asthma codes. The resulting population was 6,048,247, and we used their diagnosis records to identify comorbidity-based asthma subgroups. Asthma classification based on diagnosis records was pursued using a topic modeling approach, by analogy with Word documents.

In topic modeling, a document can be viewed as a mixture of topics, where a topic is defined as a distribution over a fixed vocabulary, then a topic model describes a probabilistic generative process for the document in two stages: first, to specify the topic proportions, and second, for the generation of each word in the document, to assign a topic according to its specified proportion and draw a word from the corresponding distribution^28–33^.

On the basis of our diagnosis records consisting of International Classification of Diseases versions 9 and 10 (ICD-9 and ICD-10) codes, we only took into account unique ICD codes per day (only keeping unique ICD codes on each day) and then grouped these ICD codes into 567 major groups of disease diagnoses on the basis of their clinical manifestations^38,39^. These 567 disease groups constituted the basic “vocabulary”, which all the records were built on. An asthma subgroup can be analogously defined as a distribution of diseases (other than asthma) that reflects an existing common comorbidity pattern among asthma patients.

After terminology conversion from “document–topic–word” to “diagnosis record–asthma subgroup–diagnosis”, the probabilistic generative process for a diagnosis record (equivalent to a word document in document modeling) also involves two stages: first, to assign subgroup proportions, and second, for the generation of each diagnosis in the record, to choose a subgroup (equivalent to a topic) and to draw a diagnosis (equivalent to a word) within accordingly. In reality, we are dealing with a statistical inference problem: only diagnosis records can be observed, and the goal is to extract the underlying subgroups that are most likely to have generated these data. For this purpose, a Hierarchical Dirichlet Process (HDP) model^35^ was applied, and its C++ implementation is publicly available at the Github repository at https://github.com/blei-lab/hdp^36^. We set the hyperparameter “*max_iter*” (maximal number of iterations) to be 500, which is large enough for the modeling process to converge (based on our initial test runs). Supplementary Fig. 4a shows its basic design: Shaded and unshaded nodes indicate observed and latent variables, respectively; Arrows denote conditional dependencies between variables, and plate notations are used to illustrate repeated sampling steps. For example, the inner plate over ${Z}_{d,n}$ and ${W}_{d,n}$ denotes the repeated sampling of asthma subgroup assignments and diagnoses until ${N}_{d}$ diagnoses are generated for diagnosis record *d*. The plate over ${\varTheta }_{d,k}$ demonstrates the repeated sampling of a distribution over subgroups for each diagnosis record *d* for a collection of *D* records, and the plate surrounding ${\varPhi }_{k,n}$ illustrates the sampling of diagnosis distributions for each subgroup *k* until the total number *K* is reached. Hyperparameters $\alpha$ and $\beta$ define the HDPs which are the distributions over a set of random probability measures over ${\varTheta }_{d,k}$ and ${\varPhi }_{k,n}$, respectively. Therefore, given the observed ${W}_{d,n}$, statistical inference aims to estimate ${\varTheta }_{d,k}$ and ${\varPhi }_{k,n}$^34^. A nonparametric Bayesian approach was implemented to infer these parameters, and the optimal number of subgroups can also be learnt in the process instead of being fixed a priori.

In our implementation of HDP modeling (see the flowchart in Fig. 1a), we randomly selected one million out of the six million records of asthma patients as input each time, and repeated the HDP modeling process 100 times, gathering a large collection of clusters. Some clusters had similar profiles, while others did not (partially due to the stochastic nature of HDP modeling). We measured the inter-cluster dissimilarity by Jensen-Shannon divergence and considering all the 567 disease dimensions, and applied HDBSCAN (Hierarchical Density-Based Spatial Clustering of Applications with Noise)^40–42^, discovering 22 stable subgroups of recurring clusters as well as their hierarchies. A subgroup was deemed to be stable if it harbored more than 50 cluster points. The number of cluster points enclosed in these 22 subgroups are 103, 103, 112, 93, 93, 125, 70, 87, 65, 104, 126, 67, 78, 79, 98, 107, 56, 74, 142, 68, 182, and 100, respectively. In particular, if we only look at the eleven subgroups that can be replicated in other cohort settings, their number of belonging cluster points are 103, 93, 125, 70, 126, 78, 79, 98, 56, 74, and 100, respectively. We understood that the threshold number of cluster points for claiming a stable subgroup was an important hyperparameter. Therefore, at the very beginning, we tested different numbers, for example, 25, 50, and 100, yielding 29, 22, and two subgroup partitions, respectively. We found that 50 was the optimal threshold number, leading to the 22 subgroups that suggested a reasonable nosology, as judged by physicians in our team. While the comorbidity patterns seen in the 29 subgroups (if the threshold number of cluster points for claiming a stable subgroup is set to be 25) appeared to be scattered and trivial, and the comorbidity patterns seen in the two subgroups (if the threshold number of cluster points for claiming a stable subgroup is set to be 100) would be too coarse. We additionally justified the hyperparameter selection of 50 cluster points using the elbow method. In detail, we tried different threshold numbers of cluster points for claiming a stable subgroup, and compute their mean stability scores^41^ of all the resulting subgroups after specifying a threshold number. We then plot these mean stability scores against the threshold numbers of cluster points (see Supplementary Fig. 1a). The location in the plot at which the increase of the mean stability scores switches from fast to slow (the elbow location) is regarded as the indicator of the optimal threshold number. In this work, the optimal number is 50 (indicated by a dashed line in the plot).

The occurring frequency of a given disease in the subgroup can be precisely quantified by the median value as well as the minimum, the first quartile, the third quartile, and maximum of the frequency values of the disease in the enclosed clusters collectively (see Supplementary Data 1 for the subgroup profiles in detail). Just for visualization purposes, we show the *t*-SNE two-dimensional projection of the identified asthma subgroups in Supplementary Fig. 1b.

Furthermore, we examined the sensitivity of modeling results towards four different cohort settings, including (i) the 3,152,519 individuals in the US MarketScan data who were aged between 15 and 70, but carried at least two asthma codes (as opposed to one asthma code used in the original configuration), (ii) the 3,401,250 individuals in the US MarketScan data who carried at least one asthma code, but were aged between 40 (as opposed to 15 used in the original configuration) and 70, (iii) the 3,687,965 individuals in the US MarketScan data who not only were aged between 15 and 70 and carried at least one asthma code, but also had at least one type of asthma drug prescriptions (the asthma drug prescriptions that are documented in the database include antibody inhibitor, inhaled corticosteroids, inhaled steroid combinations with long-acting beta agonists, leukotriene modifiers, mast cell stabilizers, methylxanthines, short-acting inhaled beta-2 agonists, and systemic corticosteroids), and (iv) the 66,448 individuals enrolled in UK Biobank who carried at least one asthma code and were aged between 39 and 72. Note that UK Biobank, different from MarketScan’s administrative claims-based database, is a national health registry dataset and more skewed towards an older and white-ancestry population (see Table 1 and Supplementary Table 4 for comparison details). By repeating the exact same procedure as described above (see the flowchart in Fig. 1a), we successfully replicated 21, 20, 22, and eleven subgroups, respectively, out of the original 22 (see Supplementary Data 2–5 for the subgroup profiles). In order to assess whether any of the subgroups generated based on the cohorts for sensitivity analyses can be claimed as successful replications of the subgroups discovered based on the discovery cohort, we computed their Pearson’s correlations based on the median frequency profiles of comorbid diseases in the respective subgroups. We only claim a successful replication if the respective correlation is determined to be significant. The common set of the successful replications of the discovered subgroups using all four different cohort settings comprised eleven subgroups (see Supplementary Table 2), and we specifically termed them “asthma subgroups”. For easier reference, each asthma subgroup is named after the broader category to which several most frequently-occurring diseases belonged, although it is the distribution of 567 disease groups that completely define the subgroup (see Supplementary Fig. 1c). As a summary, we analyzed independent large asthma cohorts and found that the identified asthma subgroups were largely consistent. Using the two largest datasets, US Marketscan and UK Biobank, we identified eleven stable topics/subgroups. Note that we aimed at arriving a not necessarily exhaustive but necessarily stable set of topics/subgroups across at least two datasets.

In next, considering that databases, such as US MarketScan used here, contain diagnosis information about individuals in different abundance and for different durations, we wanted to examine the extent to which the discovered subgroups were proxies of diagnosis code counts or observation times, or in other words, to find out whether the eleven subgroups end up with similar diagnosis counts and observation times. Therefore, we reported the summary statistics of individuals’ diagnosis code counts in each of the eleven subgroups and all of them combined based on US MarketScan data (see Supplementary Table 11 for their minimum, the first quartile, the median, the mean, the third quartile, and maximum values). Given the individuals’ diagnosis code counts of any two subgroups or all the eleven subgroups combined, we can assess their distribution similarity by estimating the overlapping area of their kernel density estimations^131^. In total, we examined 66 comparisons of subgroup pairs by exhausting all the possible pair combinations of the eleven asthma subgroups and all of them combined, i.e., $\left(12 \atop 2\right)=66$ (see Supplementary Table 12). The distribution similarity metric is equal to 1 for two identical distributions and 0 for two completely dissimilar ones. We found that the median and mean similarity values were 0.731 and 0.714, respectively. In addition, we compared enrollment patterns (visibility of patients in claims) of patients in all putative asthma subgroups and them combined. We computed (a) the total enrollment time (the duration when an individual stays enrolled) and (b) total diagnosis recording time (the duration from the time of the individual’s first diagnosis record to the time of the last diagnosis record). Supplementary Table 13 summarizes the summary statistics of these duration values in a subgroup-specific manner, and Supplementary Table 14 reports their distribution similarity values for any two subgroup (or all the eleven subgroups combined) pairs out of 66 possible comparisons. The results show that distributions of observation times across subgroups (or combined subgroups) are very similar: (a) for the enrollment durations, the median and mean similarity values are 0.820 and 0.814, respectively; (b) for the recording durations, the median and mean similarity values are even higher, 0.835 and 0.840, respectively. Altogether, these large similarity values suggest that there exists no systemic difference between subgroups and between single subgroups and combined subgroups in terms of diagnosis code counts or observation times. In rare cases, “GI” and “Lymphoma” subgroups have relatively low similarity value (0.4119) in the distribution comparison of their code counts, but still the similarity between the distributions of their enrollment or recording durations is high (~0.7).

Lastly, in order to check whether subgroup assignment to individuals solely depended on the single, most frequently occurring disease or not, we computed two types of assignment fraction values based on the diagnosis records of asthma patients. Taking the “Psychiatric” subgroup (the most frequently occurring disease is “Depression”) as an example, we computed (i) the fraction of patients who are in the “Psychiatric” subgroup indeed carry the “Depression” code, and (ii) the fraction of patients who carry the “Depression” code are eventually assigned to the “Psychiatric” subgroup. As a result, for the “Psychiatric” subgroup, the fraction i is 0.902, indicating a large majority of patients in the subgroup do carry the top code (interestingly, the remaining 10% of patients do not have to carry the top code in order to be assigned to the subgroup). The fraction ii is as low as 0.208, suggesting that having the top code alone is far from guaranteeing one to be assigned to the respective subgroup and other codes as well as their occurring frequencies play a role in such subgroup assignment process. Similar phenomena can also be observed in the other ten subgroups (Supplementary Table 15).

### Asthma subgroup assignment

After stable asthma subgroups are identified, the next task is to find an appropriate subgroup label for each individual that can best describe her/his comorbidity pattern, and to do this assignment for both asthmatic and non-asthmatic individuals. In fact, we purposely intended to use the subgroups discovered in asthma patients to classify non-asthma patients as well, so that we could compare asthma and non-asthma individuals who fell into the same subgroup (or in other words, shared the same comorbidity pattern), for example, in genome-wide association analysis.

From the perspective of matrix factorization, the statistical inference process described in Methods above can be expressed as finding a low-dimensional representation for the record-diagnosis (document-word) co-occurrence matrix of ${W}_{d,n}$ by decomposing it into the matrix of subgroup (topic) proportions ${\varTheta }_{d,k}$ and the matrix of subgroups (topics) ${\varPhi }_{k,n}$ (see Supplementary Fig. 4b, and its notations are the same as those used in Supplementary Fig. 4a). Given ${W}_{d,n}$ (observed) and ${\varPhi }_{k,n}$ (identified by HDP modeling), we can estimate ${\varTheta }_{d,k}$ by minimizing the least-square errors between the left- and right-hand sides of the equation. Finally, we labeled the individual $d$ with the subgroup of which the respective proportion value was the highest among $\left\{{\varTheta }_{d,1},\cdots,{\varTheta }_{d,k}\right\}$. In other words, given the distribution of diagnosis counts shown in an individual’s record, we tried to express it as a linear combination of the distributions of diagnosis counts as defined in the asthma subgroups, and then suggested that the subgroup with the largest assigned coefficient could represent the individual’s record best. It is worth emphasizing that the subgroup assignment accounts for (*i*) not a few dominant diseases in one’s diagnosis record but the complete collection of diseases therein, and (*ii*) not just the diseases’ presence but their frequencies of appearance in records.

This subgroup assignment process was applied to all the participating cohorts prior to the analyses of genome-wide associations, replications, differential gene expression, and phenotypic associations (see Supplementary Fig. 5 for the allocations of these cohorts to the asthma subgroups).

### UK Biobank (UKB) database and GWAS

The UKB database is a National Health Service registry database in the United Kingdom, including around 500,000 participants who were aged 40–69 years and recruited between 2006 and 2010^45^. This database was mainly used to find genotypes and phenotypes that appear to be significantly different between asthma cases and non-asthma controls in each of the eleven asthma subgroups that have been identified using the US MarketScan data. We selected the individuals who had diagnosis records plus genotype and/or phenotype data available. Diagnosis records were retrieved from both self-reports and medical assessments during regular visits, and this information was used in assigning participants to the identified asthma subgroups.

First of all, we checked whether there was some skew towards certain ancestry admixture for the eleven different asthma subgroups by examining the first (PC1) and the second (PC2) genetic principal components. We report the summary of PC1 and PC2 in the asthma case and non-asthma control pair in each of the eleven subgroups. Supplementary Table 10 summarizes the minimum, the first quartile, the median value, the mean value, the third quartile, and the maximum of PC1 and of PC2. Given the PC1 or PC2 values of two subgroups (either case or control), we can assess their distribution similarity by estimating the overlapping area of their kernel density estimations^131^. In total, we examine 231 comparisons of subgroup pairs by exhausting all the possible pair combinations of the 22 subgroups that include the eleven case subgroups and the eleven respective control subgroups, i.e., $\left(\genfrac{}{}{0ex}{}{22}{2}\right)=231$. The distribution similarity metric is equal to 1 for two identical distributions and 0 for two completely dissimilar ones. For PC1, the minimum, the first quartile, the median value, the mean value, the third quartile, and the maximum similarity values are as high as 0.874, 0.918, 0.947, 0.940, 0.962, and 0.980, respectively. For PC2, the minimum, the first quartile, the median value, the mean value, the third quartile, and the maximum similarity values are also very high, 0.848, 0.925, 0.943, 0.940, 0.958, and 0.985, respectively. These results suggest that none of the eleven asthma subgroups are enriched due to a particular ancestry admixture.

Within each subgroup, association analyses were performed to discover asthma-associated genetic variants and various phenotypes (see Methods “Associating with health-related phenotypes based on UKB phenotypic data”). In UKB, a total of around 96 million genetic variants, including genotyped and imputed variants, were eligible for genome-wide association analysis^45^. We chose the unrelated participants within the white British ancestry subset who were paired with high-quality genotype data and diagnosis records for the analysis, and the sample size was 305,098 (including 44,383 asthma cases who also had at least one comorbid disease). Furthermore, we imposed the following quality control thresholds: SNP call rate >0.95, minor allele frequency >0.01 and Hardy–Weinberg equilibrium *p* > 10^−6^.

We used a logistic-regression model to test statistical associations between additive SNP effects (i.e., 0, 1, 2 allele dosage coding) and asthma^46^, within the group of individuals with any comorbid diseases (the any-CDs group) or within each of the identified subgroups. It is worth noting that the asthma cases were always compared against the corresponding non-asthma controls that shared the same comorbidity pattern as defined in the respective subgroup. The covariates include sex, age of enrollment, and the first ten genetic principal components.

We considered an association to be suggestive and worthy of further investigation if its *p* < 10^−5^, and to be genome-wide significant if its *p* < 5 × 10^−8^^132^. The lead SNPs that met the suggestive threshold were subject to further statistical test on whether their effects were indeed significantly stronger than those found in the any-CDs group (see Methods “Stronger risk loci identification using a subsampling method”). Importantly, we identified 103 genome-wide significant loci in the any-CDs group and 20 in asthma subgroups (14 loci overlapped or 109 loci in union). To control the false discovery rate (FDR), we subjected all the GWAS results out of the twelve GWASs (in eleven subgroups and in a general asthma population) to multiple testing corrections using the Benjamini–Hochberg procedure. All the genome-wide significant loci we reported in Supplementary Data 7 were still significant after multiple testing corrections, with all FDR values <0.001. Out of these loci identified in any-CDs group and in asthma subgroups, 49 and 10 loci, respectively, were reproducible in a follow-up multi-ancestry meta-analysis across two different ethnicity subsets of UK Biobank, BioVU, and BBJ. In particular, there were six loci that conferred asthma risk to one asthma subgroup only but not to others (see Methods for technical details, Supplementary Table 1 for summary statistics, and Supplementary Fig. 2 for selected GWAS plots). We also checked whether our identified risk loci were in linkage disequilibrium (LD) with any previously reported loci in the NHGRI-EBI GWAS catalog database^47^, and only claimed a novel finding if the LD measured by *r*^2^ was smaller than 0.05 (based on 1000 Genomes reference panel that is specific to British in England and Scotland). As a result, 18 out of the 109 identified loci were novel, including five subgroup-specific ones (see Supplementary Data 7).

In addition, we assessed the heterogeneity of per-locus effect sizes, i.e., ln(OR) estimates, across all subgroups by applying Cochran’s *Q* test^48^. As a result, nine out of the 109 identified loci showed evidence of significant heterogeneity in effect sizes across asthma subgroups (see Supplementary Data 6).

### Replicating genome-wide significant associations in multi-ancestry meta-analysis

To replicate the genome-wide significant associations discovered using the white British subset in UKB, we leveraged another four independent cohorts. Two were taken from other ethnic subsets in UKB, and specifically, we selected the unrelated individuals with high-quality genotyping: (i) 22,600 individuals of white Irish and any other white background (including 3186 asthma cases who also had at least one comorbid disease), and (ii) 6833 individuals of African, Caribbean and any other black background (including 998 asthma cases who also had at least one comorbid disease).

As for the third cohort, we introduced another database—BioVU, a de-identified DNA databank from the Vanderbilt University Medical Center^54^. DNA samples were collected from routine clinical testing that would otherwise be discarded, and were linked to phenotypic data derived from electronic medical records (EMR) system. The clinical information in EMRs is updated every 1–3 months. The DNA samples underwent genome-wide genotyping with arrays including the Multi-Ethnic Global array, and then genotypes were imputed according to the HRC reference panel^133^ using the Michigan imputation server^134^. For replication analysis, we selected 16,060 individuals of European descent (determined by principal component analysis), which included 1,668 asthma cases with at least one comorbid disease.

The fourth cohort was the East Asian ethnic group from BBJ project, which was launched in 2003 to implement personalized medicine and is being conducted in three 5-year periods. The BBJ is a patient-based registry of around 200,000 participants who are of East Asian descent and diagnosed with any of 47 target common diseases. These target diseases, covering 15 broad categories, were selected owing to their clinical importance related to morbidity or mortality in Japan. Through the cooperation of 12 medical institutes, consisting of 66 hospitals, clinical information was collected and DNA samples were sequenced for genomic analyses^58^. Details about genotyping and imputation can be found in reference^56^. Previous analyses and comparisons against other Japanese databases using BBJ revealed largely consistent trends in common clinical variables, indicating that BBJ can represent the general patient population in Japan^57^. For the replication analysis, we selected a total of 194,413 individuals who had both diagnostic records and high-quality genotyping data, in which there were 3,368 asthma patients with at least one comorbid disease.

Based on these four independent cohorts, we performed a multi-ancestry meta-analysis in the following three steps. First, as described in Methods “Asthma subgroup assignment”, we assigned asthma cases and non-asthma controls to the identified asthma comorbidity subgroups (see Supplementary Fig. 5 for the numbers of allocated cases and controls). Second, focusing one cohort at a time, we conducted a multivariate logistic-regression analysis using sex, age, and the first ten genetic principal components as covariates, except for BioVU data, in which the covariates included sex, age, the first three genetic principal components of ancestry, and genotyping array type/batch. In the case of BBJ, several target SNPs were neither genotyped nor imputed, we used the SNPs in the highest LD with respect to the target SNPs if available (LD measured by *r*^2^, according to 1000 Genomes East Asian reference panel, March 2012 release; see Supplementary Fig. 3 for details). The final step was to merge these individual summary statistics, and we performed a meta-analysis by assuming a fixed effects model with inverse variance weighting^49–51^. The merged effect size can be calculated as the weighted average of all individual effect sizes:

1 $${\hat{\beta }}_{F}=\frac{{w}_{1}{\hat{\beta }}_{1}+{w}_{2}{\hat{\beta }}_{2}+{w}_{3}{\hat{\beta }}_{3}+{w}_{4}{\hat{\beta }}_{4}}{{w}_{1}+{w}_{2}+{w}_{3}+{w}_{4}}$$

and the merged variance is

2 $${var}\left({\hat{\beta }}_{F}\right)=\frac{1}{{w}_{1}+{w}_{2}+{w}_{3}+{w}_{4}}$$

where ${\hat{\beta }}_{1}$, ${\hat{\beta }}_{2}$, ${\hat{\beta }}_{3}$, and ${\hat{\beta }}_{4}$ are effect sizes (i.e., logarithm of odds ratios) using the white Irish and black subsets of UKB, the European-descent subset of BioVU, and the East Asian group of BBJ, respectively; ${w}_{1}$, ${w}_{2}$, ${w}_{3}$, and ${w}_{4}$ are their associated weights (i.e., the reciprocal of the respective squared standard errors)^52,53^. Since an association replicates only if the sign of effect sizes matches between the discovery and replication analyses, we used a one-sided *p* value to test replication, with an expected association direction based on the discovery analysis^135,136^. Out of the 128 discovered associations (involving 109 independent loci), 127 associations (involving 108 loci) were eligible for replication, and the only one exception was due to the small sample size (i.e., none of the four cohorts had more than 100 asthma cases allocated to the subgroup). After controlling the FDR using Benjamini–Hochberg procedure^137,138^, we successfully replicated 61 associations (involving 52 loci, FDR < 0.10). The detailed results are summarized in Supplementary Data 7. Among the 61 associations that were successfully replicated at an overall meta-analysis FDR of 0.1, there are ten associations that have FDR values right around 0.05 (from 0.05 to 0.06) and another ten associations that have FDR values greater than 0.06. By carefully examining these 20 replication results for which FDR values fall between 0.05 and 0.1, we find different degrees of inconsistency in the direction of SNP effects found in the four replication cohorts: Compared to the effect direction found in the discovery cohort (UKB British white group), there are one, three, six, and one replication showing effects of opposite directions in UKB Irish and other white groups; UKB African, Caribbean, and other black group; BioVU European-descent group; and BBJ (only nine out of 20 associations have enough samples for replication attempts in the first place), respectively. Such inconsistency in effect directions would be greater for the other 66 associations that were not replicated (FDR > 0.1), particularly in the UKB black and BioVU groups which show 34 and 26 cases with inconsistent directions, respectively.

### Differential gene expression analysis

We wanted to test for differential expression of the genes to which the six subgroup-specific SNPs were mapped. Thus, we introduced an independent dataset, containing transcriptome profiles of bronchial epithelial cells (BECs) in 42 asthma cases and 28 non-asthma controls enrolled in the University of Chicago hospitals^19^. The involved cDNA libraries were constructed using the TruSeq RNA Sample Preparation v2 Guide (Illumina) and run on the Illumina HiSEquation 2000 platform. Reads were mapped to the transcriptome using BWA (Burrows-Wheeler Aligner)^139^. BEDTools was used to determine the sequences that would overlap with protein-coding regions^140^. The mapped reads per individual ranged from 10,100,000 to 51,150,000, with median value to be 19,210,000. The reads were adjusted for gene length and variation in sample read depth, and then normalized using upper quartile normalization.

Using diagnosis history information, we first assigned the 42 asthma patients to the five subgroups that the six SNPs (meeting genome-wide significance threshold) related to. Only two subgroups involving three SNPs had five or more individuals: subgroup 5 “Musculoskeletal” had five cases and subgroup 3 “GI” had seven cases. The three SNPs, including *rs11144271*, *rs113757163*, and *rs2249851*, closest to genes *OSTF1*, *COX10*, and *FAM129B*, respectively, which were subject to differential gene expression analysis. Two types of control groups were compared against: (i) the 28 non-asthmatic individuals, and (ii) the remaining asthma cases that were assigned to the subgroups other than the one to be tested.

In this analysis, we first normalized the raw gene transcript counts by size factors to account for sequencing depth differences, estimated gene-wise dispersions, and then modeled the counts using a generalized linear model of the negative binomial family^60^. The confounding factors considered in the model included age, sex, and ethnicity. The significance of the test associations between gene counts and asthma subgroups were determined using the two-sided Wald test. In subgroup 11 (joint disorder), *OSTF1* was significantly lower expressed, while *COX10* was higher expressed, if compared with the respective expression levels in controls (i) and (ii). In subgroup 3 “GI,” the expression of *FAM129B* was significantly higher than those in the controls (Fig. 3c).

### Associating with health-related phenotypes based on UKB phenotypic data

To examine heterogeneity in phenotypic associations across the asthma subgroups, we made use of the phenotypic data in the UKB resource^45^ by focusing on a collection of 140 phenotypes that measured ten general categories related to health, including spirometry, blood count, blood biochemistry, urine biochemistry, early life factors, anthropometry, addictions, diet, physical activity, and local environment. Spirometry, in particular, includes pulmonary function measures on FVC, FEV_1_, FEV_1_/FVC, and PEF. After computing their respective predicted values using the prediction equations for Caucasian male and female adults developed from the third US National Health and Nutrition Examination Survey^141^, we further derived their percentage predicted values by normalizing the measured against the predicted values. Finally, min-max normalization was applied to all the phenotypic measures, so that their values all varied from 0 to 1 and the slope estimates of their associations could be compared to each other.

This analysis was based on the same samples as used in GWAS discovery, i.e., the unrelated individuals who had diagnosis records available and were in the white British ethnic group of UKB, including about 44,383 asthma cases and 260,715 non-asthma controls. The analysis consists of four steps:

1. Find appropriate subgroup assignment for all the samples, with or without asthma.
2. In a given subgroup $i$, pick a phenotypic measure and associate it with asthma diagnosis (yes or no) in a multivariate logistic-regression analysis using sex, age of enrollment, and the first ten genetic principal components as covariates (height is also included, if the phenotypic measure relates to spirometry). The resulting slope estimate of the phenotype (${\beta }_{i}$) characterizes how asthma likelihood associates with the phenotype: a positive (or negative) value indicates a positive (or negative) association; greater the absolute value is, stronger the association is.
3. Repeat step 2 for all the 140 phenotypic measures and for all the eleven asthma subgroups as well as the any-CDs group. The false discovery rate was controlled via Benjamini–Hochberg procedure^137,138^. Particularly, the slope (${\beta }_{0}$) from the any-CDs group would serve as a benchmark value to be used in the next step. The detailed results generated in this step can be found in Supplementary Data 11 (the raw slope estimates before benchmarking against any-CDs group).
4. Estimate the deviation of ${\beta }_{i}$ from ${\beta }_{0}$ and test its statistical significance, allowing for a quantitative assessment of heterogeneity in ${\beta }_{i}$ across different subgroups by comparing to the common benchmark ${\beta }_{0}$. To this end, we implemented a multivariate adaptive shrinkage (mash) method, which took the ${\beta }_{i}$ estimates as well as their standard errors as inputs and adopted an empirical Bayes procedure^63^. Out of the 140 phenotypes, 44 showed significant heterogeneity in ${\beta }_{i}$ across asthma subgroups. The final results are summarized in Fig. 4, Supplementary Fig. 7, and Supplementary Data 12 (the estimates of the slope differences after benchmarking against any-CDs group).

### Stronger risk loci identification using a subsampling method

Here, we asked among the asthma associations found in the subgroups that had passed the suggestive threshold (*p* < 10^−5^), how many of them were indeed significantly stronger than those found in the any-CDs group. To make a fair comparison of GWAS statistics, however, we needed to equate their statistical detection powers first.

As statistical power is largely influenced by sample size, detecting an association within an asthma subgroup, which is a subset of the undivided general population, is relatively less powered. This can be demonstrated using the mathematical formula for $Z$ score, written as below:

3 $$Z=\frac{\hat{\beta }}{{\hat{\sigma }}_{\hat{\beta }}}=\frac{\hat{\beta }}{\hat{\,\sigma }/\sqrt{n}}$$

where $\hat{\beta }$ is a SNP effect size (i.e., the logarithm of the odds ratio), ${\hat{\sigma }}_{\hat{\beta }}$ is the standard error, and $\hat{\sigma }$ is the sample standard deviation. $n$ denotes (effective) sample size and can be approximated via $1/\left(\frac{1}{{n}_{{case}}}+\frac{1}{{n}_{{ctrl}}}\right)$, where ${n}_{{case}}$ and ${n}_{{ctrl}}$ are the numbers of asthma cases and non-asthma controls, respectively. Here, $Z$ score is preferred over *p* value, in order to encode not only the significance level (reflected by the magnitude of $Z$ score) but also the direction of SNP effect (reflected by the sign). Detecting a SNP-asthma association, although its actual $\hat{\beta }$ and $\hat{\sigma }$ remain unchanged, would yield different $Z$ scores if cohorts with different sample sizes $n$ were used. Therefore, for an association in the any-CDs group (based on the general population who may have any comorbid diseases, ${n}_{{case}}^{g}$ cases and ${n}_{{ctrl}}^{g}$ controls), we should re-estimate what its ${Z}^{g}$ score would have been if it had been based on the cases and controls of the same sizes (${n}_{{case}}^{s}$ and ${n}_{{ctrl}}^{s}$, respectively) as a subgroup had (we called it the projected ${Z}^{g}$ here), in order to make a fair comparison against the subgroup-based ${Z}^{s}$ score.

In this analysis, we inferred the projected ${Z}^{g}$ empirically using a stratified subsampling algorithm. From each subgroup of cases (or controls), we randomly drew a number of samples, and this number was proportional to the original size of the cases’ (or controls’) subgroup; the total number of cases (or controls) we drew from all the subgroups should equal to ${n}_{{case}}^{s}$ (or ${n}_{{ctrl}}^{s}$). In other words, the original ${n}_{{case}}^{g}$ and ${n}_{{ctrl}}^{g}$ seen in the any-CDs group were shrunk to ${n}_{{case}}^{s}$ and ${n}_{{ctrl}}^{s}$, respectively, with their respective compositions of subgroups proportionally unchanged. Then, based on the newly generated subsamples, we performed the logistic-regression analysis as described in Methods “UK Biobank (UKB) database and GWAS” to compute the empirical estimates of the projected ${Z}^{g}$. But this was just one empirical estimate based on one possible set of subsamples. In practice, we repeated this subsampling process followed by the regression analysis for 20,000 times, thus generating a collection of 20,000 projected ${Z}^{g}$ scores.

Finally, we can test the null hypothesis: the subgroup-based ${Z}^{s}$ score followed the same distribution as defined by the projected scores collected above. Assuming this hypothesis was true, we computed an empirical two-tailed *p* value, which suggested the probability of getting the test statistic at least as extreme as ${Z}^{s}$. In this manner, we computed *p* values for all the possible associations between the lead SNPs of interest and asthma subgroups. Then we controlled the FDR and adjusted the *p* values using Benjamini–Hochberg procedure^137,138^. If an FDR was <0.05, then we would reject the null hypothesis about the respective association, declaring that in fact the association had an extremer-than-expected $Z$ score, and was significantly stronger in the subgroup and in the any-CDs group. Altogether, there were 182 associations of this kind (involving 182 loci) identified (see Supplementary Data 8 for a detailed summary).

### Identifying genomic regions that share influences on asthma

First, the 22 autosomes were divided into 1703, approximately independent regions based on patterns of LD that were derived from the European population in 1000 Genomes reference panel^142^, and on average each region contained 3054 SNPs. We wanted to know whether there existed genomic regions that shared asthma-associated influences (i) between asthma subgroups and the any-CDs group, and (ii) between the subgroups. For this purpose, by comparing GWAS summary statistics, we implemented an established hierarchical Bayesian model to estimate the probability that a genomic region contained at least one variant that influenced asthma susceptibility in (i) or (ii)^143^. More specifically, we performed a scan for genomic regions, computed a regional Bayes factor that measured the support for an association in a given genomic region, and inferred the posterior probability by maximizing a log-likelihood function. At a threshold of the posterior probability greater than 0.9 (i.e., at an FDR of 0.10), 73 unique genomic regions were identified for the pairs in (i) (Supplementary Data 9), and 21 unique genomic regions for the pairs in (ii) (Supplementary Data 10 and see Supplementary Fig. 6 for most conserved genomic regions that were shared by the any-CDs group and at least four subgroups).

### Pathway enrichment analysis based on GWAS summary statistics

Here, we aimed to find out unique biological pathways that were enriched in an asthma subgroup-specific manner. In each subgroup, we selected the lead SNPs that surpassed the suggestive threshold (*p* < 10^−5^), and mapped these SNPs to genes using positional, *eQTL*, and chromatin interaction information. In order to find possible overrepresentation of biological pathways and agreement with GWAS catalog, these mapped genes were tested against “background” gene sets obtained from MSigDB (i.e., hallmark gene sets, positional gene sets, curated gene sets, motif gene sets, computational gene sets, GO gene sets, oncogenic signatures, and immunologic signatures), WikiPathways (19,283 protein-coding genes), and GWAS catalog genes. Hypergeometric test was used and the resulting *p* values per category (i.e., canonical pathways, GO biological processes, and GWAS catalog, separately) were further adjusted via Benjamini–Hochberg correction^144^. Finally, we reported significant findings (Benjamini–Hochberg adjusted *p* value <0.05) in Supplementary Table 3.

### Reporting summary

Further information on research design is available in the Nature Research Reporting Summary linked to this article.

## Supplementary information

Supplementary Information

Description of Additional Supplementary Files

Reporting Summary

Supplementary Data 1

Supplementary Data 2

Supplementary Data 3

Supplementary Data 4

Supplementary Data 5

Supplementary Data 6

Supplementary Data 7

Supplementary Data 8

Supplementary Data 9

Supplementary Data 10

Supplementary Data 11

Supplementary Data 12

## Data Availability

The license of MarketScan databases is available to purchase by Federal, nonprofit, academic, pharmaceutical, and other researchers. Access to the data is contingent on completing a data use agreement and purchasing the needed license. More information about licensing the MarketScan databases can be found at https://www.ibm.com/us-en/marketplace/marketscan-research-databases. The phenotypic and genetic datasets of UK Biobank used in this study are available via the UK Biobank data access process, and the application for data access includes six steps and takes 21 weeks on average for the year 2020 (see https://www.ukbiobank.ac.uk/enable-your-research/apply-for-access); detailed information about the data can be found at http://www.ukbiobank.ac.uk/scientists-3/genetic-data/ and http://biobank.ctsu.ox.ac.uk/crystal/label.cgi?id=100314. Access to the phenotypic and genetic datasets of BioVU can be requested after a study proposal is received, approved by the BioVU Review Committee and a user agreement is signed. More information can be found at https://victr.vumc.org/how-to-use-biovu/. The transcriptome data of BECs were deposited in the GEO (https://www.ncbi.nlm.nih.gov/geo/) under accession GSE201955. The availability of the phenotypic and genetic datasets of BBJ is described at https://biobankjp.org/english/index.html, and more information can be found at https://humandbs.biosciencedbc.jp/en/hum0014-v21. The other data supporting the findings from this study are available within the manuscript and its supplementary information. Source data are provided with this paper.

## Source data

Source Data

## Code availability

The Hierarchical Dirichlet Process modeling was done by using a publicly available Github repository at https://github.com/blei-lab/hdp^35,36^. Genome-wide association analyses were performed using PLINK software version 2.0, which was downloaded at https://www.cog-genomics.org/plink/2.0/ and the name of software zip file was plink2_linux_x86_64_20180107.zip. Statistical analyses and plotting were done using RStudio version 1.2.5033.
