# Supplementary material for: Discerning asthma endotypes through comorbidity mapping
Source: Nat Commun. 2022 Nov 7;13:6712. doi: 10.1038/s41467-022-33628-8 (PMC9640644; doi:10.1038/s41467-022-33628-8)
Supplement: Supplementary file 1 — Supplementary Information [file 41467_2022_33628_MOESM1_ESM.pdf]

Supplementary Materials for

Discerning asthma endotypes through comorbidity mapping

Gengjie Jia, Xue Zhong, Hae Kyung Im, Nathan Schoettler, Milton Pividori, D. Kyle Hogarth, Anne I. Sperling, Steven R. White, Edward T. Naureckas, Christopher S. Lyttle, Chikashi Terao, Yoichiro Kamatani, Masato Akiyama, Koichi Matsuda, Michiaki Kubo, Nancy J. Cox, Carole Ober*, Andrey Rzhetsky*, Julian Solway* (*co-senior authors)

Correspondence to: jsolway@medicine.bsd.uchicago.edu, arzhetsky@uchicago.edu, c-ober@bsd.uchicago.edu

This PDF file includes:

Supplementary Notes
Supplementary Tables 1-15
Supplementary Figs. 1-7
Captions for Supplementary Data 1-12

Supplementary Notes

1. Searching for genomic loci with stronger effects in asthma subgroups than in the any-CDs group

We were also interested in loci that met only a suggestive significance threshold ($p < 10^{-5}$) but not a genome-wide significance threshold ($p < 5\times10^{-8}$), yet still appeared to exhibit comparably stronger effects in individual asthma subgroups than in the any-CDs group. We proposed to use a subsampling method to enable such comparisons (see Methods for details). To demonstrate, we use the discovery process of rs71465403 (in *CPT1A*) as an example. Initial GWAS found that this SNP's association with asthma in subgroup 3 "GI" had a $\boldsymbol{Z}$ score of 4.93 ($p = 8.29\times10^{-7}$), compared to a $\boldsymbol{Z}$ score 3.36 ($p = 7.86\times10^{-4}$) in the any-CDs group. Noticing that the former's sample size (9,015 asthma cases and 49,635 unaffected controls) was much smaller than the latter's (44,383 cases and 260,715 controls), we therefore tried to match their sample sizes first, and then only looked at the association statistics based on the samples of comparable sizes. Specifically, we randomly drew the equivalent number of subsamples (*i.e.*, 9,015 cases and 49,635 controls) from the any-CDs group and re-estimated the association $\boldsymbol{Z}$ score for the group, repeating this process 20,000 times to generate a distribution of re-estimated $\boldsymbol{Z}$ scores. This distribution presents all the possible $\boldsymbol{Z}$ scores out of the association tests between rs71465403 and asthma in the any-CDs group, based on fewer samples (*i.e.*, 9,015 cases and 49,635 controls, the same sizes as the GI subgroup has). If the same distribution ought to be followed by the $\boldsymbol{Z}$ score of 4.93 that was observed in the GI-subgroup-specific asthma (null hypothesis), the

probability of getting a $\boldsymbol{Z}$ statistic at least as extreme was computed to be 4.89×10^{-5}. We then adjusted this probability value to 2.81×10^{-3} (< 0.05) after controlling the false discovery rate, and thus claimed that rs71465403 had a significantly stronger effect in the GI subgroup than that in the any-CDs group by rejecting the null hypothesis. In this way, we subjected all the SNPs that met the suggestive threshold ($p < 10^{-5}$) to the test, and in total, we identified 182 loci that showed comparably stronger effects in subgroups (see Supplementary Data 8 for a detailed summary).

On top part of the GWAS Manhattan plots of the five selected subgroups in Supplementary Fig. 2, we highlighted these significant loci in orange and annotated their mapped genes by positional proximity, *e.g.*, loci can be linked to intronic, intergenic, 3 prime UTR (UTR3), or other variants of genes. More specifically, in subgroup 3 "GI", we expected protein-coding genes, such as *SPATA17* (intronic), *NPFFR2* (intergenic), *RAD50* (intronic), *SNORA33* (intergenic), *SCAF8* (intronic), *IMMP2L* (intronic), *FOXP2* (intergenic), *ZMIZ1-AS1* (intronic), *CPT1A* (intronic), *VPS16* (intronic), and *NINL* (UTR3) to relate with the susceptibility to the GI-subgroup-specific type of asthma. These implicated genes showing stronger effects likely include many previously unsuspected players in the pathophysiology of asthma, possibly informing subgroup-specific asthma pathogeneses and eventually opening new avenues for therapeutic opportunities.

2. Detecting genomic regions with shared genetic influences in asthma subgroups and in the any-CDs group

To complete our query into the GWAS findings, we tested not only the genetic specificities between asthma subgroups and the any-CDs group, but also their genetic commonalities; in other words, whether genomic regions share asthma-associated influences between the two. For this purpose, we first divided the 22 autosomes into 1703 approximately independent regions based on patterns of linkage disequilibrium [1], and then for each region applied a hierarchical model to estimate the probability that a genomic locus contained at least one variant that influenced the both [2] (see Methods for details). A systematic scan through all the regions located 73 unique genomic regions that had shared influences between the subgroup(s) and the any-CDs group (see Supplementary Data 9 for the complete results). In Supplementary Fig. 2, these regions were colored in blue. We can see their great abundance in the relatively densely populated subgroups 3, 5 and 8, and their scarcity in sparsely populated subgroups 4 and 6 possibly due to limited detection powers. In the same way, we also investigated the genetic commonality between asthma subgroups, finding 21 unique genomic regions with shared influences (see Supplementary Data 10 for the complete results).

Supplementary Fig. 6 shows eight genomic regions in which genetic influences observed in the any-CDs group are shared with four or more different subgroups, and we consider them as conserved regions. In particular, two consecutive genomic regions, No. 655 and 656, covering SNP positions from 30 Mb to 32 Mb on chromosome 6, are among the most conserved regions (shared by five subgroups), and therein lie the Human Leukocytes Antigen (HLA) super-loci, which have been reported to associate with various asthma phenotypes [3] and with regulation of immune system, as well as some other fundamental cellular processes [4].

References:

1. Berisa, T. & Pickrell, J.K. Approximately independent linkage disequilibrium blocks in human populations. *Bioinformatics* **32**, 283-5 (2016).
2. Pickrell, J.K. *et al.* Detection and interpretation of shared genetic influences on 42 human traits. *Nat Genet* **48**, 709-17 (2016).
3. Shiina, T., Inoko, H. & Kulski, J.K. An update of the HLA genomic region, locus information and disease associations: 2004. *Tissue Antigens* **64**, 631-649 (2004).
4. Choo, S.Y. The HLA system: Genetics, immunology, clinical testing, and clinical implications. *Yonsei Medical Journal* **48**, 11-23 (2007).

Supplementary Table 1. Genome-wide significant associations with asthma in the general population with any comorbid diseases (the any-CDs group) or in their subgroups (see Methods "UK Biobank database and GWAS")

Asthma association in	Lead SNP [a]	Position [b]	Nearest gene	Allele [c]	AAF [d]	ln(OR) [e]	SE [f]	*p*-value
any CDs	rs1381928	1:8690933	*RERE*	A/G	0.361	-0.045	0.008	4.27×10^{-9}
any CDs	rs12123821	1:152179152	*HRNR*	C/T	0.048	0.160	0.016	5.07×10^{-23}
any CDs	rs61815704	1:152893891	*IVL*	C/G	0.024	0.148	0.023	1.22×10^{-10}
any CDs	rs7523907	1:167427247	*CD247*	T/C	0.454	-0.041	0.007	1.58×10^{-8}
any CDs	rs112819286*	1:168740269	*DPT*	A/G	0.177	-0.054	0.010	4.48×10^{-8}
any CDs	rs11590405	1:203080264	*ADORA1*	T/C	0.324	-0.053	0.008	1.36×10^{-11}
any CDs	rs10178845	2:8443803	*LINC00299*	G/A	0.294	-0.060	0.008	1.36×10^{-13}
any CDs	rs1007027	2:102731596	*IL1R1*	A/C	0.389	0.054	0.007	4.39×10^{-13}
[3.] GI	rs60227565	2:102892339	*IL1RL1*	G/A	0.130	-0.160	0.025	2.25×10^{-10}
any CDs	rs60227565	2:102892339	*IL1RL1*	G/A	0.130	-0.137	0.011	8.08×10^{-34}
[3.] GI	rs12470864	2:102926362	*IL1RL1*	G/A	0.388	0.093	0.017	1.78×10^{-8}
any CDs	rs12470864	2:102926362	*IL1RL1*	G/A	0.388	0.090	0.007	8.96×10^{-34}
any CDs	rs3771175	2:102960210	*IL1RL1*	T/A	0.137	-0.139	0.011	5.24×10^{-37}
any CDs	rs72837826*	2:111933001	*BCL2L11*	G/T	0.102	0.067	0.012	1.43×10^{-8}
[3.] GI	rs34290285	2:242698640	*D2HGDH*	G/A	0.255	-0.117	0.019	6.06×10^{-10}
[8.] Cardiovascular	rs34290285	2:242698640	*D2HGDH*	G/A	0.255	-0.134	0.019	6.37×10^{-12}
any CDs	rs34290285	2:242698640	*D2HGDH*	G/A	0.255	-0.103	0.008	1.13×10^{-33}
any CDs	rs35570272	3:33047662	*GLB1*	G/T	0.396	0.050	0.007	2.17×10^{-11}
any CDs	rs7626218	3:176852038	*TBL1XR1*	A/T	0.395	-0.047	0.007	2.27×10^{-10}
any CDs	rs6808893	3:188133439	*LPP*	T/C	0.488	0.051	0.007	5.71×10^{-12}
any CDs	rs45613035	4:123141070	*KIAA1109*	T/C	0.099	0.085	0.012	7.15×10^{-13}
any CDs	rs2069772	4:123373133	*IL2*	T/C	0.277	0.048	0.008	3.91×10^{-9}
any CDs	rs16903574	5:14610309	*FAM105A*	C/G	0.077	0.083	0.014	1.37×10^{-9}
any CDs	rs4594881	5:35846815	*IL7R*	G/T	0.342	-0.046	0.008	1.89×10^{-9}
any CDs	rs17513503	5:110146446	*SLC25A46*	C/G	0.074	0.089	0.014	6.90×10^{-11}
any CDs	rs10037959	5:110265787	*TSLP*	C/T	0.059	0.090	0.015	2.77×10^{-9}
[8.] Cardiovascular	rs1837253	5:110401872	*TSLP*	C/T	0.260	-0.125	0.019	9.22×10^{-11}
any CDs	rs1837253	5:110401872	*TSLP*	C/T	0.260	-0.106	0.008	6.96×10^{-36}
any CDs	rs114442993	5:110431897	*WDR36*	A/G	0.070	0.092	0.014	2.58×10^{-10}
any CDs	rs10491424	5:110453806	*WDR36*	T/C	0.356	-0.059	0.008	1.48×10^{-14}
any CDs	rs157577	5:131563571	*P4HA2*	C/G	0.279	0.045	0.008	2.14×10^{-8}
any CDs	rs1023518	5:131793772	*IRF1*	G/T	0.264	0.080	0.008	9.73×10^{-23}
any CDs	rs72797327	5:131843465	*IRF1*	A/G	0.259	-0.048	0.008	1.20×10^{-8}
any CDs	rs115008099	5:131991881	*IL13*	C/T	0.168	0.092	0.010	1.05×10^{-21}
any CDs	rs10455052	5:132034588	*KIF3A*	C/A	0.128	0.063	0.011	4.96×10^{-9}

any CDs	rs249677	5:141539339	*NDFIP1*	A/C	0.367	-0.046	0.008	1.98×10^{-9}
any CDs	rs6879838*	5:156966567	*ADAM19*	T/A	0.145	0.062	0.010	1.80×10^{-9}
any CDs	rs17312661	6:28300336	*ZSCAN31*	A/G	0.212	0.050	0.009	1.09×10^{-8}
any CDs	rs1233493*	6:29458241	*RPS17P1*	A/G	0.126	0.063	0.011	4.24×10^{-9}
[3.] GI	rs9271365	6:32586794	*HLA-DQA1*	T/G	0.431	0.129	0.016	3.62×10^{-15}
[5.]Musculoskeletal	rs9271365	6:32586794	*HLA-DQA1*	T/G	0.431	0.164	0.028	2.85×10^{-9}
[8.]Cardiovascular	rs9271365	6:32586794	*HLA-DQA1*	T/G	0.431	0.128	0.017	1.51×10^{-14}
any CDs	rs9271365	6:32586794	*HLA-DQA1*	T/G	0.431	0.138	0.007	7.12×10^{-80}
any CDs	rs1704996*	6:33182895	*ZNF70P1*	A/C	0.054	0.100	0.016	1.45×10^{-10}
any CDs	rs28607030	6:33716716	*IP6K3*	A/G	0.341	-0.048	0.008	4.76×10^{-10}
any CDs	rs9350929*	6:64175668	*EEF1B2P5*	G/A	0.447	-0.041	0.007	2.55×10^{-8}
any CDs	rs6899623	6:90986559	*BACH2*	A/G	0.354	-0.076	0.008	5.20×10^{-23}
any CDs	rs802731	6:128279429	*PTPRK*	C/G	0.271	0.051	0.008	3.52×10^{-10}
any CDs	rs2390314	7:20455978	*ITGB8*	T/A	0.071	0.083	0.014	3.32×10^{-9}
any CDs	rs13241235	7:20584837	*EEF1A1P27*	T/C	0.356	-0.042	0.008	2.95×10^{-8}
any CDs	rs13263709	8:81287175	*LOC100216346*	C/T	0.352	0.066	0.008	4.20×10^{-18}
any CDs	rs72693791	9:5816825	*ERMP1*	G/A	0.086	0.083	0.013	4.71×10^{-11}
any CDs	rs7047575	9:5841438	*ERMP1*	A/G	0.469	0.052	0.007	1.09×10^{-12}
[3.] GI	rs78728108	9:5966313	*KIAA2026*	T/C	0.046	0.234	0.037	2.01×10^{-10}
any CDs	rs78728108	9:5966313	*KIAA2026*	T/C	0.046	0.160	0.017	7.52×10^{-22}
any CDs	rs10975413	9:6049843	*RANBP6*	A/G	0.187	-0.097	0.010	4.59×10^{-24}
[8.]Cardiovascular	rs340928	9:6086913	*RANBP6*	G/A	0.045	0.204	0.037	4.38×10^{-8}
any CDs	rs340928	9:6086913	*RANBP6*	G/A	0.045	0.166	0.017	3.08×10^{-23}
[3.] GI	rs992969	9:6209697	*IL33*	G/A	0.252	0.151	0.018	9.77×10^{-17}
[8.]Cardiovascular	rs992969	9:6209697	*IL33*	G/A	0.252	0.129	0.019	6.29×10^{-12}
any CDs	rs992969	9:6209697	*IL33*	G/A	0.252	0.119	0.008	4.46×10^{-47}
any CDs	rs78757963	9:6282511	*IL33*	G/A	0.035	0.191	0.018	4.79×10^{-25}
any CDs	rs75636497	9:6545605	*GLDC*	C/G	0.117	-0.063	0.012	4.25×10^{-8}
any CDs	rs4742214	9:6548053	*GLDC*	C/G	0.366	0.047	0.008	4.80×10^{-10}
[5.]Musculoskeletal	rs11144271*	9:77785171	*OSTF1*	C/T	0.106	0.235	0.042	2.50×10^{-8}
any CDs	rs1888072*	9:92205987	*GADD45G*	C/T	0.492	-0.040	0.007	3.34×10^{-8}
any CDs	rs150707349*	9:101989706	*SEC61B*	A/G	0.031	-0.138	0.023	1.80×10^{-9}
[3.] GI	rs2249851*	9:130324154	*FAM129B*	A/G	0.260	0.108	0.018	3.30×10^{-9}
any CDs	rs11255507	10:8109615	*GATA3*	T/G	0.177	0.054	0.009	1.21×10^{-8}
any CDs	rs10905361	10:8455537	*LOC105376396*	C/T	0.456	0.040	0.007	2.99×10^{-8}
any CDs	rs12785018	10:8515348	*LOC105376397*	C/T	0.339	0.048	0.008	4.50×10^{-10}
[8.]Cardiovascular	rs2765400	10:8566517	*KRT8P37*	C/T	0.369	0.095	0.017	2.56×10^{-8}
any CDs	rs4749785	10:8603844	*KRT8P16*	C/T	0.270	0.048	0.008	3.05×10^{-9}

any CDs	rs2025758	10:8841669	*LOC105376400*	T/C	0.456	-0.056	0.007	1.83×10^{-14}
any CDs	rs6602347	10:8979851	*LOC105376400*	G/A	0.356	-0.043	0.008	1.97×10^{-8}
any CDs	rs118077070	10:9037669	*LOC105376400*	G/A	0.039	-0.112	0.020	1.37×10^{-8}
[3.] GI	rs186856025	10:9043457	*LOC105376400*	C/T	0.108	-0.176	0.028	1.95×10^{-10}
any CDs	rs186856025	10:9043457	*LOC105376400*	C/T	0.108	-0.128	0.012	1.17×10^{-25}
[8.]Cardiovascular	rs1612986	10:9064716	*LOC105376400*	T/C	0.187	0.115	0.021	3.51×10^{-8}
any CDs	rs1612986	10:9064716	*LOC105376400*	T/C	0.187	0.089	0.009	4.65×10^{-22}
any CDs	rs10490944	10:9143511	*LINC00709*	C/T	0.077	-0.119	0.014	4.11×10^{-17}
any CDs	rs17406680	10:9208204	*LINC00709*	G/C	0.052	0.121	0.016	1.33×10^{-14}
any CDs	rs75125788*	10:9255890	*LINC00709*	C/T	0.080	-0.078	0.014	1.12×10^{-8}
any CDs	rs35654771	10:9310831	*LINC00709*	C/A	0.156	0.057	0.010	1.56×10^{-8}
any CDs	rs174566	11:61592362	*FADS2*	A/G	0.350	-0.044	0.008	1.24×10^{-8}
any CDs	rs4945084	11:76121290	*THAP12*	C/G	0.332	0.042	0.008	3.12×10^{-8}
any CDs	rs1892958	11:76277902	*EMSY*	T/C	0.104	-0.068	0.012	3.43×10^{-8}
[3.] GI	rs7110818	11:76292575	*EMSY*	C/T	0.452	0.098	0.016	1.83×10^{-9}
any CDs	rs7110818	11:76292575	*EMSY*	C/T	0.452	0.092	0.007	1.41×10^{-36}
any CDs	rs55646091	11:76299431	*EMSY*	G/A	0.051	0.185	0.016	2.20×10^{-31}
any CDs	rs12365699	11:118743286	*CXCR5*	G/A	0.167	-0.059	0.010	3.50×10^{-9}
any CDs	rs705700	12:56389293	*RAB5B*	T/C	0.425	0.051	0.007	2.83×10^{-12}
any CDs	rs3024971	12:57493727	*STAT6*	T/G	0.107	-0.108	0.012	6.03×10^{-19}
any CDs	rs3001425	12:57509569	*STAT6*	C/T	0.404	0.065	0.007	3.08×10^{-18}
any CDs	rs17547610	12:57530341	*LRP1*	C/A	0.156	-0.065	0.010	1.75×10^{-10}
any CDs	rs3851611	12:71524042	*TSPAN8*	C/G	0.409	-0.045	0.007	9.77×10^{-10}
any CDs	rs7134784*	12:94582477	*PLXNC1*	C/T	0.150	-0.061	0.010	4.24×10^{-9}
any CDs	rs59186511	13:99986238	*UBAC2*	C/T	0.120	-0.074	0.011	7.34×10^{-11}
any CDs	rs10131490	14:68743307	*RAD51B*	A/G	0.279	0.052	0.008	1.48×10^{-10}
[6.] Lung	rs76225731*	15:25393616	*SNHG14*	A/G	0.038	0.626	0.114	3.66×10^{-8}
any CDs	rs34986765	15:61069201	*RORA*	T/C	0.130	-0.076	0.011	5.11×10^{-12}
any CDs	rs28617673	15:67371244	*SMAD3*	C/T	0.110	0.075	0.011	7.00×10^{-11}
[3.] GI	rs72743461	15:67441750	*SMAD3*	C/A	0.237	0.110	0.019	3.83×10^{-9}
[8.]Cardiovascular	rs72743461	15:67441750	*SMAD3*	C/A	0.237	0.116	0.019	1.33×10^{-9}
any CDs	rs72743461	15:67441750	*SMAD3*	C/A	0.237	0.104	0.008	3.59×10^{-35}
any CDs	rs10152595	15:67475488	*SMAD3*	C/G	0.248	-0.068	0.009	2.43×10^{-15}
any CDs	rs4842921*	15:84556623	*ADAMTSL3*	G/A	0.388	-0.042	0.007	1.83×10^{-8}
any CDs	rs35441874	16:11213021	*CLEC16A*	T/A	0.247	-0.084	0.009	1.14×10^{-22}
any CDs	rs117378200	16:11266124	*CLEC16A*	T/G	0.022	-0.150	0.027	1.70×10^{-8}
any CDs	rs4296278	16:11541685	*LOC400499*	G/A	0.169	0.054	0.010	2.27×10^{-8}
any CDs	rs3785356	16:27349168	*IL4R*	C/T	0.298	0.055	0.008	5.07×10^{-12}

any CDs	rs179771*	16:27417744	*IL21R*	G/C	0.488	0.040	0.007	3.90×10^{-8}
any CDs	rs112144981*	16:89418705	*ANKRD11*	C/G	0.157	-0.057	0.010	2.17×10^{-8}
[5.] Musculoskeletal	rs113757163*	17:13898421	*COX10*	G/A	0.022	0.498	0.083	1.58×10^{-9}
any CDs	rs146644295	17:37574592	*MED1*	G/C	0.022	0.163	0.024	9.20×10^{-12}
any CDs	rs145835664	17:37579383	*MED1*	G/A	0.151	-0.067	0.010	1.25×10^{-10}
any CDs	rs9903269	17:37742383	*CDK12*	T/A	0.178	0.078	0.009	9.64×10^{-17}
[3.] GI	rs1011082	17:38068514	*GSDMB*	T/C	0.480	0.101	0.016	4.50×10^{-10}
any CDs	rs1011082	17:38068514	*GSDMB*	T/C	0.480	0.097	0.007	2.43×10^{-40}
any CDs	rs62065216	17:38218773	*THRA*	G/A	0.425	0.051	0.007	5.11×10^{-12}
any CDs	rs4792811	17:43347336	*MAP3K14*	G/C	0.268	0.047	0.008	6.64×10^{-9}
any CDs	rs2074190	17:45811210	*TBX21*	A/G	0.257	-0.046	0.008	3.68×10^{-8}
any CDs	rs4141183	17:45858487	*OSBPL7*	C/T	0.131	0.062	0.011	6.44×10^{-9}
any CDs	rs16948048	17:47440466	*ZNF652*	A/G	0.369	0.056	0.007	6.70×10^{-14}
[8.] Cardiovascular	rs117710327	19:33726578	*SLC7A10*	C/A	0.067	-0.201	0.036	2.13×10^{-8}
any CDs	rs117710327	19:33726578	*SLC7A10*	C/A	0.067	-0.149	0.016	1.06×10^{-21}
any CDs	rs2834787	21:36502558	*RUNX1*	A/G	0.154	0.058	0.010	7.14×10^{-9}
[4.] Lymphoma	rs117262476*	21:47794898	*PCNT*	C/G	0.026	1.116	0.197	1.46×10^{-8}

[a] Lead SNPs are defined as SNPs which are significant (p-value < $5x10^{-8}$) and are independent from each other at $r^2 < 0.1$; in this column, SNPs never reported in any previously published asthma GWASs are marked with asterisks;

[b] Chromosome number: SNP position (Human Genome version 19);

[c] Reference/Alternative allele; **[d]** Alternative allele frequency (AAF);

[e] Natural logarithm of odds ratio, *i.e.*, ln(OR); **[f]** Standard error of ln(OR) estimate.

Supplementary Table 2. The top five most frequently-occurring diseases in all the subgroups identified based on different cohorts.

Asthma subgroups [a]	Discovery cohort (MarketScan: at least one asthma code, aged between 15 and 70) [b]	Sensitivity analysis 1 (MarketScan: at least two asthma codes, aged between 15 and 70) [c]	Sensitivity analysis 2 (MarketScan: at least one asthma code, aged between 40 and 70) [d]	Sensitivity analysis 3 (MarketScan: at least one asthma code and one asthma drug prescription, aged between 15 and 70) [e]	Sensitivity analysis 4 (UK Biobank: at least one asthma code) [f]
1. Diabetes	Type II Diabetes Mellitus: 58.1% + General Hypertension: 10.5% + Type I Diabetes Mellitus: 6.7% + Unspecified Hyperlipidemia: 4.3% + Unspecified Diabetes Mellitus: 2.2%	Type II Diabetes Mellitus: 57.7% + General Hypertension: 10.2% + Type I Diabetes Mellitus: 6.5% + Unspecified Hyperlipidemia: 3.9% + Unspecified Diabetes Mellitus: 2.1%	Type II Diabetes Mellitus: 60.4% + General Hypertension: 10.5% + Type I Diabetes Mellitus: 5.8% + Unspecified Hyperlipidemia: 4.0% + Unspecified Diabetes Mellitus: 2.3%	Type II Diabetes Mellitus: 59.0% + General Hypertension: 9.5% + Type I Diabetes Mellitus: 6.8% + Unspecified Hyperlipidemia: 3.7% + Unspecified Diabetes Mellitus: 2.1%	Type II Diabetes Mellitus: 44.3% + General Hypertension: 19.9% + Unspecified Diabetes Mellitus: 6.1% + Unspecified Lipid Metabolism Disorder: 4.7% + Type I Diabetes Mellitus: 4.3%
Pearson's correlation (*p*-value) [g]		0.999 (< 10^{-16})	0.999 (< 10^{-16})	0.999 (< 10^{-16})	0.958 (< 10^{-16})
2. Autoimmune	Rheumatoid Arthritis Related Conditions: 44.8% + Lupus Erythematosus: 9.8% + Non-Specific Joint Disorder: 3.2% + Non-Specific Arthritis: 3.2% + Sjogren's Syndrome: 2.9%	Rheumatoid Arthritis Related Conditions: 42.0% + Lupus Erythematosus: 9.6% + Sjogren's Syndrome: 2.8% + Non-Specific Joint Disorder: 2.7% + Non-Specific Arthritis: 2.6%	Rheumatoid Arthritis Related Conditions: 44.5% + Lupus Erythematosus: 8.9% + Non-Specific Joint Disorder: 3.1% + Sjogren's Syndrome: 2.9% + Non-Specific Arthritis: 2.5%	Rheumatoid Arthritis Related Conditions: 46.8% + Lupus Erythematosus: 9.6% + Non-Specific Joint Disorder: 3.5% + Non-Specific Arthritis: 2.9% + Sjogren's Syndrome: 2.7%	Rheumatoid Arthritis Related Conditions: 55.5% + Psoriasis Related Disorders: 12.4% + Osteoporosis: 2.1% + Osteoarthritis: 1.2% + Sjogren's Syndrome: 0.8%
Pearson's correlation (*p*-value)		0.999 (< 10^{-16})	0.999 (< 10^{-16})	0.999 (< 10^{-16})	0.946 (< 10^{-16})
3. GI	Esophageal Disease: 16.9% + Non-Specific Gastrointestinal Disorder: 6.4% + Functional Digestive Disorder: 6.3% + Gastritis Duodenitis: 6.0% + Biliary Tract Disease: 5.4%	Esophageal Disease: 18.6% + Functional Digestive Disorder: 6.6% + Non-Specific Gastrointestinal Disorder: 6.5% + Gastritis Duodenitis: 6.1% + IBS: 4.9%	Esophageal Disease: 18.1% + Gastritis Duodenitis: 5.9% + Functional Digestive Disorder: 5.8% + Non-Specific Gastrointestinal Disorder: 5.8% + Hernia: 4.9%	Esophageal Disease: 18.3% + Functional Digestive Disorder: 6.5% + Non-Specific Gastrointestinal Disorder: 6.4% + Gastritis Duodenitis: 6.1% + Biliary Tract Disease: 5.4%	Hernia: 14.3% + Esophageal Disease: 10.6% + Non-Specific Gastrointestinal Disorder: 7.2% + Gastritis Duodenitis: 7.0% + Gastrointestinal Ulcer: 5.8%
Pearson's correlation (*p*-value)		0.998 (< 10^{-16})	0.995 (< 10^{-16})	0.999 (< 10^{-16})	0.767 (< 10^{-16})
4. Lymphoma	Non-Hodgkins Lymphoma: 15.0% + Unspecified White Blood Cell Disease: 5.7% + General Thrombocytopenia: 5.5% + Lymphoid Leukemia: 5.4% + Myeloid Leukemia: 3.9%	Non-Hodgkins Lymphoma: 19.0% + Myeloproliferative Disease: 8.6% + General Thrombocytopenia: 6.4% + Unspecified White Blood Cell Disease: 6.3% + Lymphoid Leukemia: 6.2%	Non-Hodgkins Lymphoma: 18.8% + Myeloproliferative Disease: 10.6% + Multiple Myeloma: 7.0% + Unspecified White Blood Cell Disease: 6.2% + Lymphoid Leukemia: 6.0%	Non-Hodgkins Lymphoma: 18.9% + Myeloproliferative Disease: 8.3% + Lymphoid Leukemia: 6.9% + Unspecified White Blood Cell Disease: 6.0% + General Thrombocytopenia: 4.8%	Non-Hodgkins Lymphoma: 43.6% + Unspecified White Blood Cell Disease: 4.2% + Hodgkins Disease: 3.0% + Septicemia: 2.0% + Other Infectious Diseases: 1.3%
Pearson's correlation (*p*-value)		0.946 (< 10^{-16})	0.896 (< 10^{-16})	0.953 (< 10^{-16})	0.832 (< 10^{-16})
5. Musculoskeletal	Non-Specific Joint Disorder: 31.6% + Muscle Ligament Disorder: 17.8% + Osteoarthritis: 15.2% + Non-Specific Acquired Musculoskeletal Abnormality: 7.0% + Non-Specific Pain: 6.7%	Non-Specific Joint Disorder: 29.6% + Osteoarthritis: 14.9% + Muscle Ligament Disorder: 11.3% + Non-Specific Acquired Musculoskeletal Abnormality: 6.7% + Non-Specific Pain: 6.7%	Non-Specific Joint Disorder: 16.4% + Muscle Ligament Disorder: 11.3% + Non-Specific Pain: 6.3% + Non-Specific Acquired Musculoskeletal Abnormality:	Non-Specific Joint Disorder: 31.7% + Muscle Ligament Disorder: 18.3% + Osteoarthritis: 16.2% + Non-Specific Acquired Musculoskeletal Abnormality:	Osteoarthritis: 18.6% + General Spondylosis Spine Disorder: 15.5% + Non-Specific Pain: 6.6% + Non-Specific Acquired Musculoskeletal

			4.6% + Synovium Tendon Bursa Disorder: 3.0%	7.0% + Non-Specific Pain: 6.5%	Abnormality: 6.1% + Muscle Ligament Disorder: 5.8%
Pearson's correlation (*p*-value)		0.990 (< 10^{-16})	0.937 (< 10^{-16})	0.999 (< 10^{-16})	0.547 (< 10^{-16})
6. Lung	Emphysema COPD: 52.3% + Non-Specific Pulmonary Disorder: 12.0% + Pneumonia: 8.2% + Acute Bronchitis: 3.6% + Non-Specific Cardiovascular Disease: 1.9%	Emphysema COPD: 52.2% + Non-Specific Pulmonary Disorder: 11.8% + Pneumonia: 8.0% + Acute Bronchitis: 3.6% + Non-Specific Cardiovascular Disease: 1.8%	Emphysema COPD: 55.7% + Non-Specific Pulmonary Disorder: 11.2% + Pneumonia: 7.5% + Acute Bronchitis: 3.6% + Substance Abuse: 1.8%	Emphysema COPD: 54.1% + Non-Specific Pulmonary Disorder: 11.3% + Pneumonia: 7.5% + Acute Bronchitis: 3.3% + Non-Specific Cardiovascular Disease: 1.8%	Emphysema COPD: 26.1% + Non-Specific Pulmonary Disorder: 20.2% + Pneumonia: 12.4% + Pleuritis: 5.2% + Upper Respiratory Inflammation: 3.7%
Pearson's correlation (*p*-value)		0.999 (< 10^{-16})	0.999 (< 10^{-16})	0.999 (< 10^{-16})	0.876 (< 10^{-16})
7. Heart	Cardiac Dysrhythmia: 19.4% + Atherosclerosis: 19.0% + Non-Specific Cardiovascular Disease: 10.0% + General Hypertension: 6.5% + Non-Rheumatic Heart Disease: 5.2%	Cardiac Dysrhythmia: 20.0% + Atherosclerosis: 18.1% + Non-Specific Cardiovascular Disease: 10.6% + General Hypertension: 6.6% + Non-Rheumatic Heart Disease: 5.4%	Atherosclerosis: 21.3% + Cardiac Dysrhythmia: 20.8% + Non-Specific Cardiovascular Disease: 10.8% + General Hypertension: 6.4% + Non-Specified Cardiac Ischemia: 5.6%	Cardiac Dysrhythmia: 19.9% + Atherosclerosis: 19.4% + Non-Specific Cardiovascular Disease: 10.1% + General Hypertension: 6.1% + Non-Specified Cardiac Ischemia: 5.1%	Cardiac Dysrhythmia: 40.8% + Non-Specific Cardiovascular Disease: 11.5% + Non-Rheumatic Heart Disease: 7.8% + Cardiac Conduction Disorder: 3.8% + Upper Respiratory Inflammation: 3.1%
Pearson's correlation (*p*-value)		0.999 (< 10^{-16})	0.999 (< 10^{-16})	0.999 (< 10^{-16})	0.718 (< 10^{-16})
8. Cardiovascular	General Hypertension: 48.2% + Unspecified Hyperlipidemia: 14.6% + Mixed Hyperlipidemia: 2.9% + Acute Bronchitis: 2.5% + Esophageal Disease: 1.7%	General Hypertension: 50.6% + Unspecified Hyperlipidemia: 13.7% + Acute Bronchitis: 2.6% + Mixed Hyperlipidemia: 2.4% + Esophageal Disease: 1.7%	General Hypertension: 51.7% + Unspecified Hyperlipidemia: 14.9% + Acute Bronchitis: 2.7% + Mixed Hyperlipidemia: 2.2% + Obesity: 1.6%	General Hypertension: 49.9% + Unspecified Hyperlipidemia: 14.1% + Acute Bronchitis: 2.6% + Mixed Hyperlipidemia: 2.5% + Esophageal Disease: 1.5%	General Hypertension: 59.0% + Unspecified Lipid Metabolism Disorder: 7.2% + Osteoarthritis: 4.5% + Pure Hypercholesterolemia: 3.0% + Upper Respiratory Inflammation: 1.8%
Pearson's correlation (*p*-value)		0.999 (< 10^{-16})	0.999 (< 10^{-16})	0.999 (< 10^{-16})	0.943 (< 10^{-16})
9. Brain	Cerebrovascular Disease: 21.2% + Epilepsy Related Disorders: 7.3% + Multiple Sclerosis Other Demyelinating Disease: 5.3% + Unspecified Encephalopathy: 3.0% + Brain Damage: 2.6%	Cerebrovascular Disease: 23.1% + Epilepsy Related Disorders: 7.4% + Extrapyramidal Abnormal Movement Disorders: 3.4% + Unspecified Encephalopathy: 3.0% + Brain Damage: 2.8%	Cerebrovascular Disease: 27.6% + Epilepsy Related Disorders: 5.0% + Extrapyramidal Abnormal Movement Disorders: 2.6% + Parkinson's Disease: 2.5% + Unspecified Encephalopathy: 2.4%	Cerebrovascular Disease: 19.9% + Multiple Sclerosis Other Demyelinating Disease: 7.5% + Epilepsy Related Disorders: 5.8% + Unspecified Encephalopathy: 2.6% + Brain Damage: 2.2%	Cerebrovascular Disease: 24.7% + Epilepsy Related Disorders: 14.9% + Hemiplegia: 4.0% + Unspecified Recurrent Headaches: 2.8% + Non-Specific Acquired Musculoskeletal Abnormality: 2.8%
Pearson's correlation (*p*-value)		0.970 (< 10^{-16})	0.952 (< 10^{-16})	0.985 (< 10^{-16})	0.900 (< 10^{-16})
10. Thyroid	Acquired Hypothyroidism: 36.4% + Goiter: 9.2% + Vitamin Deficiency: 6.0% + Unspecified Hyperlipidemia: 4.8% + Menopausal Disorder: 2.7%	Acquired Hypothyroidism: 36.0% + Goiter: 8.5% + Vitamin Deficiency: 5.6% + Unspecified Hyperlipidemia: 5.0% + Menopausal Disorder: 2.7%	Acquired Hypothyroidism: 40.7% + Goiter: 8.8% + Unspecified Hyperlipidemia: 5.9% + Vitamin Deficiency: 4.5% + Thyrotoxicosis: 2.2%	Acquired Hypothyroidism: 36.5% + Goiter: 8.7% + Vitamin Deficiency: 6.0% + Unspecified Hyperlipidemia: 4.4% + Menopausal Disorder: 2.6%	Acquired Hypothyroidism: 54.8% + Thyrotoxicosis: 4.5% + Disease of the Female Reproductive Organs: 3.2% + Osteoarthritis: 2.3% + Upper Respiratory Inflammation: 1.9%

Pearson's correlation (*p*-value)		0.999 (< 10^{-16})	0.997 (< 10^{-16})	0.999 (< 10^{-16})	0.942 (< 10^{-16})
11. Psychiatric	Depression: 57.7% + Anxiety Phobic Disorder: 21.0% + PTSD: 3.4% + Non-Specific Pain: 0.5% + Acute Upper Respiratory Infection: 0.3%	Depression: 58.4% + Anxiety Phobic Disorder: 20.4% + PTSD: 1.1% + Non-Specific Pain: 0.5% + Non-Specific Joint Disorder: 0.4%	Depression: 55.7% + Anxiety Phobic Disorder: 18.3% + PTSD: 3.0% + ADHD: 0.7% + Non-Specific Pain: 0.6%	Depression: 60.0% + Anxiety Phobic Disorder: 19.7% + PTSD: 1.2% + Non-Specific Pain: 0.5% + Adjustment Disorder: 0.4%	Substance Abuse: 42.2% + Depression: 11.4% + Anxiety Phobic Disorder: 4.0% + Non-Specific Gastrointestinal Disorder: 2.9% + Bipolar Disorder: 2.3%
Pearson's correlation (*p*-value)		0.999 (< 10^{-16})	0.999 (< 10^{-16})	0.999 (< 10^{-16})	0.272 (4.38×10^{-11})
Extra. Allergic Rhinitis	Allergic Rhinitis: 94.1% + Acute Sinusitis: 0.8% + Eye Inflammation: 0.8% + Chronic Sinusitis: 0.7% + Atopic Contact Dermatitis: 0.3%	Allergic Rhinitis: 94.4% + Acute Sinusitis: 0.8% + Eye Inflammation: 0.8% + Chronic Sinusitis: 0.5% + Atopic Contact Dermatitis: 0.3%	Allergic Rhinitis: 93.6% + Acute Sinusitis: 0.8% + Eye Inflammation: 0.7% + Chronic Sinusitis: 0.6% + Atopic Contact Dermatitis: 0.3%	Allergic Rhinitis: 94.2% + Acute Sinusitis: 0.9% + Chronic Sinusitis: 0.7% + Eye Inflammation: 0.7% + Atopic Contact Dermatitis: 0.3%	
Pearson's correlation (*p*-value)		0.999 (< 10^{-16})	0.999 (< 10^{-16})	0.999 (< 10^{-16})	
Extra. Anemia	Non-Specific Anemia: 32.2% + Chronic Kidney Disease: 24.3% + General Hypertension: 4.0% + Electrolyte Acid Base Balance Disorder: 3.9% + Acute Renal Failure: 2.4%	Non-Specific Anemia: 32.2% + Chronic Kidney Disease: 22.4% + Electrolyte Acid Base Balance Disorder: 4.2% + General Hypertension: 4.1% + Non-Specific Cardiovascular Disease: 2.5%	Non-Specific Anemia: 33.1% + Chronic Kidney Disease: 27.5% + General Hypertension: 4.4% + Electrolyte Acid Base Balance Disorder: 4.3% + Acute Renal Failure: 2.9%	Non-Specific Anemia: 30.9% + Chronic Kidney Disease: 24.5% + General Hypertension: 4.1% + Electrolyte Acid Base Balance Disorder: 3.8% + Acute Renal Failure: 2.5%	
Pearson's correlation (*p*-value)		0.999 (< 10^{-16})	0.999 (< 10^{-16})	0.995 (< 10^{-16})	
Extra. Breast Cancer	Breast Cancer: 50.0% + Secondary Malignant Neoplasm: 6.3% + Breast Disorder: 5.4% + Lymphatic Disorder: 1.7% + Unspecified White Blood Cell Disease: 1.4%	Breast Cancer: 52.9% + Secondary Malignant Neoplasm: 6.1% + Breast Disorder: 6.0% + Lymphatic Disorder: 2.0% + Unspecified White Blood Cell Disease: 1.4%	Breast Cancer: 32.5% + Lung Cancer: 14.9% + Secondary Malignant Neoplasm: 9.3% + Unspecified Cancer: 2.7% + Breast Disorder: 2.2%	Breast Cancer: 51.6% + Secondary Malignant Neoplasm: 6.2% + Breast Disorder: 4.9% + Lymphatic Disorder: 1.8% + Unspecified White Blood Cell Disease: 1.3%	
Pearson's correlation (*p*-value)		0.999 (< 10^{-16})	0.902 (< 10^{-16})	0.999 (< 10^{-16})	
Extra. Lung Cancer	Lung Cancer: 19.7% + Secondary Malignant Neoplasm: 10.9% + Colorectal Cancer: 10.4% + Unspecified Cancer: 4.9% + Non-Specific Pulmonary Disorder: 3.4%	Lung Cancer: 17.8% + Secondary Malignant Neoplasm: 10.5% + Colorectal Cancer: 9.5% + Unspecified Cancer: 4.7% + Oro-Naso-Pharyngeal Cancer: 3.9%		Lung Cancer: 23.3% + Secondary Malignant Neoplasm: 11.0% + Colorectal Cancer: 10.2% + Oro-Naso-Pharyngeal Cancer: 5.1% + Unspecified Cancer: 4.8%	
Pearson's correlation (*p*-value)		0.990 (< 10^{-16})		0.992 (< 10^{-16})	
Extra. Headache	Migraine: 38.1% + Unspecified Recurrent Headaches: 5.0% + Non-Specific Pain: 3.5% + Muscle Ligament Disorder: 2.3% + Acquired Visual Disturbances: 1.8%	Migraine: 38.9% + Non-Specific Pain: 5.7% + Unspecified Recurrent Headaches: 5.1% + Muscle Ligament Disorder: 2.4% + Extrapyramidal Abnormal Movement Disorders: 1.9%		Migraine: 44.5% + Unspecified Recurrent Headaches: 5.2% + Non-Specific Pain: 4.9% + Muscle Ligament Disorder: 2.8% + Acquired Visual Disturbances: 1.8%	
Pearson's correlation (*p*-value)		0.997 (< 10^{-16})		0.998 (< 10^{-16})	

Extra. Pain	Non-Specific Pain: 54.6% + General Spondylosis Spine Disorder: 26.4% + Muscle Ligament Disorder: 5.8% + Spinal Stenosis: 2.9% + Non-Specific Joint Disorder: 1.9%	Non-Specific Pain: 54.3% + General Spondylosis Spine Disorder: 26.9% + Muscle Ligament Disorder: 4.6% + Spinal Stenosis: 3.0% + Non-Specific Joint Disorder: 1.8%	Non-Specific Pain: 51.8% + General Spondylosis Spine Disorder: 28.3% + Muscle Ligament Disorder: 3.6% + Spinal Stenosis: 3.6% + Non-Specific Joint Disorder: 2.1%	Non-Specific Pain: 53.9% + General Spondylosis Spine Disorder: 26.5% + Muscle Ligament Disorder: 5.2% + Spinal Stenosis: 3.0% + Non-Specific Joint Disorder: 2.0%	
Pearson's correlation (*p*-value)		0.999 ($< 10^{-16}$)	0.998 ($< 10^{-16}$)	0.999 ($< 10^{-16}$)	
Extra. Sleep Apnea	Sleep Apnea: 59.0% + Obesity: 17.0% + General Hypertension: 3.1% + Sleep Disorder: 2.0% + Esophageal Disease: 1.5%	Sleep Apnea: 60.2% + Obesity: 16.7% + General Hypertension: 2.6% + Sleep Disorder: 2.0% + Esophageal Disease: 1.6%	Sleep Apnea: 66.7% + Obesity: 13.2% + General Hypertension: 2.3% + Sleep Disorder: 2.0% + Esophageal Disease: 1.3%	Sleep Apnea: 61.1% + Obesity: 14.9% + General Hypertension: 2.6% + Sleep Disorder: 1.8% + Esophageal Disease: 1.4%	
Pearson's correlation (*p*-value)		0.999 ($< 10^{-16}$)	0.996 ($< 10^{-16}$)	0.999 ($< 10^{-16}$)	
Extra. Male Reproductive	Disease of the Male Reproductive Organs: 18.3% + Urinary Calculus: 13.6% + Non-Specific Urinary Disorder: 12.3% + Benign Prostatic Hyperplasia: 8.8% + Prostate Cancer: 6.1%	Disease of the Male Reproductive Organs: 17.4% + Urinary Calculus: 13.8% + Non-Specific Urinary Disorder: 12.9% + Benign Prostatic Hyperplasia: 8.4% + UTI: 5.2%	Disease of the Male Reproductive Organs: 16.4% + Urinary Calculus: 13.1% + Non-Specific Urinary Disorder: 12.1% + Benign Prostatic Hyperplasia: 11.1% + UTI: 3.3%	Disease of the Male Reproductive Organs: 18.3% + Urinary Calculus: 14.8% + Non-Specific Urinary Disorder: 12.1% + Benign Prostatic Hyperplasia: 8.9% + UTI: 3.5%	
Pearson's correlation (*p*-value)		0.994 ($< 10^{-16}$)	0.972 ($< 10^{-16}$)	0.977 ($< 10^{-16}$)	
Extra. Upper Respiratory	Acute Sinusitis: 12.7% + Acute Bronchitis: 9.7% + Acute Upper Respiratory Infection: 8.1% + Upper Respiratory Inflammation: 7.6% + Allergic Rhinitis: 5.5%	Acute Sinusitis: 14.1% + Chronic Sinusitis: 13.4% + Acute Bronchitis: 10.2% + Acute Upper Respiratory Infection: 7.8% + Upper Respiratory Inflammation: 7.5%	Chronic Sinusitis: 14.8% + Acute Sinusitis: 14.3% + Acute Bronchitis: 10.3% + Allergic Rhinitis: 6.9% + Upper Respiratory Inflammation: 5.8%	Acute Sinusitis: 13.5% + Acute Bronchitis: 9.7% + Chronic Sinusitis: 8.2% + Acute Upper Respiratory Infection: 7.9% + Upper Respiratory Inflammation: 7.5%	
Pearson's correlation (*p*-value)		0.900 ($< 10^{-16}$)	0.848 ($< 10^{-16}$)	0.965 ($< 10^{-16}$)	
Extra. Skin Disorder	Non-Specific Skin Disorder: 18.6% + Cellulitis: 15.4% + Fungal Infection: 8.4% + Peripheral Vascular Disease: 6.1% + Nail Disease: 4.9%		Non-Specific Skin Disorder: 18.8% + Cellulitis: 12.7% + Peripheral Vascular Disease: 6.8% + Fungal Infection: 5.1% + Non-Specific Vascular Disease: 4.2%	Non-Specific Skin Disorder: 16.2% + Cellulitis: 12.7% + Fungal Infection: 5.0% + Peripheral Vascular Disease: 4.5% + Nail Disease: 2.8%	
Pearson's correlation (*p*-value)			0.984 ($< 10^{-16}$)	0.990 ($< 10^{-16}$)	
Extra. Skin Cancer	Keratosis: 7.7% + Non-Melanoma Skin Cancer: 6.4% + Benign Skin Neoplasm: 5.2% + Cataract: 4.5% + Non-Specific Skin Disorder: 3.4%	Keratosis: 9.5% + Non-Melanoma Skin Cancer: 7.8% + Benign Skin Neoplasm: 6.5% + Non-Specific Skin Disorder: 4.8% + Atopic Contact Dermatitis: 4.2%	Keratosis: 15.5% + Non-Melanoma Skin Cancer: 12.2% + Benign Skin Neoplasm: 8.8% + Atopic Contact Dermatitis: 6.0% + Non-Specific Skin Disorder: 5.8%	Keratosis: 12.2% + Non-Melanoma Skin Cancer: 11.0% + Benign Skin Neoplasm: 9.0% + Non-Specific Skin Disorder: 5.5% + Unspecified Hyperlipidemia: 2.3%	
Pearson's correlation (*p*-value)		0.908 ($< 10^{-16}$)	0.919 ($< 10^{-16}$)	0.938 ($< 10^{-16}$)	
Extra. Female Reproductive		Disease of the Female Reproductive Organs: 24.1% + Gestational Pregnancy Related Disorder: 17.7% + Menstrual	Disease of the Female Reproductive Organs: 17.8% + Menstrual Disorder: 6.9% + Menopausal Disorder: 6.5% +	Disease of the Female Reproductive Organs: 24.1% + Gestational Pregnancy Related Disorder: 18.2% + Menstrual	Disease of the Female Reproductive Organs: 29.0% + Gestational Pregnancy Related Disorder: 11.9% +

		Disorder: 12.1% + Breast Disorder: 3.7% + Benign Uterine Neoplasm: 3.7%	Breast Disorder: 5.8% + UTI: 4.1%	Disorder: 12.5% + Breast Disorder: 3.7% + Benign Uterine Neoplasm: 3.5%	Menstrual Disorder: 7.2% + Benign Uterine Neoplasm: 3.8% + Menopausal Disorder: 3.1%
Extra. Substance Abuse		Substance Abuse: 55.2% + Anxiety Phobic Disorder: 3.4% + Acute Bronchitis: 3.3% + Non-Specific Pain: 2.6% + Depression: 2.2%	Substance Abuse: 29.8% + Viral Hepatitis C: 7.8% + Chronic Liver Disease: 7.6% + Pancreatic Disease: 3.5% + Biliary Tract Disease: 2.8%		
Extra. Glaucoma		Glaucoma: 17.7% + Cataract: 15.3% + Eye Inflammation: 9.3% + Acquired Visual Disturbances: 5.5% + Acquired Retinal Defects: 5.0%	Glaucoma: 17.8% + Cataract: 16.6% + Eye Inflammation: 6.1% + Acquired Retinal Defects: 4.8% + Acquired Visual Disturbances: 4.7%	Glaucoma: 17.9% + Cataract: 15.2% + Eye Inflammation: 7.8% + Acquired Visual Disturbances: 5.4% + Acquired Retinal Defects: 4.8%	
Extra. Adjustment Disorder		Adjustment Disorder: 89.1% + Depression: 1.2% + Anxiety Phobic Disorder: 1.0% + Acute Upper Respiratory Infection: 0.5% + Upper Respiratory Inflammation: 0.4%		Adjustment Disorder: 89.5% + Depression: 1.1% + Anxiety Phobic Disorder: 1.1% + Acute Upper Respiratory Infection: 0.5% + Upper Respiratory Inflammation: 0.5%	
Extra. Bipolar Disorder				Bipolar Disorder: 36.3% + Substance Abuse: 3.8% + Depression: 2.6% + Anxiety Phobic Disorder: 2.2% + Schizophrenia Related Psychosis: 1.4%	
Extra. Secondary Cancer					Secondary Malignant Neoplasm: 42.3% + Unspecified Cancer: 1.6% + Unspecified White Blood Cell Disease: 1.0% + Pleuritis: 0.5% + Septicemia: 0.5%
Extra. Cataract					Cataract: 31.0% + Acquired Retinal Defects: 13.3% + Glaucoma: 11.2% + Acquired Visual Disturbances: 3.9% + General Hypertension: 3.4%
Extra. Urinary Disorder					Non-Specific Urinary Disorder: 24.6% + Benign Prostatic Hyperplasia: 10.1% + Bladder Disorder: 9.5% + Urinary Calculus: 8.8% + Prostate Cancer: 7.8%
Extra. Biliary Tract Disease					Biliary Tract Disease: 40.3% + Pancreatic Disease: 12.1% + Disease of the Female

					Reproductive Organs: 6.4% + Non-Specific Gastrointestinal Disorder: 4.4% + Chronic Liver Disease: 3.9%
Extra. Cardiac Ischemia					Non-Specified Cardiac Ischemia: 48.6% + General Hypertension: 11.5% + Myocardial Infarction: 11.2% + Unspecified Lipid Metabolism Disorder: 8.2% + Upper Respiratory Inflammation: 7.0%
Extra. Chronic Kidney Disease					Chronic Kidney Disease: 79.1% + Non-Specific Nephropathy: 1.8% + General Hypertension: 1.4% + Acute Renal Failure: 0.7% + Non-Specific Anemia: 0.4%

[a] We applied topic modeling to five different cohorts (see [b-f]), generating a total of 33 subgroups (see Methods "The US MarketScan Commercial database and topic modeling for asthma subgroup identification"). Out of these subgroups, we particularly numbered the eleven stable subgroups that can be commonly found in all the five cohorts and thus were further discussed in this study (see the first eleven rows), while named the other 22 subgroups as "Extra" subgroups (see the last 22 rows).

[b] We applied topic modeling to a population of 6,048,247 asthma patients aged between 15 and 70 in the US MarketScan insurance claims database, identifying a total of 22 subgroups (see the filled rows). Here, we report the top five most abundant comorbid diseases therein contained as well as their respective occurring frequencies (see Supplementary Data 1 for the complete subgroup profiles).

[c] The same topic modeling procedure was applied to a population of 3,152,519 individuals in the US MarketScan data who were aged between 15 and 70, but carried at least two asthma codes. This sensitivity analysis 1 generated a total of 25 subgroups, of which 21 subgroups had been seen among the subgroups found in the discovery cohort [b] (see Supplementary Data 2 for the complete subgroup profiles).

[d] The same topic modeling procedure was applied to a population of 3,401,250 individuals in the US MarketScan data who carried at least one asthma code, but were aged between 40 and 70. This sensitivity analysis 2 generated a total of 23 subgroups, of which 20 subgroups had been seen among the subgroups found in the discovery cohort [b] (see Supplementary Data 3 for the complete subgroup profiles).

[e] The same topic modeling procedure was also applied to a population of 3,687,965 individuals in the US MarketScan data who not only were aged between 15 and 70 and carried at least one asthma code, but also had at least one type of asthma drug prescriptions. This sensitivity analysis 3 generated a total of 26 subgroups, of which 22 subgroups had been seen among the subgroups found in the discovery cohort [b] (see Supplementary Data 4 for the complete subgroup profiles).

[f] The same topic modeling procedure was also applied to a population of 66,448 individuals enrolled in UK Biobank who carried at least one asthma code and were aged between 39 and 72. This sensitivity analysis 4 generated a total of 18 subgroups, of which eleven subgroups had been seen among the subgroups found in the discovery cohort [b] (see Supplementary Data 5 for the complete subgroup profiles).

[g] In order to assess whether any of the subgroups generated based on the cohorts for sensitivity analyses can be claimed as successful replications of the subgroups discovered based on the discovery cohort, we computed their Pearson's correlations based on the median frequency profiles of comorbid diseases in the respective subgroups. We only claim a successful replication, if the respective correlation is determined to be significant. The Pearson's correlation coefficients and their corresponding two-sided *p*-values out of Student's *t* tests are shown here.

Supplementary Table 3. Pathway analysis based on GWAS summary statistics (see Methods "Pathway enrichment analysis based on GWAS summary statistics").

**Asthma subgroups**	**Enriched canonical pathways/ Biological processes (Benjamini–Hochberg adjusted *p*-value)**	**Overlap with GWAS catalog association signals**
1. Diabetes	Ubiquitin-Proteasomal pathway involving Parkin (1.17×10^{-3});<br>Dopamine transport (5.48×10^{-3});<br>Positive regulation of response to oxidative stress (5.48×10^{-3});<br>Alpha-Synuclein signaling (2.86×10^{-2}).	Aging (1.24×10^{-2});<br>Loneliness (2.84×10^{-2}).
2. Autoimmune	Phase I biotransformations (2.94×10^{-2}).	Lipoprotein (a) levels (2.39×10^{-4});<br>Sarcoidosis (non-Lofgren's syndrome without extrapulmonary manifestations) (3.33×10^{-4});<br>Heel bone mineral density (6.67×10^{-3});<br>Hyperopia (2.03×10^{-2});<br>Ossification of the posterior longitudinal ligament of the spine (2.93×10^{-2});<br>Dimensional psychopathology (Negative) (2.93×10^{-2});<br>Post bronchodilator FEV1 (3.73×10^{-2});<br>Periodontitis (4.23×10^{-2}).
3. GI	Keratinocyte differentiation (7.52×10^{-16});<br>Regulation of leukocyte proliferation (5.27×10^{-7});<br>IL12-mediated signaling (8.43×10^{-7});<br>Leukocyte differentiation (9.05×10^{-7});<br>Regulation of lymphocyte differentiation (1.31×10^{-6});<br>STAT5 signaling in response to IL2 stimulation (4.87×10^{-6});<br>Interferon gamma response (5.11×10^{-6});<br>Regulation of B cell activation (1.18×10^{-5});<br>Regulation of T cell differentiation (1.61×10^{-5});<br>JAK-STAT signaling pathway (1.16×10^{-3});<br>Th1/Th2 Differentiation (3.02×10^{-3});<br>Late response to estrogen (3.13×10^{-3});<br>IL4-mediated signaling (4.16×10^{-3});<br>IL5 signaling pathway (9.85×10^{-3});<br>IL6 Jak STAT3 signaling (1.32×10^{-2});<br>NF-kB signaling in response to TNF (2.55×10^{-2}).	Asthma or allergic disease (2.77×10^{-49});<br>Ulcerative colitis (1.43×10^{-12});<br>Eosinophil counts (2.06×10^{-12});<br>Nasal polyps (5.51×10^{-9});<br>Neutrophil percentage of granulocytes (3.91×10^{-7});<br>Lymphocyte counts (6.78×10^{-3}).
4. Lymphoma	Late response to estrogen (5.30×10^{-3}).	Waist-to-hip ratio adjusted for BMI (age >50) (2.81×10^{-2});<br>Systemic juvenile idiopathic arthritis (3.04×10^{-2}).
5. Musculoskeletal	NA	Urinary magnesium-to-creatinine ratio (1.51×10^{-7});<br>Chronic obstructive pulmonary disease or resting heart rate (pleiotropy) (1.72×10^{-6});<br>Calcium levels (1.01×10^{-3});<br>Loneliness (1.87×10^{-3});<br>Metabolite levels (5-HIAA) (7.16×10^{-3}).

6. Lung	NA	Fast beta electroencephalogram (3.40×10^{-9});<br>Ulcerative colitis (3.18×10^{-2}).
7. Heart	Amino acid conjugation of benzoic acid (3.38×10^{-4});<br>Glycine metabolic process (7.34×10^{-3}).	Severe aortic features in Marfan syndrome (2.92×10^{-3});<br>Heschl's gyrus morphology (4.23×10^{-3});<br>Basophil percentage of white cells (2.26×10^{-2});<br>Basophil percentage of granulocytes (2.26×10^{-2});<br>Periodontal microbiota (2.26×10^{-2}).
8. Cardiovascular	Keratinocyte differentiation (4.40×10^{-19});<br>Epidermal cell differentiation (2.24×10^{-14});<br>Leukocyte differentiation (1.76×10^{-9});<br>STAT5 signaling in response to IL2 stimulation (2.87×10^{-8});<br>Lymphocyte activation (1.74×10^{-7});<br>Regulation of B cell activation (9.52×10^{-6});<br>IL12-mediated signaling (2.75×10^{-5});<br>Positive regulation of type 2 immune response (3.11×10^{-5});<br>T-helper 1 type immune response (9.98×10^{-5});<br>Regulation of T cell differentiation (2.56×10^{-4});<br>Positive regulation of macrophage activation (2.56×10^{-4});<br>Late response to estrogen (6.17×10^{-4});<br>IL4-mediated signaling (1.20×10^{-3});<br>Development and heterogeneity of the ILC family (1.73×10^{-3});<br>Th1/Th2 Differentiation (2.82×10^{-3});<br>Positive regulation of immunoglobulin production (7.89×10^{-3});<br>Apoptosis mediated by caspase cascade (8.09×10^{-3});<br>Cellular response to interferon gamma (8.92×10^{-3});<br>Caspase Cascade in Apoptosis (9.12×10^{-3});<br>IL5 signaling (9.40×10^{-3});<br>IL6 Jak STAT3 signaling (1.99×10^{-2}).	Asthma or allergic disease (pleiotropy) (4.74×10^{-42});<br>Eosinophil counts (4.13×10^{-14});<br>Eosinophil percentage of granulocytes (9.71×10^{-13});<br>Nasal polyps (2.79×10^{-9});<br>Neutrophil percentage of granulocytes (1.34×10^{-8});<br>IgE levels (3.38×10^{-7});<br>Rheumatoid arthritis (4.45×10^{-7}).
9. Brain	Amine ligand-binding receptors signaling (7.07×10^{-3}).	Rhegmatogenous retinal detachment (1.75×10^{-13});<br>Congenital left-sided heart lesions (4.33×10^{-4});<br>Body mass index (6.79×10^{-4});<br>Melanoma (1.85×10^{-3});<br>Bipolar disorder (8.29×10^{-3});<br>Blood osmolality (transformed sodium) (8.42×10^{-3}).
10. Thyroid	NA	Lung adenocarcinoma (4.68×10^{-3});<br>Skin pigmentation (7.76×10^{-3}).
11. Psychiatric	Positive regulation of chondrocyte differentiation (4.84×10^{-3});<br>Positive regulation of cartilage development (9.05×10^{-3}) ;<br>ErbB1 internalization pathway (2.19×10^{-2}).	Lipoprotein (a) levels (9.30×10^{-4});<br>Dimensional psychopathology (Negative) (2.15×10^{-2});<br>Vitamin D levels (2.15×10^{-2}).

Supplementary Table 4. Basic subgroup-specific information (based on US MarketScan and UK Biobank data).

Basic info.	Subgroups	US MarketScan [a]		UK Biobank (white British) [b]	
		Case	Control	Case	Control
Sample size (percentage of total) [c]	1. Diabetes	268,627 (4.4%)	3,245,822 (4.1%)	1,140 (2.6%)	4,464 (1.7%)
	2. Autoimmune	31,046 (0.5%)	319,349 (0.4%)	562 (1.3%)	2,936 (1.1%)
	3. GI	377,025 (6.2%)	4,739,945 (6.1%)	9,041 (20.4%)	49,738 (19.1%)
	4. Lymphoma	19,760 (0.3%)	285,338 (0.4%)	368 (0.8%)	2,241 (0.9%)
	5. Musculoskeletal	643,760 (10.6%)	8,359,443 (10.7%)	3,020 (6.8%)	21,161 (8.1%)
	6. Lung	147,698 (2.4%)	559,456 (0.7%)	1,461 (3.3%)	2,589 (1.0%)
	7. Heart	162,336 (2.7%)	1,991,044 (2.5%)	2,670 (6.0%)	13,300 (5.1%)
	8. Cardiovascular	538,939 (8.9%)	7,425,887 (9.5%)	8,557 (19.3%)	50,508 (19.4%)
	9. Brain	53,340 (0.9%)	875,865 (1.1%)	1,296 (2.9%)	8,149 (3.1%)
	10. Thyroid	203,593 (3.4%)	2,828,415 (3.6%)	2,093 (4.7%)	11,793 (4.5%)
	11. Psychiatric	270,950 (4.5%)	3,439,181 (4.4%)	949 (2.1%)	5,892 (2.3%)
Male percentage	1. Diabetes	37.4%	56.0%	57.5%	66.6%
	2. Autoimmune	17.2%	27.1%	31.0%	33.4%
	3. GI	36.2%	47.4%	41.6%	45.0%
	4. Lymphoma	44.6%	52.1%	54.6%	55.8%
	5. Musculoskeletal	34.5%	48.3%	38.9%	43.5%
	6. Lung	42.0%	61.6%	49.1%	51.4%
	7. Heart	55.2%	67.0%	63.5%	67.0%
	8. Cardiovascular	41.2%	54.7%	48.2%	54.1%
	9. Brain	38.4%	49.7%	47.7%	51.8%
	10. Thyroid	13.2%	18.0%	13.2%	14.0%
	11. Psychiatric	28.5%	38.2%	31.7%	33.8%
Median age [d]	1. Diabetes	53 (46-60)	53 (45-60)	61 (55-66)	61 (56-65)
	2. Autoimmune	52 (42-59)	50 (39-58)	61 (54-65)	59 (52-64)
	3. GI	42 (30-52)	41 (29-51)	58 (51-63)	59 (51-63)
	4. Lymphoma	50 (36-59)	46 (32-57)	60 (52-65)	60 (52-64)
	5. Musculoskeletal	44 (30-54)	42 (29-52)	57 (49-62)	58 (51-63)
	6. Lung	57 (48-63)	47 (30-59)	60 (53-64)	58 (50-63)
	7. Heart	56 (47-62)	55 (44-62)	62 (57-66)	62 (56-66)
	8. Cardiovascular	51 (43-58)	51 (43-58)	61 (55-65)	61 (55-65)
	9. Brain	49 (34-59)	47 (31-58)	58 (51-64)	59 (52-64)
	10. Thyroid	46 (36-54)	45 (35-54)	59 (52-64)	59 (52-64)
	11. Psychiatric	36 (24-47)	34 (25-45)	51 (45-58)	53 (46-59)

[a] The MarketScan insurance claims database in US;

[b] The white British subset of UK Biobank;

[c] The percentage values do not sum up to 100%, because there are other eleven subgroups discovered based on US MarketScan data but not found in UK Biobank and they are not shown here;

[d] Values in parentheses are interquartile ranges given in years and they are the ages when individuals were enrolled.

Supplementary Table 5. Subgroup-specific white blood cell counts (based on UK Biobank data).

White blood cell count (10^9 cells/liter) [a]	Subgroups	UK Biobank (white British) [b]	
		Case	Control
Eosinophil count	1. Diabetes	0.2 (0.1-0.3)	0.17 (0.1-0.25)
	2. Autoimmune	0.17 (0.1-0.26)	0.11 (0.09-0.2)
	3. GI	0.2 (0.1-0.3)	0.13 (0.1-0.2)
	4. Lymphoma	0.15 (0.1-0.285)	0.11 (0.09-0.2)
	5. Musculoskeletal	0.19 (0.1-0.28)	0.12 (0.1-0.2)
	6. Lung	0.2 (0.1-0.3)	0.14 (0.1-0.2)
	7. Heart	0.2 (0.1-0.3)	0.14 (0.1-0.21)
	8. Cardiovascular	0.2 (0.1-0.3)	0.14 (0.1-0.21)
	9. Brain	0.2 (0.1-0.3)	0.12 (0.1-0.2)
	10. Thyroid	0.2 (0.1-0.3)	0.14 (0.1-0.2)
	11. Psychiatric	0.19 (0.1-0.29)	0.12 (0.1-0.2)
Neutrophil count	1. Diabetes	5 (3.99-6.1)	4.5 (3.67-5.5)
	2. Autoimmune	4.72 (3.7-5.96)	4.2 (3.4-5.3)
	3. GI	4.27 (3.4-5.3)	4 (3.29-4.9)
	4. Lymphoma	3.935 (3.092-5.058)	3.82 (3.007-4.86)
	5. Musculoskeletal	4.1 (3.3-5.04)	3.9 (3.2-4.8)
	6. Lung	4.6 (3.68-5.95)	4.12 (3.33-5.12)
	7. Heart	4.575 (3.65-5.69)	4.2 (3.4-5.1)
	8. Cardiovascular	4.5 (3.67-5.54)	4.23 (3.48-5.17)
	9. Brain	4.31 (3.4-5.4)	4.1 (3.3-5.06)
	10. Thyroid	4.265 (3.47-5.3)	3.99 (3.212-4.89)
	11. Psychiatric	4.3 (3.4-5.3)	4 (3.268-4.98)
Lymphocyte count	1. Diabetes	2.04 (1.6-2.547)	2 (1.6-2.47)
	2. Autoimmune	1.79 (1.4-2.22)	1.72 (1.37-2.17)
	3. GI	1.88 (1.5-2.3)	1.87 (1.5-2.28)
	4. Lymphoma	1.7 (1.3-2.238)	1.7 (1.27-2.2)
	5. Musculoskeletal	1.88 (1.52-2.29)	1.85 (1.51-2.24)
	6. Lung	1.895 (1.5-2.3)	1.86 (1.5-2.3)
	7. Heart	1.81 (1.5-2.3)	1.82 (1.5-2.25)
	8. Cardiovascular	1.9 (1.52-2.32)	1.9 (1.53-2.3)
	9. Brain	1.9 (1.5-2.33)	1.83 (1.5-2.26)
	10. Thyroid	1.92 (1.59-2.4)	1.9 (1.56-2.3)
	11. Psychiatric	1.9 (1.56-2.35)	1.86 (1.5-2.25)
Monocyte count	1. Diabetes	0.5 (0.4-0.63)	0.5 (0.4-0.61)
	2. Autoimmune	0.46 (0.38-0.6)	0.44 (0.35-0.56)
	3. GI	0.46 (0.37-0.59)	0.44 (0.36-0.56)
	4. Lymphoma	0.45 (0.37-0.6)	0.44 (0.33-0.58)
	5. Musculoskeletal	0.45 (0.36-0.56)	0.43 (0.35-0.54)

	6. Lung	0.5 (0.4-0.62)	0.45 (0.37-0.58)
	7. Heart	0.5 (0.4-0.65)	0.5 (0.4-0.6)
	8. Cardiovascular	0.5 (0.4-0.6)	0.5 (0.4-0.6)
	9. Brain	0.49 (0.39-0.6)	0.47 (0.38-0.6)
	10. Thyroid	0.46 (0.37-0.58)	0.43 (0.35-0.54)
	11. Psychiatric	0.43 (0.34-0.56)	0.42 (0.34-0.53)
Basophil count	1. Diabetes	0.03 (0-0.06)	0.02 (0-0.05)
	2. Autoimmune	0.03 (0-0.05)	0.02 (0-0.04)
	3. GI	0.02 (0-0.05)	0.02 (0-0.04)
	4. Lymphoma	0.02 (0-0.05)	0.02 (0-0.04)
	5. Musculoskeletal	0.02 (0-0.05)	0.02 (0-0.04)
	6. Lung	0.03 (0-0.06)	0.02 (0-0.04)
	7. Heart	0.03 (0-0.05)	0.02 (0-0.04)
	8. Cardiovascular	0.03 (0-0.05)	0.02 (0-0.04)
	9. Brain	0.02 (0-0.05)	0.02 (0-0.04)
	10. Thyroid	0.03 (0-0.05)	0.02 (0-0.04)
	11. Psychiatric	0.03 (0-0.05)	0.02 (0-0.04)

[a] The assay was performed on blood samples which were obtained during UK Biobank assessment center visit. Eosinophil count in the table, for example, is the median proportion of (eosinophils/100) × white blood cell count given in 10^9 cells/liter (*i.e.*, unit of measurement here), and the values in parentheses are interquartile ranges;

[b] The white British subset of UK Biobank.

Supplementary Table 6. Subgroup-specific spirometry measurements (based on UK Biobank data).

Spirometry	Subgroups	UK Biobank (white British) [a]	
		Case	Control
FVC [b]	1. Diabetes	0.793 (0.695-0.907)	0.853 (0.752-0.944)
	2. Autoimmune	0.865 (0.76-0.975)	0.911 (0.811-1.007)
	3. GI	0.886 (0.781-0.987)	0.928 (0.832-1.022)
	4. Lymphoma	0.877 (0.785-0.981)	0.92 (0.825-1.013)
	5. Musculoskeletal	0.901 (0.805-0.998)	0.934 (0.842-1.026)
	6. Lung	0.831 (0.712-0.938)	0.895 (0.79-0.999)
	7. Heart	0.83 (0.719-0.948)	0.895 (0.796-0.994)
	8. Cardiovascular	0.857 (0.749-0.959)	0.902 (0.805-0.999)
	9. Brain	0.865 (0.745-0.967)	0.905 (0.805-1.006)
	10. Thyroid	0.868 (0.77-0.971)	0.912 (0.819-1.006)
	11. Psychiatric	0.898 (0.79-0.994)	0.934 (0.84-1.023)
FEV_1 [c]	1. Diabetes	0.765 (0.642-0.885)	0.854 (0.746-0.96)
	2. Autoimmune	0.826 (0.689-0.929)	0.898 (0.788-1.002)
	3. GI	0.844 (0.723-0.956)	0.919 (0.814-1.02)
	4. Lymphoma	0.827 (0.717-0.958)	0.911 (0.809-1.006)
	5. Musculoskeletal	0.864 (0.75-0.968)	0.926 (0.825-1.024)
	6. Lung	0.746 (0.563-0.893)	0.869 (0.738-0.98)
	7. Heart	0.774 (0.642-0.897)	0.889 (0.776-0.994)
	8. Cardiovascular	0.815 (0.686-0.933)	0.9 (0.792-1.004)
	9. Brain	0.812 (0.681-0.926)	0.897 (0.78-1)
	10. Thyroid	0.836 (0.712-0.945)	0.906 (0.805-1.003)
	11. Psychiatric	0.851 (0.737-0.957)	0.917 (0.819-1.012)
PEF [d]	1. Diabetes	0.739 (0.582-0.879)	0.78 (0.622-0.918)
	2. Autoimmune	0.799 (0.639-0.921)	0.812 (0.662-0.937)
	3. GI	0.808 (0.664-0.943)	0.826 (0.682-0.955)
	4. Lymphoma	0.808 (0.646-0.949)	0.816 (0.678-0.951)
	5. Musculoskeletal	0.828 (0.689-0.956)	0.841 (0.698-0.966)
	6. Lung	0.714 (0.529-0.878)	0.776 (0.619-0.926)
	7. Heart	0.75 (0.583-0.891)	0.813 (0.661-0.953)
	8. Cardiovascular	0.787 (0.634-0.92)	0.815 (0.664-0.947)
	9. Brain	0.763 (0.596-0.899)	0.79 (0.623-0.927)
	10. Thyroid	0.813 (0.662-0.944)	0.819 (0.678-0.95)
	11. Psychiatric	0.797 (0.656-0.92)	0.817 (0.676-0.944)
FEV_1/FVC [e]	1. Diabetes	0.96 (0.886-1.019)	1.011 (0.954-1.055)
	2. Autoimmune	0.94 (0.861-1.001)	0.982 (0.932-1.03)
	3. GI	0.952 (0.881-1.01)	0.992 (0.94-1.037)
	4. Lymphoma	0.946 (0.887-1.005)	0.993 (0.939-1.04)
	5. Musculoskeletal	0.957 (0.888-1.011)	0.991 (0.941-1.036)

	6. Lung	0.899 (0.775-0.979)	0.971 (0.902-1.027)
	7. Heart	0.932 (0.849-1.003)	0.995 (0.939-1.042)
	8. Cardiovascular	0.955 (0.879-1.014)	1 (0.947-1.046)
	9. Brain	0.942 (0.861-1.004)	0.99 (0.934-1.041)
	10. Thyroid	0.957 (0.89-1.011)	0.991 (0.941-1.033)
	11. Psychiatric	0.953 (0.877-1.004)	0.983 (0.933-1.029)

[a] The white British subset of UK Biobank;

[b] FVC stands for forced vital capacity, and we report its fraction of predicted FVC value here (see Methods "Associating with health-related phenotypes based on UKB phenotypic data");

[c] FEV_1 stands for forced expiratory volume in one second, and we report its fraction of predicted FEV_1 value here;

[d] PEF stands for peak expiratory flow, and we report its fraction of predicted PEF value here;

[e] FEV_1/FVC is the ratio of FEV_1 to FVC, and we report its fraction of predicted ratio value here.

Supplementary Table 7. Other subgroup-specific information (based on UK Biobank data).

Other info.	Subgroups	UK Biobank (white British) [a]	
		Case	Control
Median BMI [b]	1. Diabetes	32.194 (28.393-36.192)	30.407 (27.391-34.22)
	2. Autoimmune	27.21 (24.223-30.575)	26.194 (23.592-29.304)
	3. GI	27.144 (24.455-30.586)	26.587 (24.096-29.548)
	4. Lymphoma	26.641 (23.899-29.929)	26.32 (23.872-29.361)
	5. Musculoskeletal	27.346 (24.674-30.938)	26.677 (24.202-29.688)
	6. Lung	26.016 (23.193-29.131)	25.599 (23.147-28.506)
	7. Heart	27.748 (24.849-31.643)	27.215 (24.715-30.309)
	8. Cardiovascular	29.059 (26.02-32.911)	28.282 (25.602-31.531)
	9. Brain	26.684 (23.984-30.589)	26.542 (23.948-29.49)
	10. Thyroid	28.173 (24.958-32.45)	26.892 (24.162-30.469)
	11. Psychiatric	26.796 (24.083-30.261)	26.048 (23.452-29.029)
Smoking status [c] (percentages of previous, current smokers)	1. Diabetes	47.0%, 16.1%	45.4%, 11.2%
	2. Autoimmune	40.6%, 14.0%	38.0%, 11.1%
	3. GI	37.2%, 12.4%	35.9%, 10.1%
	4. Lymphoma	39.3%, 12.2%	33.7%, 9.4%
	5. Musculoskeletal	36.3%, 9.3%	34.1%, 9.2%
	6. Lung	41.6%, 18.9%	37.2%, 16.1%
	7. Heart	45.1%, 16.1%	42.7%, 9.6%
	8. Cardiovascular	42.1%, 10.3%	39.7%, 8.0%
	9. Brain	35.8%, 19.8%	35.5%, 12.9%
	10. Thyroid	37.6%, 9.9%	36.2%, 7.5%
	11. Psychiatric	30.0%, 19.9%	31.1%, 14.8%
Pack years adult smoking as proportion of life span exposed to smoking [d]	1. Diabetes	0.741 (0.422-1.124)	0.585 (0.333-0.917)
	2. Autoimmune	0.541 (0.312-0.868)	0.449 (0.231-0.75)
	3. GI	0.53 (0.269-0.878)	0.447 (0.228-0.741)
	4. Lymphoma	0.593 (0.278-0.95)	0.445 (0.236-0.712)
	5. Musculoskeletal	0.462 (0.225-0.75)	0.413 (0.214-0.712)
	6. Lung	0.692 (0.384-0.984)	0.531 (0.25-0.882)
	7. Heart	0.624 (0.348-0.978)	0.491 (0.25-0.797)
	8. Cardiovascular	0.565 (0.288-0.883)	0.456 (0.237-0.75)
	9. Brain	0.692 (0.381-1)	0.5 (0.262-0.838)
	10. Thyroid	0.498 (0.26-0.798)	0.407 (0.204-0.688)
	11. Psychiatric	0.5 (0.245-0.9)	0.484 (0.238-0.781)

[a] The white British subset of UK Biobank;

[b] BMI stands for body mass index, and it was constructed from height (in meters) and weight (in kilograms) measured during the initial Assessment Centre visit. Here we report the median BMI value given in kilogram/meter^2, and the values in parentheses are interquartile ranges;

[c] The two percentage values in each cell are the percentages of the participants, who smoked previously (have stopped now) and are still smoking now, respectively;

[d] This is defined as pack years of smoking divided by the number of years between the age of participants at recruitment and the age of 16.

Supplementary Table 8. Age of patients with asthma diagnosis by subgroup (based on UK Biobank data).

Subgroups	Asthma patients in UK Biobank (white British) [a]
1. Diabetes	39 (12-50)
2. Autoimmune	38 (20-50)
3. GI	35 (14-48)
4. Lymphoma	35 (13.5-50)
5. Musculoskeletal	33 (14-45)
6. Lung	33 (12-50)
7. Heart	40 (13-50.75)
8. Cardiovascular	40 (16-50)
9. Brain	32 (12-47)
10. Thyroid	39 (22-50)
11. Psychiatric	30 (14-41)

[a] For asthma patients in the white British subset of UK Biobank, we report their median ages of asthma onset (given in years) as well as interquartile ranges in parentheses.

Supplementary Table 9. Fraction of asthma patients in each subgroup who take different types of asthma medications (based on US MarketScan data).

Subgroups	Asthma patient counts	Antibody inhibitor	Inhaled corticosteroids	Inhaled steroid combinations with long acting beta agonists	Leukotriene modifiers	Mast cell stabilizers	Methylxanthines	Short-acting, inhaled beta-2 agonists	Systemic corticosteroids
1. Diabetes	164,793	3.75×10^{-3}	0.195	0.448	0.313	7.34×10^{-4}	2.49×10^{-2}	0.693	0.665
2. Autoimmune	21,723	3.82×10^{-3}	0.177	0.421	0.298	4.14×10^{-4}	1.78×10^{-2}	0.616	0.886
3. GI	214,425	3.65×10^{-3}	0.198	0.392	0.304	7.09×10^{-4}	1.19×10^{-2}	0.689	0.632
4. Lymphoma	12,572	1.99×10^{-3}	0.183	0.416	0.277	6.36×10^{-4}	1.61×10^{-2}	0.631	0.780
5. Musculoskeletal	392,786	2.86×10^{-3}	0.197	0.390	0.304	6.90×10^{-4}	1.14×10^{-2}	0.711	0.664
6. Lung	102,070	6.54×10^{-3}	0.175	0.702	0.360	1.44×10^{-3}	1.21×10^{-1}	0.651	0.835
7. Heart	100,456	3.21×10^{-3}	0.190	0.469	0.288	6.87×10^{-4}	2.62×10^{-2}	0.627	0.718
8. Cardiovascular	324,961	2.11×10^{-3}	0.189	0.419	0.297	4.22×10^{-4}	1.55×10^{-2}	0.691	0.661
9. Brain	30,644	2.45×10^{-3}	0.166	0.403	0.274	1.01×10^{-3}	1.94×10^{-2}	0.627	0.675
10. Thyroid	121,880	2.42×10^{-3}	0.195	0.391	0.318	7.30×10^{-4}	1.09×10^{-2}	0.713	0.633
11. Psychiatric	171,793	3.17×10^{-3}	0.221	0.386	0.293	6.46×10^{-4}	7.93×10^{-3}	0.739	0.606

Supplementary Table 10. Summary statistics of the first and the second genetic principal components of asthma cases and non-asthma controls in each subgroup (based on UK Biobank data).

The first genetic principal component (PC1)							
Asthma subgroups		Min.	1st Qu.	Median	Mean	3rd Qu.	Max.
1. Diabetes	Case	-17.865	-13.485	-12.466	-12.397	-11.293	-6.935
	Control	-18.108	-13.425	-12.379	-12.347	-11.305	-6.828
2. Autoimmune	Case	-16.775	-13.391	-12.173	-12.207	-11.121	-6.638
	Control	-18.317	-13.371	-12.367	-12.336	-11.268	-6.241
3. GI	Case	-18.071	-13.475	-12.409	-12.391	-11.340	-6.564
	Control	-18.357	-13.462	-12.396	-12.380	-11.324	-6.137
4. Lymphoma	Case	-16.52	-13.32	-12.24	-12.22	-11.04	-6.78
	Control	-16.929	-13.417	-12.327	-12.311	-11.285	-6.567
5. Musculoskeletal	Case	-17.51	-13.42	-12.35	-12.33	-11.28	-6.72
	Control	-18.206	-13.424	-12.371	-12.341	-11.287	-6.053
6. Lung	Case	-18.414	-13.457	-12.413	-12.389	-11.333	-6.969
	Control	-18.367	-13.398	-12.392	-12.326	-11.281	-6.381
7. Heart	Case	-17.672	-13.465	-12.395	-12.372	-11.312	-6.502
	Control	-18.436	-13.461	-12.403	-12.357	-11.311	-6.209
8. Cardiovascular	Case	-17.725	-13.436	-12.394	-12.364	-11.319	-6.254
	Control	-18.33	-13.44	-12.37	-12.34	-11.29	-6.18
9. Brain	Case	-17.094	-13.467	-12.391	-12.353	-11.387	-6.965
	Control	-18.336	-13.483	-12.383	-12.366	-11.297	-6.145
10. Thyroid	Case	-17.328	-13.431	-12.308	-12.279	-11.163	-6.578
	Control	-18.049	-13.470	-12.415	-12.386	-11.358	-6.218
11. Psychiatric	Case	-17.925	-13.345	-12.357	-12.261	-11.149	-7.284
	Control	-18.064	-13.371	-12.342	-12.304	-11.290	-6.435

The second genetic principal component (PC2)							
		Min.	1st Qu.	Median	Mean	3rd Qu.	Max.
1. Diabetes	Case	-0.5595	2.7788	3.7357	3.7868	4.8066	8.4672
	Control	-1.431	2.773	3.807	3.783	4.791	8.826
2. Autoimmune	Case	-1.972	2.783	3.875	3.776	4.869	8.089
	Control	-1.515	2.844	3.796	3.772	4.737	8.708
3. GI	Case	-1.981	2.764	3.804	3.785	4.792	9.255
	Control	-1.882	2.769	3.778	3.765	4.785	9.319
4. Lymphoma	Case	0.02846	2.91168	3.72756	3.81476	4.73132	8.17107
	Control	-1.324	2.677	3.732	3.710	4.716	8.376
5. Musculoskeletal	Case	-1.261	2.834	3.853	3.811	4.806	9.105
	Control	-1.839	2.776	3.791	3.775	4.793	9.427
6. Lung	Case	-1.049	2.810	3.826	3.776	4.735	9.353
	Control	-1.329	2.773	3.800	3.785	4.836	8.581
7. Heart	Case	-1.746	2.743	3.767	3.765	4.795	9.181
	Control	-2.024	2.785	3.808	3.783	4.790	8.703
8. Cardiovascular	Case	-1.759	2.786	3.807	3.793	4.823	9.179
	Control	-1.781	2.792	3.808	3.793	4.808	9.165
9. Brain	Case	-0.8662	2.7806	3.8118	3.7601	4.7527	7.9944
	Control	-1.506	2.808	3.830	3.792	4.781	8.710
10. Thyroid	Case	-0.9042	2.7570	3.8081	3.7905	4.8342	9.3455
	Control	-1.889	2.773	3.780	3.778	4.788	8.873
11. Psychiatric	Case	-1.766	2.699	3.799	3.779	4.831	8.609
	Control	-1.563	2.762	3.783	3.782	4.818	8.895

Supplementary Table 11. Summary statistics of individuals' diagnosis code counts in each subgroup and in these subgroups combined (based on US MarketScan data).

Asthma subgroups	Min.	1st Qu.	Median	Mean	3rd Qu.	Max.
1. Diabetes	1	30	66	97.6	129	2268
2. Autoimmune	1	33	74	106.2	143	1612
3. GI	1	11	25	43.64	54	1890
4. Lymphoma	1	29	83	120.2	169	1391
5. Musculoskeletal	1	16	39	65.79	85	2077
6. Lung	1	15	54	95.27	133	2033
7. Heart	1	26	68	106.6	145	2179
8. Cardiovascular	1	15	34	52.49	69	1911
9. Brain	1	19	50	87.46	115	1760
10. Thyroid	1	15	34	51.1	67	1420
11. Psychiatric	1	18	45	77.59	100	1727
Combined subgroups	1	16	40	68.6	88	2268

Supplementary Table 12. Similarities between the distributions of subgroup (or these subgroups combined) individuals' diagnosis code counts (based on US MarketScan data).

Comparisons between two asthma subgroups	Similarity estimated using MarketScan data [a]
1. Diabetes vs. 2. Autoimmune	0.8861
1. Diabetes vs. 3. GI	0.4980
1. Diabetes vs. 4. Lymphoma	0.7488
1. Diabetes vs. 5. Musculoskeletal	0.6900
1. Diabetes vs. 6. Lung	0.7389
1. Diabetes vs. 7. Heart	0.8432
1. Diabetes vs. 8. Cardiovascular	0.6073
1. Diabetes vs. 9. Brain	0.8028
1. Diabetes vs. 10. Thyroid	0.6010
1. Diabetes vs. 11. Psychiatric	0.7665
2. Autoimmune vs. 3. GI	0.4477
2. Autoimmune vs. 4. Lymphoma	0.8296
2. Autoimmune vs. 5. Musculoskeletal	0.6235
2. Autoimmune vs. 6. Lung	0.7413
2. Autoimmune vs. 7. Heart	0.8659
2. Autoimmune vs. 8. Cardiovascular	0.5433
2. Autoimmune vs. 9. Brain	0.7555
2. Autoimmune vs. 10. Thyroid	0.5371
2. Autoimmune vs. 11. Psychiatric	0.7008
3. GI vs. 4. Lymphoma	0.4119
3. GI vs. 5. Musculoskeletal	0.7341
3. GI vs. 6. Lung	0.5404
3. GI vs. 7. Heart	0.4936

3. GI vs. 8. Cardiovascular	0.8122
3. GI vs. 9. Brain	0.5927
3. GI vs. 10. Thyroid	0.8070
3. GI vs. 11. Psychiatric	0.6524
4. Lymphoma vs. 5. Musculoskeletal	0.5609
4. Lymphoma vs. 6. Lung	0.7606
4. Lymphoma vs. 7. Heart	0.8430
4. Lymphoma vs. 8. Cardiovascular	0.4814
4. Lymphoma vs. 9. Brain	0.7093
4. Lymphoma vs. 10. Thyroid	0.4757
4. Lymphoma vs. 11. Psychiatric	0.6353
5. Musculoskeletal vs. 6. Lung	0.6996
5. Musculoskeletal vs. 7. Heart	0.6691
5. Musculoskeletal vs. 8. Cardiovascular	0.8706
5. Musculoskeletal vs. 9. Brain	0.7938
5. Musculoskeletal vs. 10. Thyroid	0.8621
5. Musculoskeletal vs. 11. Psychiatric	0.8862
6. Lung vs. 7. Heart	0.8432
6. Lung vs. 8. Cardiovascular	0.6079
6. Lung vs. 9. Brain	0.8507
6. Lung vs. 10. Thyroid	0.6009
6. Lung vs. 11. Psychiatric	0.7811
7. Heart vs. 8. Cardiovascular	0.5788
7. Heart vs. 9. Brain	0.8312
7. Heart vs. 10. Thyroid	0.5723

7. Heart vs. 11. Psychiatric	0.7545
8. Cardiovascular vs. 9. Brain	0.6891
8. Cardiovascular vs. 10. Thyroid	0.9756
8. Cardiovascular vs. 11. Psychiatric	0.7713
9. Brain vs. 10. Thyroid	0.6820
9. Brain vs. 11. Psychiatric	0.8947
10. Thyroid vs. 11. Psychiatric	0.7636
Combined subgroups vs. 1. Diabetes	0.698
Combined subgroups vs. 2. Autoimmune	0.632
Combined subgroups vs. 3. GI	0.728
Combined subgroups vs. 4. Lymphoma	0.570
Combined subgroups vs. 5. Musculoskeletal	0.973
Combined subgroups vs. 6. Lung	0.712
Combined subgroups vs. 7. Heart	0.679
Combined subgroups vs. 8. Cardiovascular	0.858
Combined subgroups vs. 9. Brain	0.805
Combined subgroups vs. 10. Thyroid	0.848
Combined subgroups vs. 11. Psychiatric	0.896

[a] Distribution similarity is measured by overlapped area relative to each pair of distributions; the value ranges from 0 to 1. The distribution similarity metric is equal to 1 for two identical distributions and 0 for two completely dissimilar ones.

Supplementary Table 13. Summary statistics of individuals' durations (in weeks) of enrollment and actual diagnosis recordings in each subgroup and in these subgroups combined (based on US MarketScan data).

Asthma subgroups	Durations of (in weeks)	Min.	1st Qu.	Median	Mean	3rd Qu.	Max.
1. Diabetes	Enrollment	0	126	226	274.9	366	678
	Diagnosis	0	92	191	227.3	334	577
2. Autoimmune	Enrollment	4	117	209	267	365	678
	Diagnosis	0	92	189	223.1	313	574
3. GI	Enrollment	0	100	161	210.1	265	678
	Diagnosis	0	49	110	157.1	214	579
4. Lymphoma	Enrollment	0	104	209	252.3	365	678
	Diagnosis	0	72	159	206.3	304	575
5. Musculoskeletal	Enrollment	0	105	209	242.2	313	678
	Diagnosis	0	74	150	189.5	259	578
6. Lung	Enrollment	0	96	174	234	326	678
	Diagnosis	0	51	137	188.3	280	580
7. Heart	Enrollment	0	105	209	268.6	365	678
	Diagnosis	0	78	174	220.4	332	577
8. Cardiovascular	Enrollment	0	105	209	257.1	365	678
	Diagnosis	0	73	156	202.4	294	577
9. Brain	Enrollment	3	104	196	240	326	678
	Diagnosis	0	63	147	191.5	270	578
10. Thyroid	Enrollment	0	104	208	235.1	313	678
	Diagnosis	0	68	146	184.2	255	574
11. Psychiatric	Enrollment	0	104	191	228.9	313	678
	Diagnosis	0	64	142	182.4	256	576
Combined subgroups	Enrollment	0	104	208	243.5	326	678
	Diagnosis	0	68	149	192.5	269	580

Supplementary Table 14. Similarities between the distributions of subgroup (or these subgroups combined) individuals' durations (in weeks) of enrollment and actual diagnosis recordings (based on US MarketScan data).

Comparisons between two asthma subgroups	Similarity on durations (in weeks) of enrollment [a]	Similarity on durations (in weeks) of actual diagnosis recordings [a]
1. Diabetes vs. 2. Autoimmune	0.8444	0.8977
1. Diabetes vs. 3. GI	0.7065	0.6866
1. Diabetes vs. 4. Lymphoma	0.8121	0.8452
1. Diabetes vs. 5. Musculoskeletal	0.8167	0.8199
1. Diabetes vs. 6. Lung	0.7924	0.7827
1. Diabetes vs. 7. Heart	0.8937	0.9041
1. Diabetes vs. 8. Cardiovascular	0.9020	0.8811
1. Diabetes vs. 9. Brain	0.8199	0.8180
1. Diabetes vs. 10. Thyroid	0.8368	0.8125
1. Diabetes vs. 11. Psychiatric	0.8152	0.7994
2. Autoimmune vs. 3. GI	0.6626	0.6817
2. Autoimmune vs. 4. Lymphoma	0.9254	0.9002
2. Autoimmune vs. 5. Musculoskeletal	0.7311	0.7936
2. Autoimmune vs. 6. Lung	0.8001	0.7882
2. Autoimmune vs. 7. Heart	0.9007	0.8898
2. Autoimmune vs. 8. Cardiovascular	0.7968	0.8538
2. Autoimmune vs. 9. Brain	0.8668	0.8342
2. Autoimmune vs. 10. Thyroid	0.7911	0.8009
2. Autoimmune vs. 11. Psychiatric	0.7822	0.7893
3. GI vs. 4. Lymphoma	0.6819	0.7287
3. GI vs. 5. Musculoskeletal	0.8339	0.8152
3. GI vs. 6. Lung	0.7687	0.7977

3. GI vs. 7. Heart	0.7004	0.7196
3. GI vs. 8. Cardiovascular	0.7788	0.7748
3. GI vs. 9. Brain	0.7435	0.8028
3. GI vs. 10. Thyroid	0.8254	0.8372
3. GI vs. 11. Psychiatric	0.8387	0.8545
4. Lymphoma vs. 5. Musculoskeletal	0.7206	0.8041
4. Lymphoma vs. 6. Lung	0.8400	0.8544
4. Lymphoma vs. 7. Heart	0.8887	0.8999
4. Lymphoma vs. 8. Cardiovascular	0.7809	0.8684
4. Lymphoma vs. 9. Brain	0.9093	0.9035
4. Lymphoma vs. 10. Thyroid	0.7973	0.8351
4. Lymphoma vs. 11. Psychiatric	0.8005	0.8277
5. Musculoskeletal vs. 6. Lung	0.7646	0.7958
5. Musculoskeletal vs. 7. Heart	0.7704	0.8105
5. Musculoskeletal vs. 8. Cardiovascular	0.8962	0.8853
5. Musculoskeletal vs. 9. Brain	0.7666	0.8445
5. Musculoskeletal vs. 10. Thyroid	0.8853	0.9282
5. Musculoskeletal vs. 11. Psychiatric	0.8620	0.9215
6. Lung vs. 7. Heart	0.8446	0.8425
6. Lung vs. 8. Cardiovascular	0.8115	0.8514
6. Lung vs. 9. Brain	0.8986	0.8965
6. Lung vs. 10. Thyroid	0.8276	0.8266
6. Lung vs. 11. Psychiatric	0.8620	0.8311
7. Heart vs. 8. Cardiovascular	0.8526	0.8912
7. Heart vs. 9. Brain	0.8811	0.8677

7. Heart vs. 10. Thyroid	0.8251	0.8240
7. Heart vs. 11. Psychiatric	0.8191	0.8167
8. Cardiovascular vs. 9. Brain	0.8203	0.8931
8. Cardiovascular vs. 10. Thyroid	0.9044	0.9118
8. Cardiovascular vs. 11. Psychiatric	0.8772	0.8887
9. Brain vs. 10. Thyroid	0.8551	0.8903
9. Brain vs. 11. Psychiatric	0.8656	0.8927
10. Thyroid vs. 11. Psychiatric	0.9261	0.9512
Combined subgroups vs. 1. Diabetes	0.7695	0.8159
Combined subgroups vs. 2. Autoimmune	0.6753	0.7751
Combined subgroups vs. 3. GI	0.8462	0.8168
Combined subgroups vs. 4. Lymphoma	0.6695	0.7899
Combined subgroups vs. 5. Musculoskeletal	0.8943	0.9226
Combined subgroups vs. 6. Lung	0.7310	0.8096
Combined subgroups vs. 7. Heart	0.7296	0.8183
Combined subgroups vs. 8. Cardiovascular	0.8364	0.8972
Combined subgroups vs. 9. Brain	0.7126	0.8315
Combined subgroups vs. 10. Thyroid	0.8117	0.8950
Combined subgroups vs. 11. Psychiatric	0.8042	0.8962

[a] Distribution similarity is measured by overlapped area relative to each pair of distributions; the value ranges from 0 to 1. The distribution similarity metric is equal to 1 for two identical distributions and 0 for two completely dissimilar ones.

Supplementary Table 15. Two types of subgroup-specific fraction values computed based on the diagnosis records of asthma patients with at least one comorbid disease code.

Asthma subgroups	Top disease codes	Faction of patients who are in the subgroup indeed carry the top code	Fraction of patients who carry the top code are eventually assigned to the subgroup
1. Diabetes	Type II Diabetes Mellitus	0.998	0.292
2. Autoimmune	Rheumatoid Arthritis Related Conditions	0.966	0.155
3. GI	Esophageal Disease	0.773	0.205
4. Lymphoma	Non-Hodgkins Lymphoma	0.626	0.386
5. Musculoskeletal	Non-specific Joint Disorder	0.921	0.239
6. Lung	Emphysema COPD	0.907	0.156
7. Heart	Cardiac Dysrhythmia	0.719	0.181
8. Cardiovascular	General Hypertension	0.997	0.239
9. Brain	Cerebrovascular Disease	0.713	0.096
10. Thyroid	Acquired Hypothyroidism	0.956	0.218
11. Psychiatric	Depression	0.902	0.208

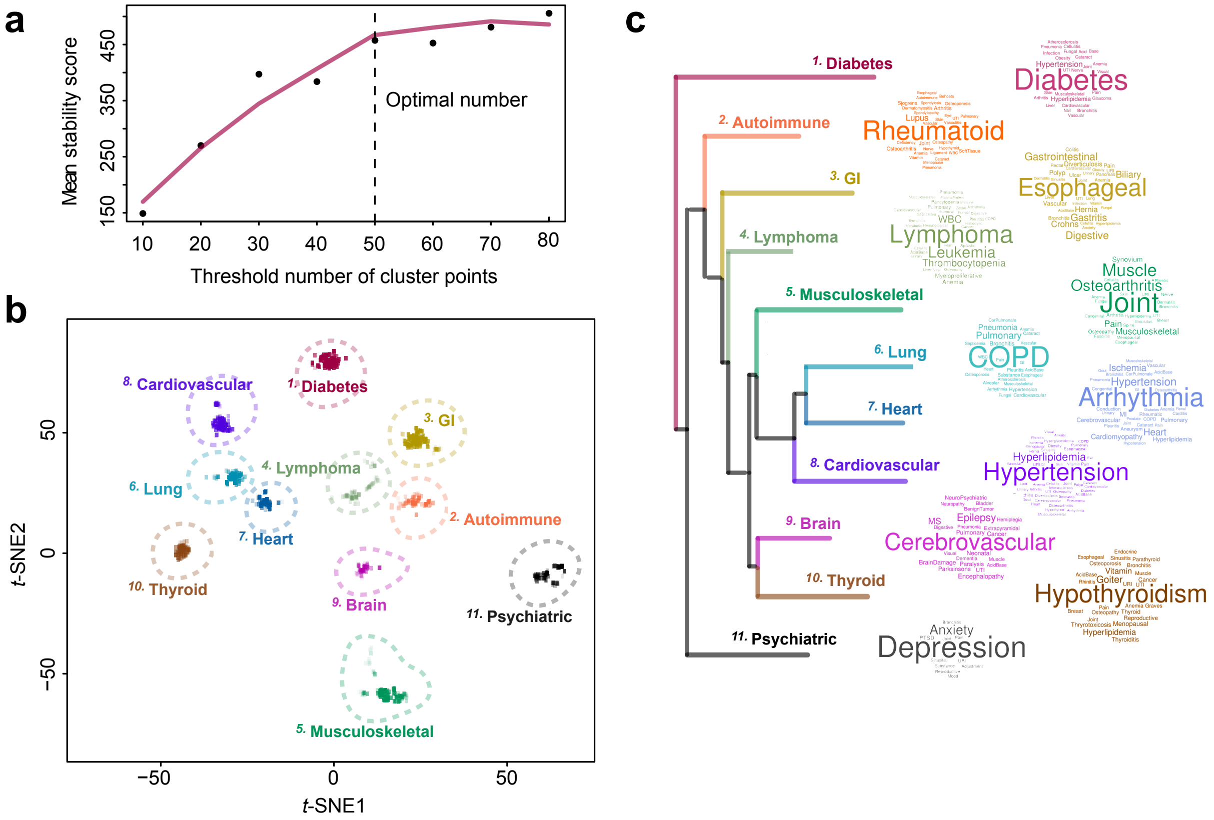

Supplementary Fig. 1. Visualizations of the identified eleven asthma subgroups (Related to Fig. 1).

(a) The elbow method determined the optimal threshold number of cluster points for claiming a stable subgroup. Varying the threshold numbers, we computed the mean stability score of the resulting subgroups for each threshold number. Here, we plot the mean stability scores (y-axis) against different threshold numbers (x-axis). The threshold number of 50 appears optimal, because it is where the increase of the mean stability score switches from fast to slow (*i.e.*, the "elbow" location, indicated by a dashed line).

(b) The *t*-SNE projection of the identified asthma subgroups. Applying the flowchart shown in Fig.1 generated eleven asthma subgroups (shown in different colors). We named each subgroup after the broader category to which several most frequently occurring diseases belonged, and also numbered the subgroups for easier reference. Singleton subgroup points were treated as noises and thus excluded from display. This two-dimensional *t*-SNE projection here is for visualization purpose only, while the actual subgrouping was done based on all the dimensions of 567 diseases (see Methods).

(c) The hierarchy of the asthma subgroups. With between-subgroup dissimilarity measured by Jensen-Shannon divergence, HDBSCAN inferred the hierarchical clustering of the subgroups, shown here as a dendrogram using the same color coding as in panel **a**. For each subgroup, a word cloud shows comorbid diseases therein contained, the occurring frequencies of which are roughly proportional to the font sizes (see Supplementary Data 1 for precise descriptions of the frequency distributions). Note that the identified asthma subgroups

are mutually exclusive, and each subgroup is defined by a unique distribution of co-occurring diseases.

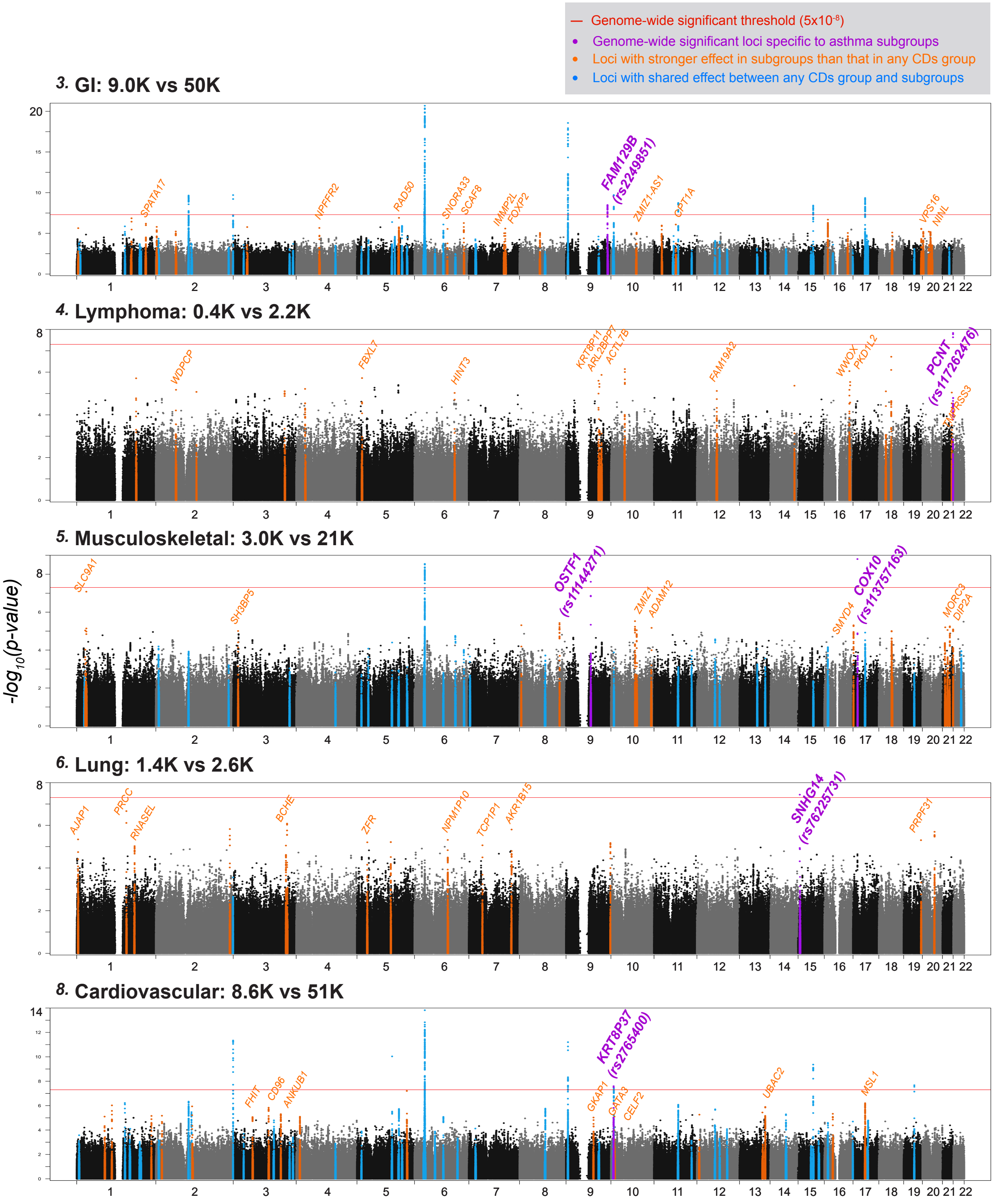

Supplementary Fig. 2. GWAS Manhattan plots with stronger-effect and shared-effect loci annotated (Related to Fig. 2b).

On the basis of UK Biobank data analysis, Manhattan plots were generated to indicate statistical significance of genetic associations between states of SNPs and asthma in individual subgroups. We selected subgroups 3 “GI,” 4 “Lymphoma,” 5 “Musculoskeletal,” 6 “Lung,” and 8

“Cardiovascular” that contained genome-wide significant loci for display here (as labelled in each plot’s title, there also shows asthma case count versus non-asthma control count in the respective subgroup). All *p*-values are shown on a $-log_{10}$ scale on the *y*-axis, and genomic locations are shown on the *x*-axis. The threshold of genome-wide significance (5×10^{-8}) is indicated as a red horizontal line in each plot. Some loci shared the similar effects in subgroups as those in the any-CDs group, and we color these loci in blue; other loci showed significantly stronger effects in subgroups (see Methods) and are marked in orange along with their nearest genes. In addition, we highlighted the genome-wide significant loci that are subgroup-specific in purple and labelled them with the nearest genes and the SNPs (in parentheses). Detailed results can be found in Supplementary Data 8-10.

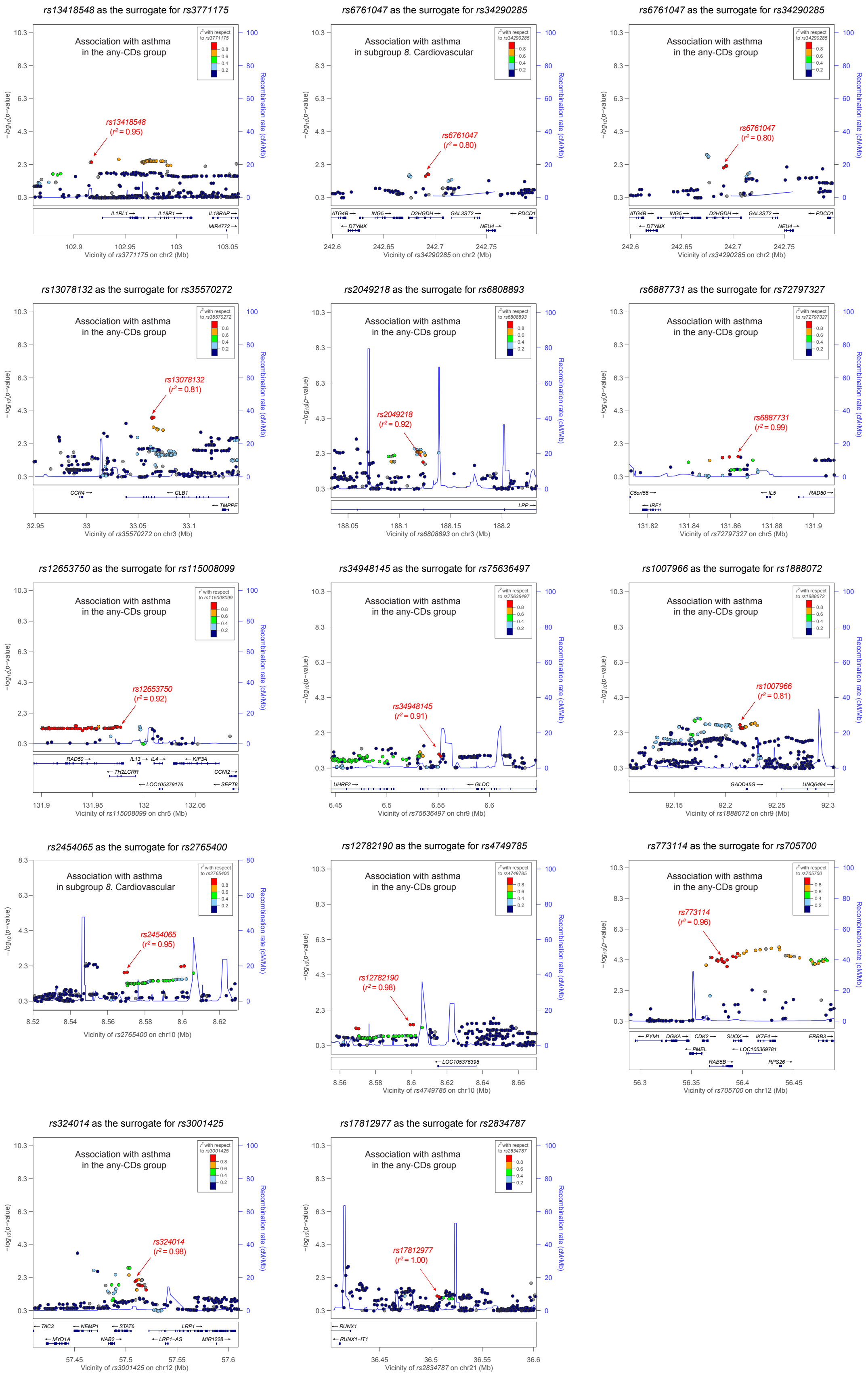
rs13418548 as the surrogate for rs3771175
Association with asthma
in the any-CDs group
rs13418548
(r^2 = 0.95)
Vicinity of rs3771175 on chr2 (Mb)
rs6761047 as the surrogate for rs34290285
Association with asthma
in subgroup 8. Cardiovascular
rs6761047
(r^2 = 0.80)
Vicinity of rs34290285 on chr2 (Mb)
rs6761047 as the surrogate for rs34290285
Association with asthma
in the any-CDs group
rs6761047
(r^2 = 0.80)
Vicinity of rs34290285 on chr2 (Mb)
rs13078132 as the surrogate for rs35570272
Association with asthma
in the any-CDs group
rs13078132
(r^2 = 0.81)
Vicinity of rs35570272 on chr3 (Mb)
rs2049218 as the surrogate for rs6808893
Association with asthma
in the any-CDs group
rs2049218
(r^2 = 0.92)
Vicinity of rs6808893 on chr3 (Mb)
rs6887731 as the surrogate for rs72797327
Association with asthma
in the any-CDs group
rs6887731
(r^2 = 0.99)
Vicinity of rs72797327 on chr5 (Mb)
rs12653750 as the surrogate for rs115008099
Association with asthma
in the any-CDs group
rs12653750
(r^2 = 0.92)
Vicinity of rs115008099 on chr5 (Mb)
rs34948145 as the surrogate for rs75636497
Association with asthma
in the any-CDs group
rs34948145
(r^2 = 0.91)
Vicinity of rs75636497 on chr9 (Mb)
rs1007966 as the surrogate for rs1888072
Association with asthma
in the any-CDs group
rs1007966
(r^2 = 0.81)
Vicinity of rs1888072 on chr9 (Mb)
rs2454065 as the surrogate for rs2765400
Association with asthma
in subgroup 8. Cardiovascular
rs2454065
(r^2 = 0.95)
Vicinity of rs2765400 on chr10 (Mb)
rs12782190 as the surrogate for rs4749785
Association with asthma
in the any-CDs group
rs12782190
(r^2 = 0.98)
Vicinity of rs4749785 on chr10 (Mb)
rs773114 as the surrogate for rs705700
Association with asthma
in the any-CDs group
rs773114
(r^2 = 0.96)
Vicinity of rs705700 on chr12 (Mb)
rs324014 as the surrogate for rs3001425
Association with asthma
in the any-CDs group
rs324014
(r^2 = 0.98)
Vicinity of rs3001425 on chr12 (Mb)
rs17812977 as the surrogate for rs2834787
Association with asthma
in the any-CDs group
rs17812977
(r^2 = 1.00)
Vicinity of rs2834787 on chr21 (Mb)
$-log_{10}$(p-value)
Recombination rate (cM/Mb)

Supplementary Fig. 3. Regional associations involving the SNPs in the vicinity of target SNPs, based on Biobank Japan (BBJ).

In BBJ, if a target SNP to be replicated was neither genotyped nor imputed, the SNP in its highest linkage disequilibrium (LD), if available, was used (see Methods). Here, we show all the relevant regional Manhattan plots, and in each plot, the left vertical axis represents $-log_{10}$ transformed *p*-value, the right vertical axis is the estimated recombination rate, and genomic locations in the vicinity of the target SNP are shown on the horizontal axis. SNPs are presented as points and colored according to their r^2 measure of LD with respect to the target SNP (pairwise r^2 values are from 1000 Genomes East Asian reference panel, March 2012 release). We chose the SNP of the highest LD as a surrogate for the target SNP, which is pointed by an arrow and annotated in red. A lower annotation track shows nearby genes along with the orientation of their transcribed strand and exons/introns (based on UCSC genome browser, Human Genome version 19).

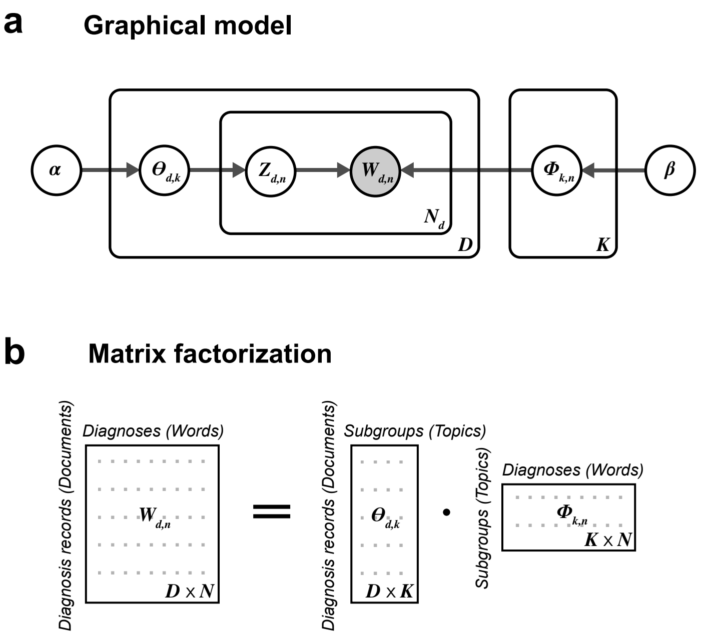

Supplementary Fig. 4. Graphical model and matrix factorization in topic modeling.

(a) Topic modeling is realized using the Hierarchical Dirichlet Process (HDP), and its basic design can be graphically presented here. Shaded and unshaded nodes indicate observed and latent variables, respectively. Arrows denote conditional dependencies between variables, and "plate" notations are used to illustrate repeated sampling steps. For example, the inner plate over $Z_{d,n}$ and $W_{d,n}$ denotes the repeated sampling of asthma subgroup assignments and diagnoses until N_d diagnoses are generated for diagnosis record d. The plate over $\Theta_{d,k}$ demonstrates the repeated sampling of a distribution over disease subgroups for each diagnosis record d for a total of D records, and the plate surrounding $\Phi_{k,n}$ illustrates the sampling of diagnosis distributions for each subgroup k until the total number K is reached. Hyperparameters α and β define the HDPs, which are distributions over a set of random probability measures over $\Theta_{d,k}$ and $\Phi_{k,n}$, respectively. Therefore, given the observed $W_{d,n}$, statistical inference aims to estimate $\Theta_{d,k}$ and $\Phi_{k,n}$.

(b) Alternatively, from the perspective of matrix factorization, this statistical inference is to find a low-dimensional representation for the record-diagnosis (document-word) co-occurrence matrix of $W_{d,n}$ by decomposing it into the matrix of subgroup (topic) proportions $\Theta_{d,k}$ and the matrix of subgroups (topics) $\Phi_{k,n}$.

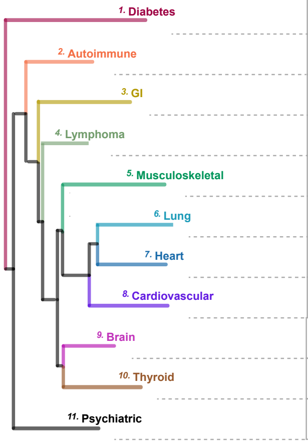

Biobank Database	US MarketScan	UK Biobank (white British)	UK Biobank (white Irish and other white)	UK Biobank (African, Caribbean and other black)	BioVU (European -descent)	Biobank Japan	UChicago RNAseq
Counts: Asthma vs non-Asthma	6M vs 78M	44K vs 261K	3186 vs 19K	998 vs 5835	1668 vs 14K	3368 vs 191K	42
1. Diabetes	0.3M vs 3.2M	1.1K vs 4.5K	90 vs 304	51 vs 254	84 vs 587	570 vs 32K	
2. Autoimmune	0.03M vs 0.3M	0.6K vs 2.9K	36 vs 174	8 vs 50	38 vs 195	66 vs 4.0K	
3. GI	0.4M vs 4.7M	9.0K vs 50K	645 vs 3717	142 vs 876	128 vs 991	74 vs 6.5K	7
4. Lymphoma	0.02M vs 0.3M	0.4K vs 2.2K	34 vs 188	5 vs 39	10 vs 286	9 vs 1.3K	
5. Musculoskeletal	0.6M vs 8.4M	3.0K vs 21K	202 vs 1461	65 vs 373	151 vs 809		5
6. Lung	0.1M vs 0.6M	1.4K vs 2.6K	124 vs 262	17 vs 39	119 vs 299	302 vs 2.3K	1
7. Heart	0.2M vs 2.0M	2.7K vs 13K	174 vs 897	23 vs 124	520 vs 5345	585 vs 37K	1
8. Cardiovascular	0.5M vs 7.4M	8.6K vs 51K	513 vs 3309	301 vs 1805	136 vs 1017	349 vs 16K	4
9. Brain	0.05M vs 0.9M	1.3K vs 8.1K	105 vs 601	24 vs 180	62 vs 803	352 vs 19K	
10. Thyroid	0.2M vs 2.8M	2.1K vs 12K	158 vs 914	31 vs 140	49 vs 325	65 vs 6.4K	3
11. Psychiatric	0.3M vs 3.4M	0.9K vs 5.9K	64 vs 507	14 vs 68	21 vs 99		

Supplementary Fig. 5. The distributions of used biobanks among asthma subgroups.

This figure summarizes all the biobank databases that were used in this study, as well as their representations among asthma subgroups. All the databases contain diagnosis record information, which we used to identify the subgroups in the case of US MarketScan dataset, or was based on to assign patients into the identified subgroups in the case of the other databases, so that various downstream analyses could follow:

(*i*) to discover subgroup-associated genetic variants and health-related phenotypes, using white British subjects in UK Biobank (that contains genome-wide genotype data and health-related phenotype data);

(*ii*) to replicate the significant genetic associations found in (*i*), by leveraging four different cohorts: a UK Biobank cohort of white Irish and any other white background, a UK Biobank cohort of African, Caribbean and any other black background, a BioVU cohort of European descent group, and a Biobank Japan cohort (that also contains genome-wide genotype data);

(*iii*) to test for subgroup-specific differential expression of the genes whose nearby SNPs were found to exhibit subgroup-specific associations with asthma, using the RNA-sequencing dataset from the University of Chicago (bronchial epithelial transcriptome profiling of 42 asthma patients).

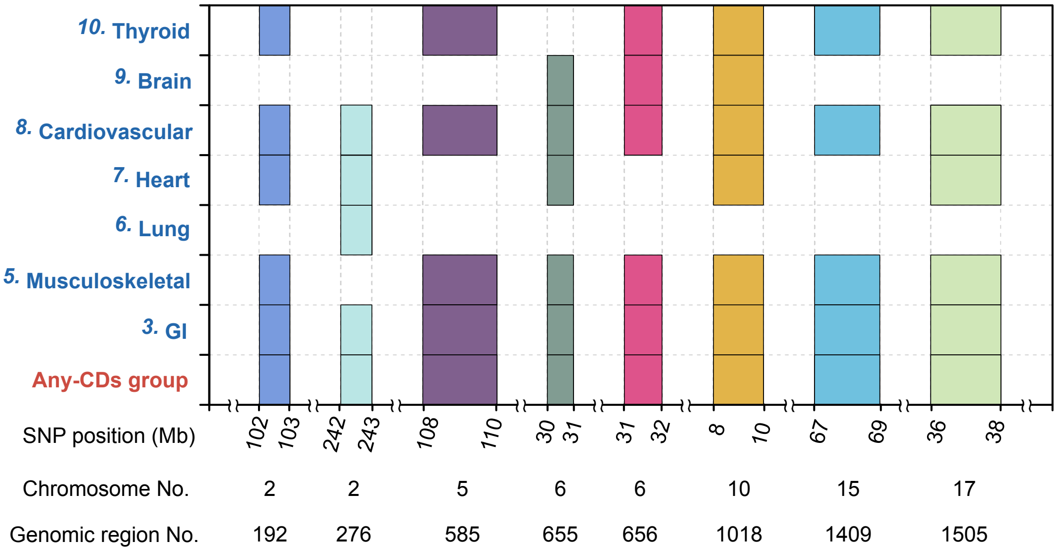

Supplementary Fig. 6. Genomic regions that share similar signals between individual asthma subgroups and the any-CDs group, and between subgroup pairs.

By analyzing GWAS summary statistics between asthma subgroups and the any-CDs group, we tested a shared-association model that describes a genomic region where there exists at least one variant that has shared influences (Methods). Vertical stripes of the same color indicate the coverages of the genomic regions (*x*-axis shows SNP positions) shared by the any-CDs group and at least four subgroups (as shown on the *y*-axis). The irrelevant regions in the genome were cropped off and omitted on the *x*-axis.

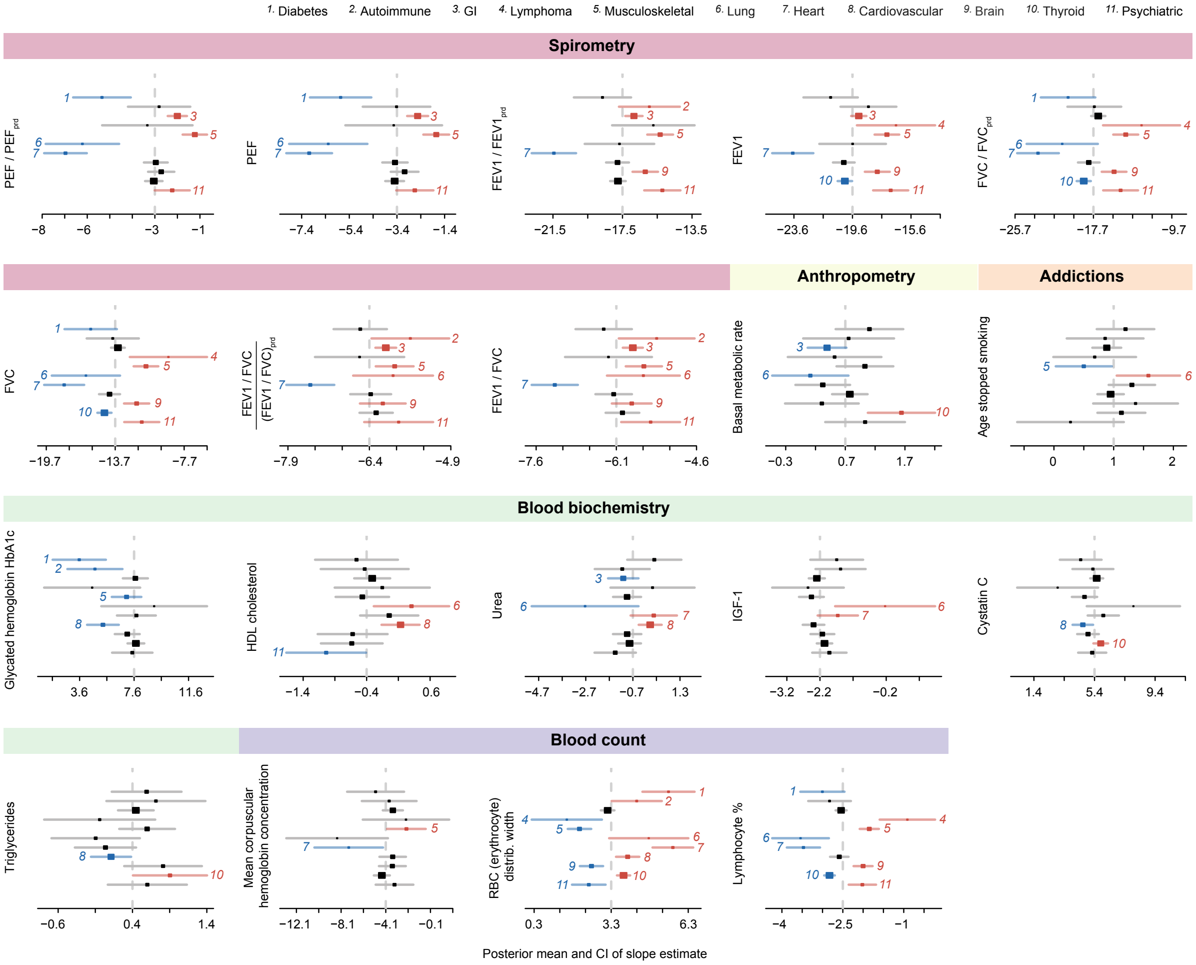

Supplementary Fig. 7. Other differential asthma associations with health-related phenotypes across subgroups (Related to Fig. 4).

The heterogeneity in the slope estimates of asthma associations was assessed across subgroups, in comparison with the slope values in the any-CDs group (served as benchmarks). In total, there are 44 phenotypes found to have differential asthma associations across subgroups, of which 25 have been displayed in Fig. 4. We show the remaining 19 phenotypes here using meta-plots. The meta-plot of a phenotype shows the posterior means (as squares) and 95 percent confidence intervals (as error bars) of the slopes from subgroups 1 to 11 (displayed from top to bottom). The benchmark slope value in the any-CDs group is marked by a vertical dashed line. If the slope estimate in a subgroup turns out to be less positive than the benchmark, we would color it in blue, and if more positive than the benchmark, we would color it in red. See Methods for technical details and Supplementary Data 11 for the numbers of allocated cases and controls in each subgroup.

Supplementary Data 1-12 (provided in separate Excel files, and the table captions are as below):

Supplementary Data 1. Asthma subgroup profile defined by comorbidities and their occurring frequencies, based on US MarketScan asthma cases (with at least one asthma code and aged between 15 and 70, for discovery analysis).

Supplementary Data 2. Asthma subgroup profile based on US MarketScan asthma cases (with at least two asthma codes and aged between 15 and 70, for sensitivity analysis 1).

Supplementary Data 3. Asthma subgroup profile based on US MarketScan asthma cases (with at least one asthma code and aged between 40 and 70, for sensitivity analysis 2).

Supplementary Data 4. Asthma subgroup profile based on US MarketScan asthma cases (with at least one asthma code, aged between 15 and 70, and with at least one type of asthma drug prescriptions, for sensitivity analysis 3).

Supplementary Data 5. Asthma subgroup profile based on UK Biobank asthma cases (with at least one asthma code, for sensitivity analysis 4).

Supplementary Data 6. Summary statistics of the identified 109 lead SNPs in asthma subgroups and in the any-CDs group, and the test results of their effect size heterogeneity (see Methods "UK Biobank (UKB) database and GWAS").

Supplementary Data 7. Summary statistics of the identified genome-wide significant associations and their replication results from multi-ancestry meta-analysis (see Methods "Replicating genome-wide significant associations in multi-ancestry meta-analysis").

Supplementary Data 8. Lead SNPs that show stronger effects in individual asthma subgroups than in the any-CDs group (see Methods "Stronger risk loci identification using a subsampling method").

Supplementary Data 9. Genomic regions that share similar effects between individual asthma subgroups and the any-CDs group (see Methods "Identifying genomic regions that share influences on asthma").

Supplementary Data 10. Genomic regions that share similar effects between asthma subgroups (see Methods "Identifying genomic regions that share influences on asthma").

Supplementary Data 11. Asthma associations with health-related phenotypes (see Methods "Associating with health-related phenotypes based on UKB phenotypic data", the first three steps of our phenotype association analysis).

Supplementary Data 12. Heterogeneity in asthma associations with health-related phenotypes across asthma subgroups (see Methods "Associating with health-related phenotypes based on UKB phenotypic data", the fourth step of our phenotype association analysis).
