# Supplementary material for: Discerning asthma endotypes through comorbidity mapping
Source: Nat Commun. 2022 Nov 7;13:6712. doi: 10.1038/s41467-022-33628-8 (PMC9640644; doi:10.1038/s41467-022-33628-8)
Supplement: Supplementary file 2 — Description of Additional Supplementary Files [file 41467_2022_33628_MOESM2_ESM.pdf]

Title: Supplementary Data 1.
Description: Asthma subgroup profile defined by comorbidities and their occurring frequencies, based on US MarketScan asthma cases (with at least one asthma code and aged between 15 and 70, for discovery analysis).

Title: Supplementary Data 2.
Description: Asthma subgroup profile based on US MarketScan asthma cases (with at least two asthma codes and aged between 15 and 70, for sensitivity analysis 1).

Title: Supplementary Data 3.
Description: Asthma subgroup profile based on US MarketScan asthma cases (with at least one asthma code and aged between 40 and 70, for sensitivity analysis 2).

Title: Supplementary Data 4.
Description: Asthma subgroup profile based on US MarketScan asthma cases (with at least one asthma code, aged between 15 and 70, and with at least one type of asthma drug prescriptions, for sensitivity analysis 3).

Title: Supplementary Data 5.
Description: Asthma subgroup profile based on UK Biobank asthma cases (with at least one asthma code, for sensitivity analysis 4).

Title: Supplementary Data 6.
Description: Summary statistics of the identified 109 lead SNPs in asthma subgroups and in the any-CDs group, and the test results of their effect size heterogeneity (see Methods “UK Biobank (UKB) database and GWAS”).

Title: Supplementary Data 7.
Description: Summary statistics of the identified genome-wide significant associations and their replication results from multi-ancestry meta-analysis (see Methods “Replicating genome-wide significant associations in multi-ancestry meta-analysis”).

Title: Supplementary Data 8.
Description: Lead SNPs that show stronger effects in individual asthma subgroups than in the any-CDs group (see Methods “Stronger risk loci identification using a subsampling method”).

Title: Supplementary Data 9.
Description: Genomic regions that share similar effects between individual asthma subgroups and the any-CDs group (see Methods “Identifying genomic regions that share influences on asthma”).

Title: Supplementary Data 10.
Description: Genomic regions that share similar effects between asthma subgroups (see Methods “Identifying genomic regions that share influences on asthma”).

Title: Supplementary Data 11.
Description: Asthma associations with health-related phenotypes (see Methods “Associating with health-related phenotypes based on UKB phenotypic data”, the first three steps of our phenotype association analysis).

Title: Supplementary Data 12.
Description: Heterogeneity in asthma associations with health-related phenotypes across asthma subgroups (see Methods “Associating with health-related phenotypes based on UKB phenotypic data”, the fourth step of our phenotype association analysis).
