# Supplementary material for: Discerning asthma endotypes through comorbidity mapping
Source: Nat Commun. 2022 Nov 7;13:6712. doi: 10.1038/s41467-022-33628-8 (PMC9640644; doi:10.1038/s41467-022-33628-8)
Supplement: Supplementary file 3 — Reporting Summary [file 41467_2022_33628_MOESM3_ESM.pdf]

nature portfolio

Corresponding author(s): Julian Solway
Andrey Rzhetsky
Carole Ober

Last updated by author(s): Aug 12, 2022

Reporting Summary

Nature Portfolio wishes to improve the reproducibility of the work that we publish. This form provides structure for consistency and transparency in reporting. For further information on Nature Portfolio policies, see our Editorial Policies and the Editorial Policy Checklist.

Statistics

For all statistical analyses, confirm that the following items are present in the figure legend, table legend, main text, or Methods section.

n/a	Confirmed	
☐	☒	The exact sample size (n) for each experimental group/condition, given as a discrete number and unit of measurement
☐	☒	A statement on whether measurements were taken from distinct samples or whether the same sample was measured repeatedly
☐	☒	The statistical test(s) used AND whether they are one- or two-sided<br>*Only common tests should be described solely by name; describe more complex techniques in the Methods section.*
☐	☒	A description of all covariates tested
☐	☒	A description of any assumptions or corrections, such as tests of normality and adjustment for multiple comparisons
☐	☒	A full description of the statistical parameters including central tendency (e.g. means) or other basic estimates (e.g. regression coefficient) AND variation (e.g. standard deviation) or associated estimates of uncertainty (e.g. confidence intervals)
☐	☒	For null hypothesis testing, the test statistic (e.g. F, t, r) with confidence intervals, effect sizes, degrees of freedom and P value noted<br>*Give P values as exact values whenever suitable.*
☒	☐	For Bayesian analysis, information on the choice of priors and Markov chain Monte Carlo settings
☒	☐	For hierarchical and complex designs, identification of the appropriate level for tests and full reporting of outcomes
☐	☒	Estimates of effect sizes (e.g. Cohen's d, Pearson's r), indicating how they were calculated

Our web collection on statistics for biologists contains articles on many of the points above.

Software and code

Policy information about availability of computer code

Data collection	No software was used.
Data analysis	The computations were done using RStudio Version 1.2.5033.<br>Genome-wide association analyses were performed using PLINK software version 2.0, which was downloaded at https://www.cog-genomics.org/plink/2.0/ and the name of software zip file was plink2_linux_x86_64_20180107.zip.<br>Hierarchical Dirichlet Process modeling was done by using a publicly available Github repository at https://github.com/blei-lab/hdp.

For manuscripts utilizing custom algorithms or software that are central to the research but not yet described in published literature, software must be made available to editors and reviewers. We strongly encourage code deposition in a community repository (e.g. GitHub). See the Nature Portfolio guidelines for submitting code & software for further information.

Data

Policy information about availability of data

All manuscripts must include a data availability statement. This statement should provide the following information, where applicable:

- Accession codes, unique identifiers, or web links for publicly available datasets
- A description of any restrictions on data availability
- For clinical datasets or third party data, please ensure that the statement adheres to our policy

Data Availability: The license of MarketScan databases is available to purchase by Federal, nonprofit, academic, pharmaceutical, and other researchers. Access to the data is contingent on completing a data use agreement and purchasing the needed license. More information about licensing the MarketScan databases can be found at https://www.ibm.com/us-en/marketplace/marketscan-research-databases. The phenotypic and genetic datasets of UK Biobank used in this study are available via the UK Biobank data access process, and the application for data access includes six steps and takes 21 weeks on average for the year of 2020 (see

https://www.ukbiobank.ac.uk/enable-your-research/apply-for-access); detailed information about the data can be found at http://www.ukbiobank.ac.uk/scientists-3/genetic-data/ and http://biobank.ctsu.ox.ac.uk/crystal/label.cgi?id=100314. Access to the phenotypic and genetic datasets of BioVU can be requested after a study proposal is received, approved by the BioVU Review Committee and a user agreement is signed. More information can be found at https://victr.vumc.org/how-to-use-biovu/. The transcriptome data of bronchial epithelial cells were deposited in the GEO (https://www.ncbi.nlm.nih.gov/geo/) under accession GSE201955. The availability about the phenotypic and genetic datasets of Biobank Japan is described at https://biobankjp.org/english/index.html, and more information can be found at https://humandbs.biosciencedbc.jp/en/hum0014-v21. The other data supporting the findings from this study are available within the manuscript and its supplementary information. Source data are provided with this paper.

Code Availability: The Hierarchical Dirichlet Process modeling was done by using a publicly available Github repository at https://github.com/blei-lab/hdp 35,36. Genome-wide association analyses were performed using PLINK software version 2.0, which was downloaded at https://www.cog-genomics.org/plink/2.0/ and the name of software zip file was plink2_linux_x86_64_20180107.zip. Statistical analyses and plotting were done using RStudio version 1.2.5033.

US MarketScan, UK Biobank, BioVU, and BioBank Japan are large-scale biomedical databases, containing in-depth health and (or) genetic information from participants. Accessing to these data requires researchers to submit research proposals and to pay access fees, because the data information is sensitive and the database is regularly maintained and augmented with additional data that need funding support.

Field-specific reporting

Please select the one below that is the best fit for your research. If you are not sure, read the appropriate sections before making your selection.

- [x] Life sciences
- [ ] Behavioural & social sciences
- [ ] Ecological, evolutionary & environmental sciences

For a reference copy of the document with all sections, see nature.com/documents/nr-reporting-summary-flat.pdf

Life sciences study design

All studies must disclose on these points even when the disclosure is negative.

Sample size	We used the largest possible samples in the databases we have for this study: 151 million individuals enrolled in the US MarketScan data, around 0.5 million individuals enrolled in the UK Biobank data, 16,060 individuals of European descent in the BioVU data, 194,413 individuals in the Biobank Japan data, and 70 individuals in the bronchoscopy studies who were enrolled in the University of Chicago hospitals. These are the largest possible sample sizes available in respective databases.
Data exclusions	No data were excluded from the analyses.
Replication	No lab experiments were performed. To computationally replicate the genome-wide significant associations discovered using the white British subset in UKB, we leveraged another four independent cohorts. Out of the 128 discovered associations (involving 109 independent loci), 127 associations (involving 108 loci) were eligible for replication, and the only one exception was due to the small sample size (i.e., none of the four cohorts had more than 100 asthma cases allocated to the subgroup). After controlling false discovery rate (FDR) using Benjamini-Hochberg procedure 138,139, we successfully replicated 61 associations (involving 52 loci, FDR < 0.10). The detailed results are summarized in Supplementary Data 7. Among the 61 associations that were successfully replicated at an overall meta-analysis FDR of 0.1, there are ten associations that have FDR values right around 0.05 (from 0.05 to 0.06) and another ten associations that have FDR values greater than 0.06. By carefully examining these 20 replication results for which FDR values fall between 0.05 and 0.1, we find different degrees of inconsistency in the direction of SNP effects found in the four replication cohorts: Compared to the effect direction found in the discovery cohort (UKB British white group), there are one, three, six, and one replications showing effects of opposite directions in UKB Irish and other white group; UKB African, Caribbean and other black group; BioVU European-descent group; and Biobank Japan group (only nine out of 20 associations have enough samples for replication attempts in the first place), respectively. Such inconsistency in effect directions would be greater for the other 66 associations that were not replicated (FDR > 0.1), particularly in the UKB black and BioVU groups which show 34 and 26 cases with inconsistent directions, respectively.
Randomization	Given stable asthma subgroups were identified, we assigned each individual to an appropriate subgroup that can best describe her/his comorbidity pattern. We purposely design our subgrouping method solely based on comorbidity pattern and not to be biased by other conventional covariates, e.g., age and gender.
Blinding	Not relevant to our study, because our study is retrospective and we assigned individuals into the subgroups that can best describe their comorbidity patterns.

Reporting for specific materials, systems and methods

We require information from authors about some types of materials, experimental systems and methods used in many studies. Here, indicate whether each material, system or method listed is relevant to your study. If you are not sure if a list item applies to your research, read the appropriate section before selecting a response.

Materials & experimental systems

n/a	Involved in the study	
☒	☐	Antibodies
☒	☐	Eukaryotic cell lines
☒	☐	Palaeontology and archaeology
☒	☐	Animals and other organisms
☐	☒	Human research participants
☒	☐	Clinical data
☒	☐	Dual use research of concern

Methods

n/a	Involved in the study	
☒	☐	ChIP-seq
☒	☐	Flow cytometry
☒	☐	MRI-based neuroimaging

Human research participants

Policy information about studies involving human research participants

Population characteristics	Age, gender, ethnicity, and genotypic information. The population characteristics of used cohorts are now reported in a subgroup-specific manner in Supplementary Table 4.
Recruitment	The US MarketScan databases, owned by IBM Watson Health, are a suite of administrative claims-based databases that include inpatient and outpatient claims, medical procedure claims, prescription claims, clinical utilization records, and healthcare expenditures. These data were collected from employers, managed care organizations, health plan providers, and state Medicaid agencies. The covered patient population is mainly composed of relatively more affluent, privately-insured segments of US society.<br>The UKB database is a National Health Service registry database in the United Kingdom, including around 500,000 participants who were aged 40–69 years and recruited between 2006 and 2010. The UK Biobank data can potentially be more prone to the population selection bias, i.e., a relatively older, healthier, and white-ancestry population.<br>Subjects participating in the bronchoscopy study had been recruited under a human subjects research protocol approved by the University of Chicago Institutional Review Board. Informed consent was obtained from all research subjects to the work involving transcriptome data of bronchial epithelial cells
Ethics oversight	IBM Watson Health, the UK Biobank Ethics and Governance Council, the Institutional Review Board at the University of Chicago.<br>The study design and conduct complied with all relevant regulations regarding the use of human study participants and was conducted in accordance with the criteria set by the Declaration of Helsinki.

Note that full information on the approval of the study protocol must also be provided in the manuscript.
